# SnAr Reactions of 2,4-Diazidopyrido[3,2-*d*]pyrimidine and Azide-Tetrazole Equilibrium Studies of the Obtained 5-Substituted Tetrazolo[1,5-*a*]pyrido[2,3-*e*]pyrimidines

**DOI:** 10.3390/molecules27227675

**Published:** 2022-11-08

**Authors:** Kristaps Leškovskis, Anatoly Mishnev, Irina Novosjolova, Māris Turks

**Affiliations:** 1 Institute of Technology of Organic Chemistry, Faculty of Materials Science and Applied Chemistry, Riga Technical University, P. Valdena str. 3, LV-1048 Riga, Latvia; 2 Latvian Institute of Organic Synthesis, Aizkraukles str. 21, LV-1006 Riga, Latvia

**Keywords:** azide, tetrazole, SnAr, triazole, CuAAC chemistry, pyrido[3,2-*d*]pyrimidine, tautomeric equilibrium, X-ray structure determination

## Abstract

A straightforward method for the synthesis of 5-substituted tetrazolo[1,5-*a*]pyrido[2,3-*e*]pyrimidines from 2,4-diazidopyrido[3,2-*d*]pyrimidine in SnAr reactions with *N*-, *O*-, and *S*- nucleophiles has been developed. The various *N*- and *S*-substituted products were obtained with yields from 47% to 98%, but the substitution with *O*-nucleophiles gave lower yields (20–32%). Furthermore, the fused tetrazolo[1,5-*a*]pyrimidine derivatives can be regarded as 2-azidopyrimidines and functionalized in copper(I)-catalyzed azide-alkyne dipolar cycloaddition (CuAAC) and Staudinger reactions due to the presence of a sufficient concentration of the reactive azide tautomer in solution. In total, seven products were fully characterized by their single crystal X-ray studies, while five of them were representatives of the tetrazolo[1,5-*a*]pyrido[2,3-*e*]pyrimidine heterocyclic system. Equilibrium constants and thermodynamic values were determined using variable temperature ^1^H NMR and are in agreement of favoring the tetrazole tautomeric form (ΔG_298_ = −3.33 to −7.52 (kJ/mol), ΔH = −19.92 to −48.02 (kJ/mol) and ΔS = −43.74 to −143.27 (J/mol·K)). The key starting material 2,4-diazidopyrido[3,2-*d*]pyrimidine presents a high degree of tautomerization in different solvents.

## 1. Introduction

Fused-pyrimidine heterocycles are privileged scaffolds that have attracted great interest due to their biological properties [1]. The modification and refinement of such scaffolds are a promising strategy for the development of novel drugs. Among them, pyrido[3,2-*d*]pyrimidine motif as a purine and pteridine analogue is a commonly used building block in drug discovery [2,3,4,5,6,7].

**Figure 1:**
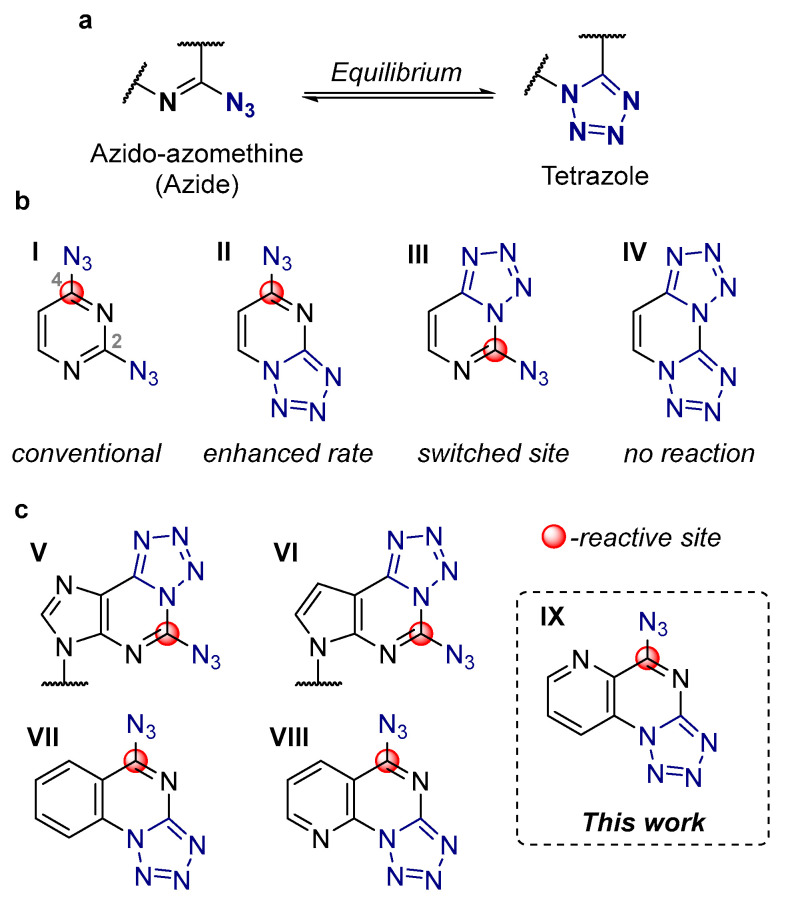
(**a**) Azide-tetrazole equilibrium; (**b**) reactivity of 2,4-diazidopyrimidines; (**c**) SnAr regioselectivity in fused 2,4-diazidopyrimidines.

From the synthesis perspective, heterocycles with an azido-azomethine structural entity are interesting due to their intrinsic dynamic azide-tetrazole tautomeric equilibrium in the solution phase (Figure 1a) [8,9,10,11,12,13,14,15] alongside rich azide functional group chemistry [16].

The azide-tetrazole equilibrium greatly varies based on the substituent electronic effects, solvent polarity, and temperature [17,18,19,20]. This phenomenon raises the opportunity to selectively substitute one position of 2,4-diazidopyrimidines (Figure 1b). Conventionally, nucleophile attack on pyrimidines takes place at a more reactive *C*-4 position when two identical leaving groups are present (Figure 1b, I). However, as the equilibrium shifts, (1) the addition rate of nucleophiles can be enhanced (Figure 1b, II) by the electron withdrawing effect of the tetrazole moiety, which stabilizes the Meisenheimer complex intermediate; (2) the addition site can be switched (Figure 1b, III), since tetrazole cannot be substituted and the substitution takes place at the *C*-2 position; and (3) addition can be completely omitted (Figure 1b, IV). Indeed, SnAr reactions in 2,4-diazidopurines V [21,22,23] and deazapurines VI [24,25] take place at the *C*-2 position (Figure 1c). However, this is not the case with quinazoline VII [26,27] and pyrido[2,3-*d*]pyrimidine VIII [28], where a conventional *C*-4 addition is observed.

To the best of our knowledge, only a handful of papers have mentioned tetrazolopyrido[2,3-*e*]pyrimidines [29,30,31,32], providing a vague idea of the azide-tetrazole tautomerism, and one paper describing the ring opening of tetrazolo[1,5-*c*]pyrido[2,3-*e*]pyrimidine with reactive *C*-nucleophiles [33]. Hence, in this paper, we report on the SnAr reactions of 2,4-diazidopyrido[3,2-*d*]pyrimidine IX for the first time, describe azide-tetrazole tautomerism in the obtained tetrazolo[1,5-*a*]pyrido[2,3-*e*]pyrimidines, further functionalize the remaining azide moiety, and provide insights into the azide-tetrazole tautomerism in 2,4-diazidopyrido[3,2-*d*]pyrimidine.

## 2. Results and Discussion

### 2.1. Synthesis

**Scheme 1:**
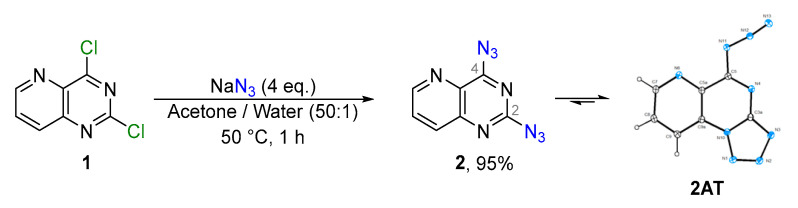
Synthesis of 2,4-diazidopyrido[3,2-*d*]pyrimidine (**2**).

First, we acquired our key starting material, 2,4-diazidopyrido[3,2-*d*]pyrimidine **2**, in excellent yield from commercially available dichloride **1** with sodium azide (Scheme 1). Here and further, the name *diazide* and structure **2** are used as formal simplification, as it does not exist in pure diazide form, but rather as a mixture of azide-tetrazole tautomeric forms.

**Scheme 2:**
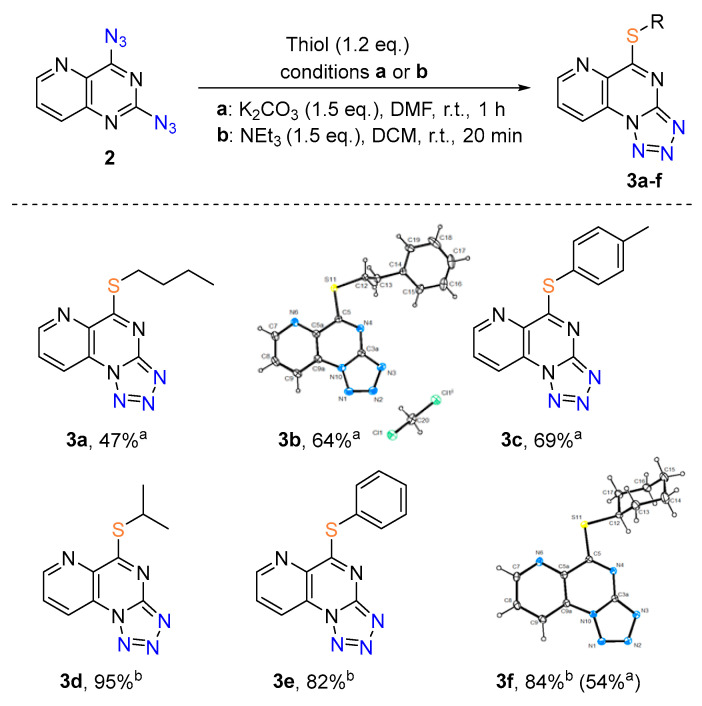
SnAr reaction of *diazide* **2** with thiols.

As the initial conditions for the substitution of *diazide* **2** with thiols, we chose the K_2_CO_3/_DMF system (Scheme 2, conditions **a**). 5-Thiotetrazolo[1,5-*a*]pyrido[2,3-*e*]pyrimidines **3a**–**c**,**f** were obtained in moderate yields with substitution proceeding at the expected *C*-4 position. These conditions were found to be most suitable in our previous work on pyrido[2,3-*d*]pyrimidines [28]. However, we discovered that the reaction could be undertaken in DCM using NEt_3_ as a base (conditions **b**). In these conditions, the work-up was easier and products **3d**–**f** were obtained in higher yields.

**Scheme 3:**
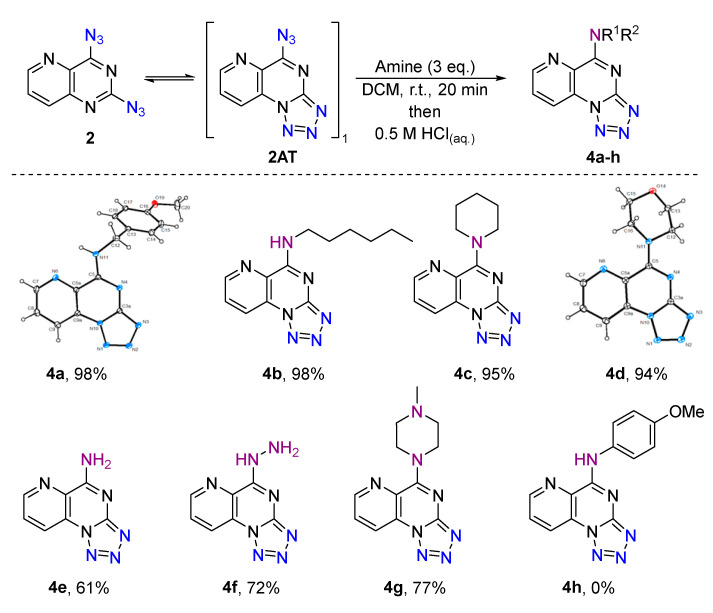
SnAr reaction of *diazide* **2** with amines.

Next, we explored the SnAr reaction between *diazide* **2** and the amines. As with the thiols, we adopted the previously used reaction conditions [28] and an addition of *p*-methoxybenzylamine to *diazide* **2** in DMSO provided product **4a** in 49% yield without an additional base. At this point, we decided to investigate the solvent effect on tautomerization and thus manipulate the site of nucleophile attack. To do this, we carried out SnAr reactions of *diazide* **2** with *p*-methoxybenzylamine in various solvents: toluene, benzene, DCM, EtOH, CHCl_3_, DMSO, and MeCN. In all cases, the same product **4a** was obtained. This means that 5-azidotetrazolo[1,5-*a*]pyrimidine tautomer **2AT** is always predominant, despite the selected solvents. The highest yield with the easiest work-up procedure was obtained in DCM, and it was the solvent of choice in further research. To explore the scope of the reaction, we used optimized conditions for the synthesis of different amino derivatives **4a**–**g** in good yields (Scheme 3). Products bearing the benzylic **4a**, aliphatic primary **4b**, and secondary **4c**, **4d**, **4g** amine moieties were obtained. In addition, ammonia and hydrazine showed good reactivity and provided products **4h** and **4f**. However, substitution of *diazide* **2** with aromatic amine (anisidine) to **4h** was unsuccessful and only the starting material was recovered after 3 days of stirring.

**Scheme 4:**
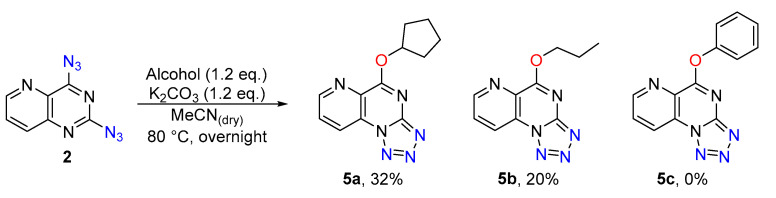
SnAr reaction of *diazide* **2** with alcohols.

Substitution of *diazide* **2** with simple alcohols proceeded in the presence of a base (K_2_CO_3_) in dry MeCN, yielding products **5a** and **5b** (Scheme 4), although the products were obtained in low yields, mainly due to partial hydrolysis in the basic reaction medium as a side reaction. A complex mixture of unidentified products was obtained in the reaction of *diazide* **2** with phenol, most probably due to further substitution reactions of the phenyloxy moiety as a leaving group.

**Scheme 5:**
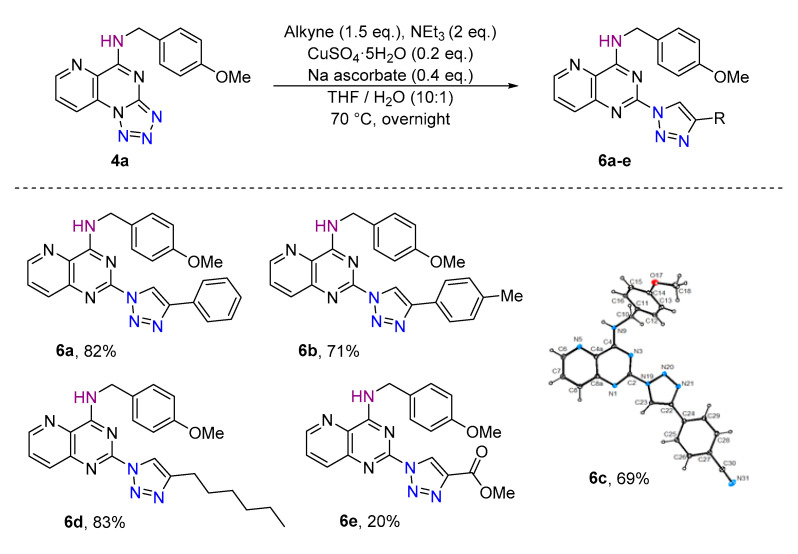
Synthesis of 2-triazolylpyrido[3,2-*d*]pyrimidines **6a–e**.

Given that the compounds **2**–**5** persist in equilibrium between tetrazolo[1,5-*a*]pyrimidines and 2-azidopyrimidines, it should be possible to functionalize them as hetarylazides [12,34] and tetrazoles [35]. Indeed, a series of 1,2,3-triazole-substituted pyrido[3,2-*d*]pyrimidines **6a**–**e** were obtained from tetrazolo[1,5-*a*]pyrido[2,3-*e*]pyrimidine **4a** in the copper(I)-catalyzed azide-alkyne cycloaddition (CuAAC) reaction (Scheme 5).

**Scheme 6:**
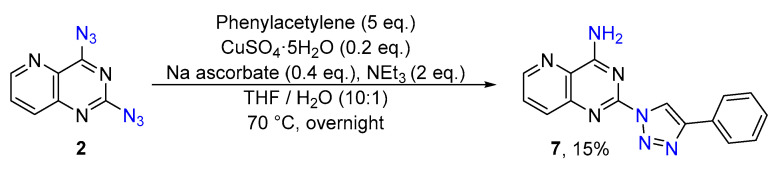
Partial reduction of compound **2** during its CuAAC reaction.

Synthesis of 2,4-bistriazole from *diazide* **2** was not successful due to the formation of multiple side products. The major component was found to be partially reduced 2-triazolylpyrido[3,2-*d*]pyrimidine **7** (Scheme 6). We [36,37,38] and others [39] have previously observed that azido groups can be selectively reduced to their respective amino derivatives by Cu(I), which is generated by the CuSO_4_/ascorbate system.

**Scheme 7:**
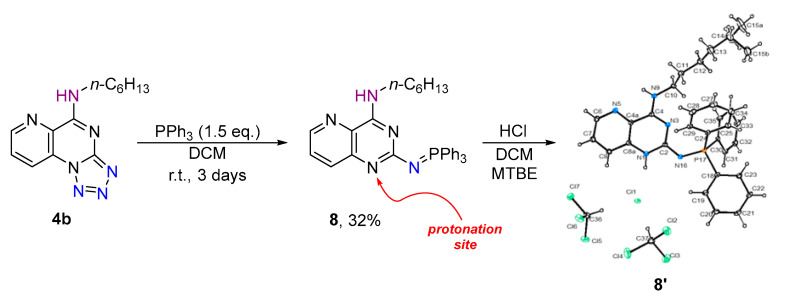
Synthesis of iminophosphorane **8** and its HCl salt **8′**.

Additionally, we were able to functionalize 5-aminotetrazolo[1,5-*a*]pyrimidine **4b** in the Staudinger reaction to iminophosphorane **8** (Scheme 7). Interestingly, its NMR and single crystal X-ray analysis revealed a protonated form **8′**, which was obtained by the precipitation of compound **8** with anhydrous HCl in the DCM/MTBE system. The protonation occurred at the N(1) position of the molecule.

**Scheme 8:**
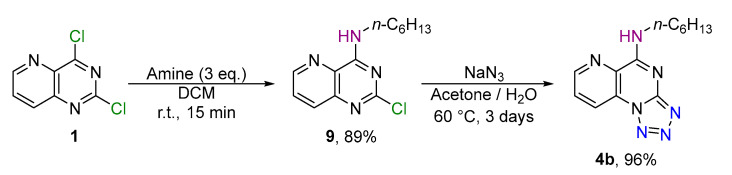
Conventional synthesis route for compound **4b**.

To confirm that the *C*-4 position is the more reactive site in pyrido[3,2-*d*]pyrimidines with identical leaving groups in positions *C*-2 and *C*-4 (substrate **1**), we switched the order of nucleophile addition. Indeed, the addition of amine first to the 2,4-dichloropyrido[3,2-*d*]pyrimidine (**1**), followed by sodium azide, afforded the expected **4b** (Scheme 8). However, it should be mentioned that the addition rate of the second nucleophile–azide was rather slow. It took 3 days to achieve near complete conversion of intermediate **9**. Previously, Boyomi et al. [29] reported failed attempts of 4-amino and 4-benzyloxy substituted 2-chloropyrido[3,2-*d*]pyrimidine substitution with sodium azide in refluxed ethanol. Moreover, in our case, the amino product **9** was obtained in high yield without the formation of the diamino product. The electron-donating effect of the amino group slowed or even inhibited further SnAr process.

**Scheme 9:**
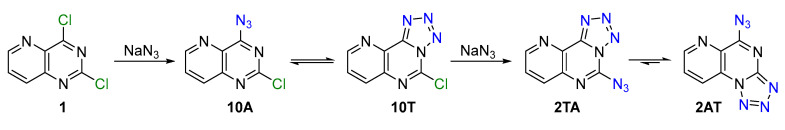
Synthesis pathway of *diazide* **2**.

It is interesting to note that the addition of two azido groups in the synthesis of *diazide* **2** was relatively fast (<1 h). This suggests that the first azido group after the addition to *C*-4 tautomerizes to tetrazole **10T**, where tetrazole, as an electron-withdrawing group, makes the pyrimidine system more reactive toward a second nucleophilic addition, and the final 2,4-disubstituted system is formed (Scheme 9).

### 2.2. Single Crystal X-ray Analysis

Compounds **2**, **3b**, **3f**, **4a**, **4d**, **5a**, and the product **8** protonated form **8′** were obtained in crystalline form and their chemical structures were confirmed by single crystal X-ray analysis. Crystal data and refinement details for the studied crystals are presented in Table 1. Search of the Cambridge structure database (CSD, version 5.43, November 2021) for synthesized pyrido[2,3-*e*]tetrazolo[1,5-*a*]pyrimidine heterosystem did not reveal any hits and, thus, gave evidence that it had not been studied by single crystal X-ray diffraction yet. Below, we discuss the geometry of this new tricyclic heterosystem in detail. The pyrido[3,2-*d*]pyrimidine heterosystem search gave five hits [40,41,42]. Comparison of compound **6c** with structures from CSD showed that their geometry fit very well.

In the crystal structure **2**, the tricyclic heterosystem was planar within ±0.021(1) Å. Atoms N12 and N13 of the azide group deviated from this plane by 0.0736(9) Å and 0.1356(11) Å, respectively. Thus, the azide group is involved in a common conjugate system of the molecule, and the C5-N11 single bond, equal to 1.391(2) Å, was shortened when compared to a standard single C–N bond [43]. The azide group was not exactly linear and the valence angle N11-N12-N13 was 171.88(12)°.

The crystal structure **3b** was a dichloromethane solvate. Heterocyclic fragment of the molecule was strictly planar. Deviation of the S11 atom from this plane was 0.121(1) Å. The lone electron pairs of S11 atom were involved in the common conjugate system of the heterosystem, which resulted in shortening of the bond C5-S11 = 1.731 (2) Å compared to a standard single C-S bond [43]. Aromatic fragments of the molecule, forming a dihedral angle of 5.15(9)°, were nearly parallel to each other.

In the crystal structure **3f**, some violation of the planarity of the heterocyclic system was observed. The dihedral angle between the tetrazolo-pyrimidine and pyridine fragments was 5.97(5)°. The C5-S11 bond (1.745(1) Å) in the **3f** structure was longer than in **3b**. The least squares mean planes of tricyclic heterosystem and cyclohexane fragments formed a dihedral angle of 61.40(7)°.

In the crystal structure **4a**, the heterocyclic system was strictly planar (±0.01 Å). Deviation of the N11 atom from this plane was 0.0358(11) Å and the lone electron pair of N11 atom participated in the common conjugate system of a tricycle. The dihedral angle between aromatic fragments of the molecule was 73.98(5)°. Orientation of the methoxy group was characterized by the torsion angle C20-O19-C16-C15 = −6.8(2)°.

In the crystal structure **4d**, we again observed minor violation of the planarity of the heterocyclic system. The dihedral angle between the tetrazolo-pyrimidine and pyridine fragments was 3.39(7)°. The least squares mean planes of the heterocyclic system and morpholine fragment formed a dihedral angle of 19.71(7)°. The morpholine fragment assumed a chair conformation. Atoms N11 and O14 deviated from the plane formed by four carbon atoms by 0.6142(15) Å and −0.6703(14) Å, respectively.

In the structure **6c**, the bicyclic heterosystem was sufficiently planar. The C4-N9 bond length was 1.3366 (15) Å. Dihedral angles of the triazole fragment with mean planes of bicycle and adjacent phenyl ring were 4.38(6)° and 11.16(7)°, respectively. The slope of the mean plane of the second phenyl fragment to the plane of the bicycle was 75.71(5)°. Orientation of the methoxy group was characterized by the torsion angle C18-O17-C14-C13 = 7.2(2)°.

Compound **8** was crystallized in the form of hydrochloride chloroform disolvate. In contrast to the previous structure **6a** in **8**, atom N1 became protonated and bond C2-N16 [1.328(2) Å] assumed a double bond character. Least squares planes of pyridine and pyrimidine fragments in the heterosystem formed a dihedral angle of 3.87(8)°. Two atoms at the end of an aliphatic chain in the structure were disordered and assumed two positions with an occupancy ratio of 0.7:0.3.

Since the pyrido[2,3-*e*]tetrazolo[1,5-*a*]pyrimidine heterosystem has not been studied by single crystal X-ray diffraction until now, we present a comparison of the geometric parameters with the tetrazolo[1,5-*a*]pyrimidine fragment of the crystal structures deposited with CCDC. Table 2 lists the selected geometrical parameters of the studied compounds and data from the literature.

The analysis of Table 2 shows that, overall, the geometry of the tetrazolo[1,5-*a*]pyrimidine fragment in the studied compounds corresponded to the published data. The geometry of the tetrazole fragment was the most conservative and was practically the same in all structures. The most variable bonds of the heterocyclic system were C3a-N4 and N4-C5. Their length was related to the type of substituent in position 5.

### 2.3. Free Energy Calculation for Azide-Tetrazole Equilibrium of Substituted Tetrazolo[1,5-a]pyrido[2,3-e] Pyrimidines

The system in equilibrium can be quantitatively characterized by thermodynamic values—Gibbs free energy, enthalpy, and entropy. The Gibbs free energy describes the equilibrium at given state of conditions, while the enthalpy defines the absolute stability of the tetrazole system (a higher value means a higher stability of tetrazole).

The Gibbs–Helmholtz equation ΔG = −RTln(K_eq_) was used to calculate the Gibbs free energy of tautomerization [50]. ^1^H NMR spectra of tetrazoles **3**–**5** were acquired in CDCl_3_ at variable temperatures (see Supplementary Materials) to obtain equilibrium constants (K_eq_) expressed as the integral ratio of tetrazole/azido tautomeric forms K_(eq)_ = [T]/[A]. Enthalpy and entropy values were obtained by plotting the Gibbs free energy equation ΔG = ΔH − TΔS (see Supplementary Materials). The calculated thermodynamic values for the tautomerization of the obtained compounds are given in Table 3. Very similar results were also obtained by plotting the van’t Hoff equation (see Supplementary Materials). Errors were calculated using the mean square error method.

As previously mentioned, the azide-tetrazole equilibrium is influenced by the solvent polarity, temperature, substituent electronic effects, and sterics [8]. In our case, the equilibrium of tetrazolo[1,5-*a*]pyrido[2,3-*e*]pyrimidines **3**–**5** was fully shifted toward tetrazole in DMSO-*d*_6_ and the azido tautomer was not observed in this solvent. On the other hand, the equilibrium in less polar CDCl_3_ was notable and varied with different substituents. As expected, the equilibrium shifted toward the azido tautomer at elevated temperatures. The calculated negative enthalpy values confirmed that the tetrazole is an energetically more stable form and the negative Gibbs free energy affirms that tetrazole in pyrido[3,2-*d*]pyrimidines **3**–**5** is a major tautomer present at 25 °C.

**Figure 2:**
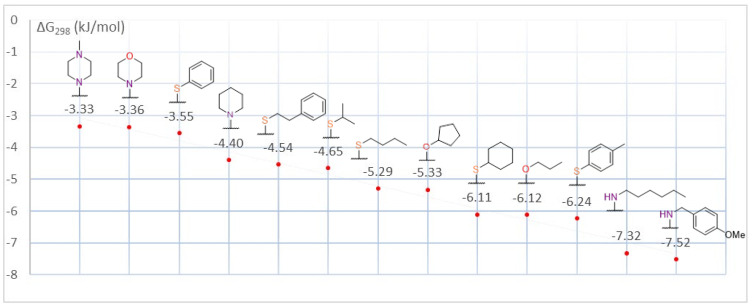
Composition of the Gibbs free energy of tautomerization for the substituted tetrazoles **3**–**5**.

It is well-known that electron donating substituents stabilize the fused tetrazole ring, while electron withdrawing substituents favor the azido tautomer. In our case, the Gibbs free energy values of *p*-methoxybenzylamino- (**4a**) and hexylamino- (**4b**) products were the highest. Therefore, the equilibrium was strongly shifted toward the tetrazole tautomer (Figure 2). However, the Gibbs free energy values for products containing secondary amine moieties piperidine (**4c**), morpholine (**4d**), and *N*-methylpiperazine (**4g**) were significantly lower than those of primary amine moieties **4a** and **4b**. Additionally, alkoxy-substituted **5a** and **5b** were shifted toward the tetrazole tautomer and the Gibbs free energy values were higher than those of the thiols.

### 2.4. Tautomerism of Diazidopyrido[3,2-d]Pyrimidine

**Figure 3:**
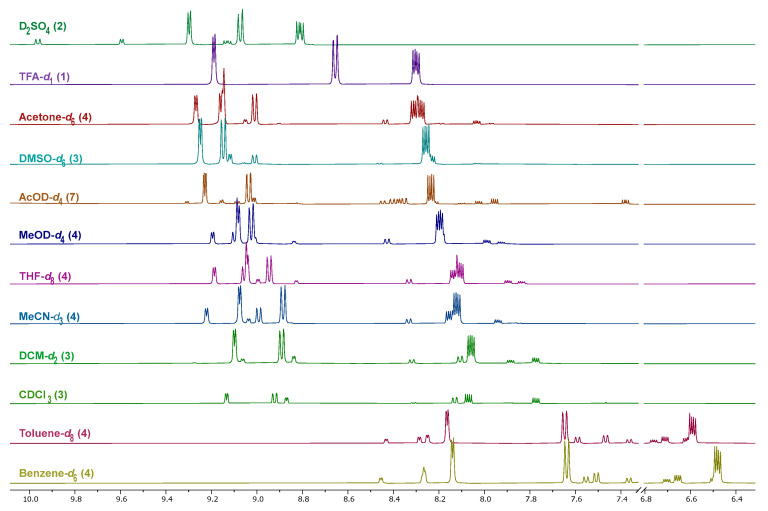
^1^H-NMR spectra of *diazide* **2** in various solvents (number of the observed tautomers reported in parentheses).

Finally, we looked at the tautomeric equilibrium of *diazide* **2**. The ^1^H-NMR spectra of *diazide* **2** in various solvents are shown in Figure 3. A different number of tautomeric forms were present depending on the solvent polarity. Tetrazole as the electron withdrawing moiety shifted signals downfield, while azido tautomer signals were more upfield. By going up in solvent polarity, the signals appeared more downfield and the ratio of the downfield/upfield signals increased. Thus, as in theory, the tetrazole tautomer becomes more dominant in polar solvents. At the present time, we are unable to undeniably provide the structural identity of each set of signals. For a thorough assignment of tautomeric forms, ^15^N labeling is required.

**Figure 4:**
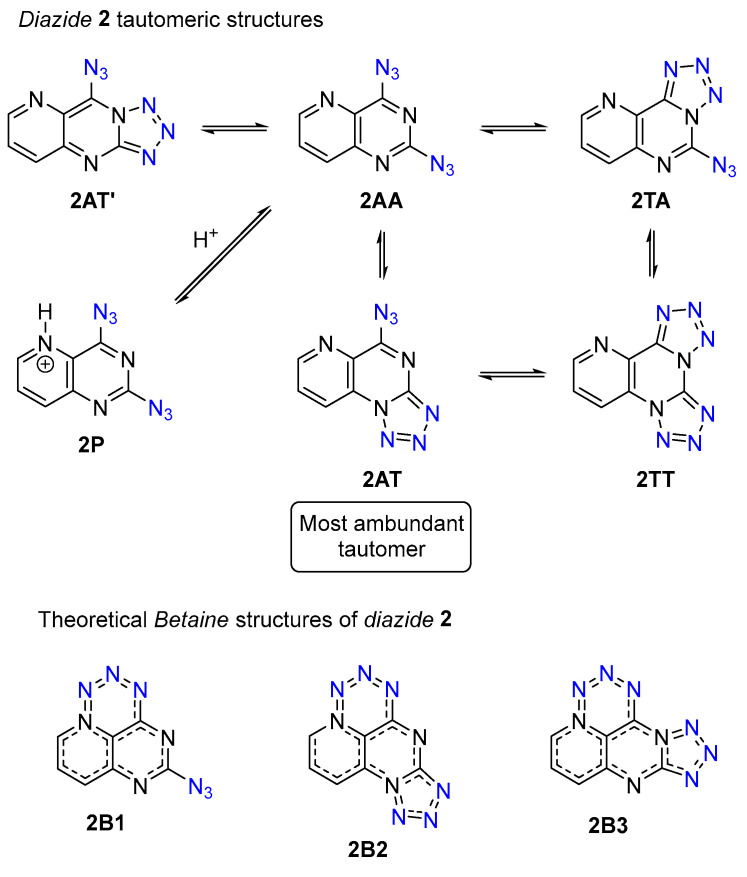
Tautomeric structures of *diazide* **2**.

**Figure 5:**
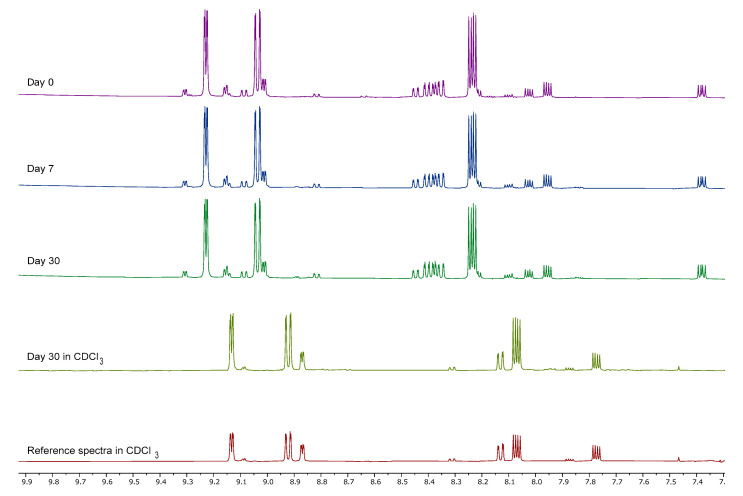
^1^H-NMR spectra of *diazide* **2** in AcOD-*d*_4_ and CDCl_3_.

In most cases, three to four tautomeric forms were observed. In TFA, only one tautomeric form was present and two tautomeric forms were observed in D_2_SO_4_. It is most likely that these solvents shift the equilibrium to bistetrazole **2TT** due to far-out polarity. However, the pyridine ring in such acidic conditions can be protonated, making the ring system extremely electron deficient and shifting the equilibrium toward diazide **2P**. It is interesting to note that in AcOD-*d*_4_, seven out of nine possible tautomeric forms were present. There are five possible tautomeric structures and three betaine structures for *diazide* **2** (Figure 4). To prove that these are indeed tautomeric forms, we acquired spectra after prolonged storage and redissolving the stored sample in a different solvent. To our delight, acquiring spectra after 7 days and 30 days of storage at 4 °C in acetic acid solution presented identical spectra to that of the freshly prepared sample (Figure 5). Furthermore, evaporation of the acetic acid and redissolving the 30 day stored sample in CDCl_3_ provided identical spectra to one obtained by dissolving *diazide* **2** in CDCl_3_.

## 3. Materials and Methods

### 3.1. General Information

Reagents purchased from Alfa Aesar, Acros Organics, Sigma Aldrich were used as received. All solvents were distilled prior to use. THF and toluene were distilled from Na under an Ar atmosphere. DMF and DMSO were distilled from CaH_2_ under reduced pressure. For column chromatography, ROCC silica gel (40–60 µm, 60 Å) was used. Chromatography was monitored by TLC (E. Merck Kieselgel 60 F_254_) and visualized with UV light.

HPLC analysis was performed using an Agilent Technologies 1200 Series system equipped with an X Bridge C-18 column, 4.6 × 150 mm, particle size 3.5 μm, with a flow rate of 1 mL/min, using 0.1% TFA/H_2_O and MeCN for the mobile phase.

The IR spectra were recorded in KBr with a Perkin-Elmer Spectrum BX FTIR spectrometer (4000−450 cm^−1^).

High-resolution mass (HRMS) (electrospray ionization (ESI)) was recorded with an Agilent 1290 Infinity series ultra-high pressure liquid chromatography connected to an Agilent 6230 time-of-flight mass spectrometer or (atmospheric pressure chemical ionization (APCI)) on a 7 T solariX XR (Bruker Daltonik GmbH) Fourier transform ion cyclotron resonance mass spectrometer equipped with an APCI source.

Single-crystal diffraction data were collected on an XtaLAB Synergy-S Dualflex diffractometer (Rigaku Corporation, Tokyo, Japan) equipped with a HyPix6000 detector and micro-focus sealed X-ray tube (Rigaku, Tokyo, Japan) using Cu Kα radiation (λ = 1.54184 Å). Single crystals were fixed with oil in a nylon loop of a magnetic CryoCap and set on a goniometer head. The samples were cooled down to 150 K, and ω-scans were performed with a step size of 0.5°. Data collection and reduction were performed with CrysAlisPro 1.171.40.35a software (Oxford Diffraction Ltd., Abingdon, UK). The structure solution and refinement were performed with SHELXT [50] and SHELXL [51] software, which are part of the CrysAlisPro and Olex2 suites. The H atoms were positioned geometrically and treated as riding on their parent C or N atoms. Molecular graphics were prepared using ORTEP3 for Windows [52] and Mercury [53]. The PLATON [54] tool was used for the geometrical calculations.

^1^H and ^13^C NMR spectra were recorded on a Bruker Avance 500 spectrometer (Bruker BioSpin GmbH, Rheinstetten, Germany). Chemical shifts (δ) were reported in ppm and coupling constants (*J*) in Hz. Residual solvent peaks (^1^H) or (^13^C) were used as the reference (for ^1^H-NMR: CDCl_3_ δ = 7.26 ppm, DMSO-*d*_6_ δ = 2.50 ppm, (CD_3_)_2_CO δ = 2.05 ppm, CD_3_COOD δ = 2.04 ppm, C_6_D_6_ δ = 7.16 ppm, CD_2_Cl_2_ δ = 5.32 ppm, D_2_SO_4_ δ = 11.20 ppm, CD_3_CN δ = 1.94 ppm, CD_3_OD δ = 3.31 ppm, CF_3_COOD δ = 11.50 ppm, THF-*d*_8_ δ = 3.58 ppm, toluene-*d*_8_ δ = 6.98 ppm and for ^13^C-NMR: CDCl_3_ δ = 77.16 ppm, DMSO-*d*_6_ δ = 39.52 ppm). H_3_PO_4_ (85% aq.) δ = 0.00 ppm was used as the external standard for ^31^P NMR. Multiplicities were reported as s (singlet), d (doublet), t (triplet), q (quartet), and m (multiplet).

### 3.2. Synthesis Methods and Product Characterization

General procedure A: A synthesis of 5-thiotetrazolo[1,5-*a*]pyrido[2,3-*e*]pyrimidines **3c**–**f**. Thiol (1.5 eq) was added to 2,4-diazidopyrido[3,2-*d*]pyrimidine (2) (1 eq) and triethylamine (1.2 eq) in DCM (1 mL) in a 10 mL glass vial and stirred for 15 min at ambient temperature. After the reaction completion (HPLC monitoring), the reaction mixture was filtered through a silica gel plug with 10% DCM/MeCN and evaporated under reduced pressure to yield the crude product.

General procedure B: A synthesis of 5-aminotetrazolo[1,5-*a*]pyrido[2,3-*e*]pyrimidines **4a**–**d**. An amine (3 eq) was added to 2,4-diazidopyrido[3,2-*d*]pyrimidine (2) (1 eq) in DCM (1 mL) in a 10 mL glass vial and stirred for 15 min at ambient temperature. After the reaction completion (HPLC monitoring), an additional DCM (5 mL) was added and the mixture was washed with 0.5 M HCl_(aq.)_ solution (2 × 5 mL), followed by saturated NaCl_(aq.)_ wash (2 × 5 mL). The organic phase was dried over anhydrous Na_2_SO_4_, then filtered and evaporated to yield the product.

General procedure C: A synthesis of triazoles **6a**–**e**. Sodium ascorbate (0.4 eq), CuSO_4_·5H_2_O (0.2 eq), NEt_3_ (2 eq), *N*-(4-methoxybenzyl)pyrido[2,3-*e*]tetrazolo[1,5-*a*]pyrimidin-5-amine (**4a**) (1 eq), and substituted acetylene (1.5 eq) were dissolved in THF (1 mL) and H_2_O (0.1 mL) in a 10 mL glass vial and stirred at 60 °C overnight. The resulting mixture was filtered through silica gel and the Na_2_SO_4_ plug, evaporated under reduced pressure. Crude product was purified by column chromatography.

*2,4-Diazidopyrido[3,2-d]pyrimidine* (**2**):

2,4-Dichloropyrido[3,2-*d*]pyrimidine (**1**) (1 g, 5 mmol, 1 eq) and NaN_3_ (1.6 g, 20 mmol, 4 eq) were weighed in a 50 mL round bottom flask. Acetone (10 mL) and water (1 mL) was added, and the mixture was stirred at 50 °C for 1 h. After the reaction completion (monitored by HPLC), the reaction mixture was cooled to room temperature and the solvent was evaporated under reduced pressure. Water (20 mL) was added to the mixture and extracted with dichloromethane (3 × 20 mL). Combined organic phases were washed with a saturated NaCl solution (2 × 10 mL) and dried over anhydrous Na_2_SO_4_, filtered, and evaporated under reduced pressure. Product was obtained as a slightly yellow amorphous solid (1 g, 95%). The product can be recrystallized from EtOH to obtain yellowish crystals (m.p. 168 °C). A single crystal for X-ray analysis was obtained by slow evaporation from DCM/MeOH.

^1^H NMR (500 MHz, CF_3_COOD) δ 9.19 (dd, 1H, ^3^*J* = 4.7 Hz, ^4^*J* = 1.4 Hz, H-C(8)), 8.66 (dd, 1H, ^3^*J* = 8.8 Hz, ^4^*J* = 1.4 Hz, H-C(6)), 8.30 (dd, 1H, ^3^*J* = 8.8, 4.7 Hz, H-C(7)) ppm. ^13^C NMR (125 MHz, CF_3_COOD) δ 169.0, 160.6, 152.0, 143.2, 136.0, 135.6, 127.7 ppm.

Observed as a mixture of three tautomers in a 80:17:3 ratio in DMSO-*d*_6_. First tautomer: ^1^H NMR (500 MHz, DMSO-*d*_6_) δ 9.25 (dd, 1H, ^3^*J* = 4.7 Hz, ^4^*J* = 1.5 Hz), 9.15 (dd, 1H, ^3^*J* = 8.6 Hz, ^4^*J* = 1.5 Hz), 8.26 (dd, 1H, ^3^*J* = 8.5, 4.5 Hz) ppm. Second tautomer: ^1^H NMR (500 MHz, DMSO-*d*_6_) δ 9.12 (dd, 1H, ^3^*J* = 4.5 Hz, ^4^*J* = 1.5 Hz), 9.01 (dd, 1H, ^3^*J* = 8.5 Hz, ^4^*J* = 1.5 Hz), 8.23 (dd, 1H, ^3^*J* = 8.5, 4.4 Hz) ppm. Third tautomer: ^1^H NMR (500 MHz, DMSO-*d*_6_) δ 9.06 (dd, 1H, ^3^*J* = 4.6 Hz, ^4^*J* = 1.6 Hz), 8.46 (dd, 1H, ^3^*J* = 8.4 Hz, ^4^*J* = 1.6 Hz), 8.03 (dd, 1H, ^3^*J* = 8.4, 4.3 Hz) ppm.

Observed as a mixture of three tautomers in a 59:35:6 ratio in CDCl_3_. First tautomer: ^1^H NMR (500 MHz, CDCl_3_) δ 9.13 (dd, 1H, ^3^*J* = 4.5 Hz, ^4^*J* = 1.5 Hz), 8.92 (dd, 1H, ^3^*J* = 8.5 Hz, ^4^*J* = 1.5 Hz), 8.07 (dd, 1H, ^3^*J* = 8.5, 4.5 Hz) ppm. Second tautomer: ^1^H NMR (500 MHz, CDCl_3_) δ 8.86 (dd, 1H, ^3^*J* = 4.3 Hz, ^4^*J* = 1.5 Hz), 8.12 (dd, 1H, ^3^*J* = 8.5 Hz, ^4^*J* = 1.5 Hz), 7.77 (dd, 1H, ^3^*J* = 8.5, 4.3 Hz) ppm. Third tautomer: ^1^H NMR (500 MHz, CDCl_3_) δ 9.08 (dd, 1H, ^3^*J* = 4.4 Hz, ^4^*J* = 1.5 Hz), 8.31 (dd, 1H, ^3^*J* = 8.5 Hz, ^4^*J* = 1.5 Hz), 7.87 (dd, 1H, ^3^*J* = 8.5, 4.4 Hz) ppm.

IR (KBr): 3074, 2377, 2225, 2164, 2144, 1598, 1539 cm^−1^.

HRMS calculated for [C_7_H_3_N_9_ + H^+^] = 214.0584, found 214.0584.

*5-(Butylthio)pyrido[2,3-e]tetrazolo[1,5-a]pyrimidine* (**3a**):

2,4-Diazidopyrido[3,2-*d*]pyrimidine (100 mg, 0.469 mmol, 1 eq) and K_2_CO_3_ (71 mg, 0.516 mmol, 1.1 eq) were added to a 10 mL round bottom flask, flushed with N_2_, and capped with a septum. Through the septum, absolute DMF (1 mL) and buthanethiol (44 mg, *d* = 0.84 g/mL, v = 52 µL, 0.493 mmol, 1.05 eq) were added and stirred for 1 h at room temperature. After the reaction completion (HPLC control), water (10 mL) was added and extracted with toluene (3 × 10 mL). The combined organic phase was washed with 5% LiCl solution (2 × 10 mL) and dried over anhydrous Na_2_SO_4_, filtered, and evaporated under reduced pressure to give the crude product. The crude product purification by silica gel column chromatography (DCM/MeOH, gradient 0→2%) and crystallization from *n*-PrOH yielded yellow crystals (57 mg, 47%, R_f_ = 0.50 in 50% Hex/EtOAc, m.p. 136 °C).

^1^H NMR (500 MHz, DMSO-*d*_6_) δ 9.06 (dd, 1H, ^3^*J* = 4.5 Hz, ^4^*J* = 1.4 Hz, H-C(7)), 8.94 (dd, 1H, ^3^*J* = 8.5 Hz, ^4^*J* = 1.4 Hz, H-C(9)), 8.18 (dd, 1H, ^3^*J* = 8.5, 4.5 Hz, H-C(8)), 3.35 (t, 2H, ^3^*J* = 7.4 Hz, H_2_-C(1′)), 1.78 (quintet, 2H, ^3^*J* = 7.4 Hz, H_2_-C(2′)), 1.51 (sextet, 2H, ^3^*J* = 7.4 Hz, H_2_-C(3′)), 0.96 (t, 3H, ^3^*J* = 7.4 Hz, H_3_-C(4′)) ppm. ^13^C NMR (125 MHz, DMSO-*d*_6_) δ 174.3, 152.3, 150.3, 134.2, 130.1, 128.2, 124.9, 30.0, 29.0, 21.6, 13.5 ppm.

IR (KBr): 3067, 2963, 2932, 1592, 1526, 1504 cm^−1^.

HRMS calculated for [C_11_H_12_N_6_S + H^+^] = 261.0917, found 261.0922.

*2-Azido-4-(butylthio)pyrido[3,2-d]pyrimidine* (**3aA**) *and 5-(butylthio)pyrido[2,3-e]tetrazolo[1,5-a]pyrimidine* (**3a**): observed in CDCl_3_ solution as a tautomer mixture in 11:89 ratio.

*Azide*: ^1^H NMR (500 MHz, CDCl_3_) δ 8.81 (dd, 1H, ^3^*J* = 4.5 Hz, ^4^*J* = 1.5 Hz, H-C(6)), 8.08 (dd, 1H, ^3^*J* = 8.5 Hz, ^4^*J* = 1.5 Hz, H-C(8)), 7.72 (dd, 1H, ^3^*J* = 8.5, 4.5 Hz, H-C(7)), 3.32 (t, 2H, ^3^*J* = 7.4 Hz, H_2_-C(1′)), 1.85 (quintet, 2H, ^3^*J* = 7.4 Hz, H_2_-C(2′)), 1.56 (sextet, 2H, ^3^*J* = 7.4 Hz, H_2_-C(3′)), 1.00 (t, 3H, ^3^*J* = 7.4 Hz, H_3_-C(4′)) ppm.

*Tetrazole*: ^1^H NMR (500 MHz, CDCl_3_) δ 9.06 (dd, 1H, ^3^*J* = 4.5 Hz, ^4^*J* = 1.5 Hz, H-C(7)), 8.85 (dd, 1H, ^3^*J* = 8.5 Hz, ^4^*J* = 1.5 Hz, H-C(9)), 7.99 (dd, 1H, ^3^*J* = 8.5, 4.5 Hz, H-C(8)), 3.46 (t, 2H, ^3^*J* = 7.4 Hz, H_2_-C(1′)), 1.85 (quintet, 2H, ^3^*J* = 7.4 Hz, H_2_-C(2′)), 1.56 (sextet, 2H, ^3^*J* = 7.4 Hz, H_2_-C(3′)), 1.00 (t, 3H, ^3^*J* = 7.4 Hz, H_3_-C(4′)) ppm.

*5-(Phenethylthio)pyrido[2,3-e]tetrazolo[1,5-a]pyrimidine* (**3b**):

2,4-Diazidopyrido[3,2-*d*]pyrimidine (100 mg, 0.469 mmol, 1 eq) and K_2_CO_3_ (71 mg, 0.516 mmol, 1.1 eq) were added to a 10 mL round bottom flask, flushed with N_2_, and capped with a septum. Through the septum, absolute DMF (1 mL) and phenethanethiol (68 mg, *d* = 1.03 g/mL, v = 66 µL, 0.493 mmol, 1.05 eq) were added and stirred for 1 h at room temperature. After the reaction completion (HPLC control), water (10 mL) was added and extracted with toluene (3 × 10 mL). The combined organic phase was washed with 5% LiCl solution (2 × 10 mL) and dried over anhydrous Na_2_SO_4_, filtered, and evaporated under reduced pressure to give the crude product. Crystallization from *n*-PrOH yielded yellow crystals (93 mg, 63%, m.p. 184 °C). A single crystal for X-ray analysis was obtained by slow evaporation from DCM/Hex.

^1^H NMR (500 MHz, DMSO-*d*_6_) δ 9.06 (dd, 1H, ^3^*J* = 4.4 Hz, ^4^*J* = 1.3 Hz, H-C(7)), 8.95 (dd, 1H, ^3^*J* = 8.5 Hz, ^4^*J* = 1.3 Hz, H-C(9)), 8.18 (dd, 1H, ^3^*J* = 8.5, 4.4 Hz, H-C(8)), 7.22–7.40 (m, 5H, 5 × H-C(Ar)), 3.61 (t, 2H, ^3^*J* = 7.5 Hz, H_2_-C(1′)), 3.11 (t, 2H, ^3^*J* = 7.5 Hz, H_2_-C(2′)) ppm. ^13^C NMR (125 MHz, DMSO-*d*_6_) δ 174.1, 152.3, 150.3, 139.9, 134.2, 130.2, 128.6, 128.5, 128.2, 126.5, 124.9, 33.6, 30.8 ppm.

IR (KBr): 3078, 1596, 1533, 1504 cm^−1^.

HRMS calculated for [C_15_H_12_N_6_S + H^+^] = 309.0917, found 309.0899.

*2-Azido-4-(phenethylthio)pyrido[3,2-d]pyrimidine* (**3bA**) *and 5-(phenethylthio)pyrido[2,3-e]tetrazolo[1,5-a]pyrimidine* (**3b**): observed in CDCl_3_ solution as a tautomer mixture in 7:43 ratio.

*Azide*: ^1^H NMR (500 MHz, CDCl_3_) δ 8.81 (d, 1H, ^3^*J* = 4.2 Hz, H-C(6)), 8.09 (d, 1H, ^3^*J* = 8.6 Hz, H-C(8)), 7.72 (dd, 1H, ^3^*J* = 8.6, 4.2 Hz, H-C(7)), 7.22-7.37 (m, 5H, 5 × H-C(Ar)), 3.59 (t, 2H, ^3^*J* = 7.7 Hz, H-C(1′)), 3.12 (t, 2H, ^3^*J* = 7.7 Hz, H-C(2′)) ppm.

*Tetrazole*: ^1^H NMR (500 MHz, CDCl_3_) δ 9.04 (d, 1H, ^3^*J* = 4.5 Hz, H-C(7)), 8.86 (d, 1H, ^3^*J* = 8.5 Hz, H-C(9)), 7.99 (dd, 1H, ^3^*J* = 8.5, 4.5 Hz, H-C(8)), 7.22–7.37 (m, 5H, 5 × H-C(Ar)), 3.71 (t, 2H, ^3^*J* = 7.6 Hz, H-C(1′)), 3.17 (t, 2H, ^3^*J* = 7.6 Hz, H-C(2′)) ppm.

*5-(p-Tolylthio)pyrido[2,3-e]tetrazolo[1,5-a]pyrimidine* (**3c**):

Prepared according to procedure A using 2,4-diazidopyrido[3,2-*d*]pyrimidine (**2**) (50 mg, 0.235 mmol, 1 eq), *p*-thiocresol (35 mg, 0.282 mmol, 1.2 eq), and triethylamine (36 mg, *d* = 0.73 g/mL, v = 49 µL, 0.353 mmol, 1.5 eq). Crystallization from *n*-PrOH yielded yellow crystals (48 mg, 69%, m.p. 235 °C).

^1^H NMR (500 MHz, DMSO-*d*_6_) δ 9.14 (dd, 1H, ^3^*J* = 4.4 Hz, ^4^*J* = 1.5 Hz, H-C(7)), 8.98 (dd, 1H, ^3^*J* = 8.5 Hz, ^4^*J* = 1.5 Hz, H-C(9)), 8.23 (dd, 1H, ^3^*J* = 8.5, 4.4 Hz, H-C(8)), 7.57 (d, 2H, ^3^*J* = 8.0 Hz, 2 × H-C(1′)), 7.41 (d, 2H, ^3^*J* = 8.0 Hz, 2 × H-C(2′)), 2.44 (s, 3H, H_3_-C(3′)) ppm. ^13^C NMR (125 MHz, DMSO-*d*_6_) δ 174.1, 152.3, 150.4, 140.2, 135.5, 133.7, 130.4 (2 × C), 128.4, 125.0, 122.9, 21.0 ppm.

IR (KBr): 3082, 2919, 1592, 1540, 1504 cm^−1^.

HRMS calculated for [C_14_H_10_N_6_S + H^+^] = 295.0760, found 295.0787.

*2-Azido-4-(p-tolylthio)pyrido[3,2-d]pyrimidine* (**3cA**) *and 5-(p-tolylthio)pyrido[2,3-e]tetrazolo[1,5-a]pyrimidine* (**3c***):* observed in CDCl_3_ solution as a tautomer mixture in 1:9 ratio.

*Azide*: ^1^H NMR (500 MHz, CDCl_3_) δ 8.88 (dd, 1H, ^3^*J* = 4.2 Hz, ^4^*J* = 1.5 Hz, H-C(6)), 8.10 (dd, 1H, ^3^*J* = 8.5 Hz, ^4^*J* = 1.5 Hz, H-C(8)), 7.75 (dd, 1H, ^3^*J* = 8.5, 4.2 Hz, H-C(7)), 7.52 (d, 2H, ^3^*J* = 8.0 Hz, 2 × H-C(1‘)), 7.30 (d, 2H, ^3^*J* = 8.0 Hz, 2 × H-C(2′)), 2.43 (s, 3H, H_3_-C(3′)) ppm.

*Tetrazole*: ^1^H NMR (500 MHz, CDCl_3_) δ 9.11 (dd, 1H, ^3^*J* = 4.5 Hz, ^4^*J* = 1.5 Hz, H-C(7)), 8.87 (dd, 1H, ^3^*J* = 8.5 Hz, ^4^*J* = 1.5 Hz, H-C(9)), 8.03 (dd, 1H, ^3^*J* = 8.5, 4.5 Hz, H-C(8)), 7.53 (d, 2H, ^3^*J* = 8.0 Hz, 2 × H-C(1′)), 7.33 (d, 2H, ^3^*J* = 8.0 Hz, 2 × H-C(2′)), 2.46 (s, 3H, H_3_-C(3′)) ppm.

*5-(Isopropylthio)pyrido[2,3-e]tetrazolo[1,5-a]pyrimidine* (**3d**)*:*

Prepared according to procedure A using 2,4-diazidopyrido[3,2-*d*]pyrimidine (**2**) (50 mg, 0.235 mmol, 1 eq), isopropanethiol (22 mg, *d* = 0.82 g/mL, v = 26 µL, 0.282 mmol, 1.2 eq), and triethylamine (36 mg, *d* = 0.73 g/mL, v = 49 µL, 0.353 mmol, 1.5 eq). Yielded a yellow amorphous solid (55 mg, 95%). Crystallization from *n*-PrOH yielded yellowish crystals (m.p. 151 °C).

^1^H NMR (500 MHz, DMSO-*d*_6_) δ 9.05 (dd, 1H, ^3^*J* = 4.5 Hz, ^4^*J* = 1.4 Hz, H-C(7)), 8.94 (dd, 1H, ^3^*J* = 8.5 Hz, ^4^*J* = 1.4 Hz, H-C(9)), 8.17 (dd, 1H, ^3^*J* = 8.5, 4.5 Hz, H-C(8)), 4.18 (heptet, 1H, ^3^*J* = 6.9 Hz, H-C(1′)), 1.50 (d, 6H, ^3^*J* = 6.9 Hz, H-C(2′)) ppm. ^13^C NMR (125 MHz, DMSO-*d*_6_) δ 174.0, 152.3, 150.2, 134.1, 130.1, 128.3, 124.9, 34.8, 22.2 ppm.

IR (KBr): 3063, 2968, 2952, 2926, 2865, 1598, 1527, 1505 cm^−1^.

HRMS calculated for [C_10_H_10_N_6_S + H^+^] = 247.0760, found 247.0769.

*2-Azido-4-(isopropylthio)pyrido[3,2-d]pyrimidine (***3dA***) and 5-(isopropylthio)pyrido[2,3-e]tetrazolo[1,5-a]pyrimidine* (**3d**)*:* observed in CDCl_3_ solution as a tautomer mixture in 13:87 ratio.

*Azide*: ^1^H NMR (500 MHz, CDCl_3_) δ 8.80 (dd, 1H, ^3^*J* = 4.3 Hz, ^4^*J* = 1.6 Hz, H-C(6)), 8.08 (dd, 1H, ^3^*J* = 8.5 Hz, ^4^*J* = 1.6 Hz, H-C(8)), 7.71 (dd, 1H, ^3^*J* = 8.5, 4.3 Hz, H-C(7)), 4.20 (heptet, 1H, ^3^*J* = 6.9 Hz, H-C(1′)), 1.53 (d, 6H, ^3^*J* = 6.9 Hz, H-C(2′)) ppm.

*Tetrazole*: ^1^H NMR (500 MHz, CDCl_3_) δ 9.04 (dd, 1H, ^3^*J* = 4.6 Hz, ^4^*J* = 1.5 Hz, H-C(7)), 8.85 (dd, 1H, ^3^*J* = 8.5 Hz, ^4^*J* = 1.5 Hz, H-C(9)), 7.98 (dd, 1H, ^3^*J* = 8.5, 4.6 Hz, H-C(8)), 4.38 (heptet, 1H, ^3^*J* = 6.8 Hz, H-C(1′)), 1.57 (d, 6H, ^3^*J* = 6.8 Hz, H-C(2′)) ppm.

*5-(Phenylthio)pyrido[2,3-e]tetrazolo[1,5-a]pyrimidine* (**3e**)*:*

Prepared according to procedure A using 2,4-diazidopyrido[3,2-*d*]pyrimidine (**2**) (50 mg, 0.235 mmol, 1 eq), thiophenol (31 mg, 0.282 mmol, 1.2 eq) and triethylamine (36 mg, *d* = 0.73 g/mL, v = 49 µL, 0.353 mmol, 1.5 eq). Purification by silica gel column chromatography (DCM without gradient) yielded a yellowish amorphous solid (54 mg, 82%, Rf = 0.80 in 50% Hex/EtOAc).

^1^H NMR (500 MHz, DMSO-*d*_6_) δ 9.15 (dd, 1H, ^3^*J* = 4.4 Hz, ^4^*J* = 1.4 Hz, H-C(7)), 8.99 (dd, 1H, ^3^*J* = 8.5 Hz, ^4^*J* = 1.4 Hz, H-C(9)), 8.24 (dd, 1H, ^3^*J* = 8.5, 4.4 Hz, H-C(8)), 7.58–7.72 (m, 5H, 5 × H-C(Ar)) ppm. ^13^C NMR (125 MHz, DMSO-*d*_6_) δ 173.8, 152.3, 150.5, 135.6, 133.7, 130.5, 130.3, 129.7, 128.4, 126.5, 125.0 ppm.

IR (KBr): 3085, 3064, 1592, 1542, 1533, 1506 cm^−1^.

HRMS calculated for [C_13_H_8_N_6_S + H^+^] = 281.0604, found 281.0630.

*2-Azido-4-(phenylthio)pyrido[3,2-d]pyrimidine* (**3eA**) *and 5-(phenylthio)pyrido[2,3-e]tetrazolo[1,5-a]pyrimidine* (**3e**)*:* observed in CDCl_3_ solution as a tautomer mixture in 19:81 ratio.

*Azide*: ^1^H NMR (500 MHz, CDCl_3_) δ 8.87 (dd, 1H, ^3^*J* = 4.3 Hz, ^4^*J* = 1.6 Hz, H-C(6)), 8.11 (dd, 1H, ^3^*J* = 8.5 Hz, ^4^*J* = 1.6 Hz, H-C(8)), 7.76 (dd, 1H, ^3^*J* = 8.5, 4.3 Hz, H-C(7)), 7.45–7.70 (m, 5H, 5 × H-C(Ar)) ppm.

*Tetrazole*: ^1^H NMR (500 MHz, CDCl_3_) δ 9.12 (dd, 1H, ^3^*J* = 4.4 Hz, ^4^*J* = 1.5 Hz, H-C(7)), 8.88 (dd, 1H, ^3^*J* = 8.5 Hz, ^4^*J* = 1.6 Hz, H-C(9)), 8.04 (dd, 1H, ^3^*J* = 8.5, 4.4 Hz, H-C(8)), 7.45–7.70 (m, 5H, 5 × H-C(Ar)) ppm.

*5-(Cyclohexylthio)pyrido[2,3-e]tetrazolo[1,5-a]pyrimidine* (**3f**)*:*

Prepared according to procedure A using 2,4-diazidopyrido[3,2-*d*]pyrimidine (**2**) (50 mg, 0.235 mmol, 1 eq), cyclohexanethiol (33 mg, *d* = 0.95 g/mL, v = 35 µL, 0.282 mmol, 1.2 eq), and triethylamine (36 mg, *d* = 0.73 g/mL, v = 49 µL, 0.353 mmol, 1.5 eq). Yellow amorphous solid (56 mg, 84%). Crystallization from *n*-PrOH yielded yellowish crystals (m.p. 156 °C). A single crystal for X-ray analysis was obtained by slow evaporation from DCM/Hex.

^1^H NMR (500 MHz, DMSO-*d*_6_) δ 9.04 (dd, 1H, ^3^*J* = 4.5 Hz, ^4^*J* = 1.2 Hz, H-C(7)), 8.93 (dd, 1H, ^3^*J* = 8.5 Hz, ^4^*J* = 1.2 Hz, H-C(9)), 8.17 (dd, 1H, ^3^*J* = 8.5, 4.5 Hz, H-C(8)), 4.05–4.12 (m, 1H, H-C(1′)), 2.11–2.19 (m, 2H, 2 × H-CH), 1.73–1.82 (m, 2H, 2 × H-CH), 1.32–1.68 (m, 6H, 6 × H-CH) ppm. ^13^C NMR (125 MHz, DMSO-*d*_6_) δ 173.7, 152.3, 150.2, 134.1, 130.2, 128.3, 124.9, 42.1, 31.8, 25.4, 25.2 ppm.

HRMS calculated for [C_13_H_14_N_6_S + H^+^] = 287.1073, found 287.1074.

*5-(Cyclohexylthio)pyrido[2,3-e]tetrazolo[1,5-a]pyrimidine* (**3f**) *and 2-azido-4-(cyclohexylthio)pyrido[3,2-d]pyrimidine* (**3fA**)*:* observed in CDCl_3_ solution as a tautomer mixture in 2:23 ratio.

*Azide*: ^1^H NMR (500 MHz, CDCl_3_) δ 8.80 (dd, 1H, ^3^*J* = 4.2 Hz, ^4^*J* = 1.5 Hz, H-C(6)), 8.08 (dd, 1H, ^3^*J* = 8.5 Hz, ^4^*J* = 1.5 Hz, H-C(8)), 7.70 (dd, 1H, ^3^*J* = 8.5, 4.2 Hz, H-C(7)), 4.05–4.12 (m, 1H, H-C(1′)), 1.34–2.26 (m, 10H, 5 × H_2_-C(c-Hex)) ppm.

*Tetrazole*: ^1^H NMR (500 MHz, CDCl_3_) δ 9.04 (dd, 1H, ^3^*J* = 4.6 Hz, ^4^*J* = 1.5 Hz, H-C(7)), 8.84 (dd, 1H, ^3^*J* = 8.5 Hz, ^4^*J* = 1.5 Hz, H-C(9)), 7.98 (dd, 1H, ^3^*J* = 8.5, 4.6 Hz, H-C(8)), 4.29 (tt, 1H, ^3^*J* = 10.0, 3.9 Hz, H-C(1′)), 1.34–2.26 (m, 10H, 5 × H_2_-C(c-Hex)) ppm.

*N-(4-Methoxybenzyl)pyrido[2,3-e]tetrazolo[1,5-a]pyrimidin-5-amine* (**4a**)*:*

Prepared according to procedure B using 2,4-diazidopyrido[3,2-*d*]pyrimidine (**2**) (50 mg, 0.235 mmol, 1 eq) and *p*-methoxybenzylamine (97 mg, *d* = 1.05 g/mL, v = 92 µL, 0.705 mmol, 1 eq). White amorphous solid (71 mg, 98%). Further crystallization from *n*-PrOH yielded white crystals (m.p. 200 °C). A single crystal for X-ray analysis was obtained by slow evaporation from DCM/Hex.

^1^H NMR (500 MHz, DMSO-*d*_6_) δ 9.58 (t, 1H, ^3^*J* = 6.4 Hz, H-N), 8.98 (dd, 1H, ^3^*J* = 4.5 Hz, ^4^*J* = 1.4 Hz (H-C(7)), 8.78 (dd, 1H, ^3^*J* = 8.4 Hz, ^4^*J* = 1.4 Hz, H-C(9)), 8.07 (dd, 1H, ^3^*J* = 8.4, 4.5 Hz, H-C(8)), 7.38 (d, 2H, ^3^*J* = 8.6 Hz, 2 × H-C(2′)), 6.88 (d, 2H, ^3^*J* = 8.6 Hz, 2 × H-C(3′)), 4.74 (d, 2H, ^3^*J* = 6.4 Hz, H_2_-C(1′)), 3.71 (s, 3H, H_3_-C(4′)) ppm. ^13^C NMR (125 MHz, DMSO-*d*_6_) δ 158.3, 157.0, 153.8, 149.2, 130.4, 129.3, 129.1 (2 × C), 129.0, 124.6, 113.7, 55.0, 43.2 ppm.

IR (KBr): 3235, 3080, 3004, 2930, 2832, 1615, 1538, 1517 cm^−1^.

HRMS calculated for [C_15_H_13_N_7_ + H^+^] = 308.1254, found 308.1224.

*2-Azido-N-(4-methoxybenzyl)pyrido[3,2-d]pyrimidin-4-amine (**4aA**) and N-(4-methoxybenzyl)pyrido[2,3-e]tetrazolo[1,5-a]pyrimidin-5-amine (**4a**):* observed in CDCl_3_ solution as a tautomer mixture in 1:19 ratio.

*Azide*: ^1^H NMR (500 MHz, CDCl_3_) δ 8.54 (dd, 1H, ^3^*J* = 4.3 Hz, ^4^*J* = 1.5 Hz, H-C(6)), 7.94 (dd, 1H, ^3^*J* = 8.4 Hz, ^4^*J* = 1.5 Hz, H-C(8)), 7.77 (bs, 1H, H-N), 7.58 (dd, 1H, ^3^*J* = 8.4, 4.3 Hz, H-C(7)), 7.34 (d, 2H, ^3^*J* = 8.6 Hz, 2 × H-C(2′)), 6.91 (d, 2H, ^3^*J* = 8.6 Hz, 2 × H-C(3′)), 4.76 (d, 2H, ^3^*J* = 5.8 Hz, H_2_-C(1′)), 3.8 (s, 3H, H_3_-C(4′)) ppm.

*Tetrazole*: ^1^H NMR (500 MHz, CDCl_3_) δ 8.84 (dd, 1H, ^3^*J* = 4.5 Hz, ^4^*J* = 1.4 Hz, H-C(7)), 8.73 (dd, 1H, ^3^*J* = 8.4 Hz, ^4^*J* = 1.4 Hz, H-C(9)), 7.88 (dd, 1H, ^3^*J* = 8.4, 4.5 Hz, H-C(8)), 7.77 (bs, 1H, H-N), 7.39 (d, 2H, ^3^*J* = 8.6 Hz, 2 × H-C(2′)), 6.91 (d, 2H, ^3^*J* = 8.6 Hz, 2 × H-C(3′)), 4.89 (d, 2H, ^3^*J* = 5.8 Hz, H_2_-C(1′)), 3.81 (s, 3H, H_3_-C(4′)) ppm.

*N-Hexylpyrido[2,3-e]tetrazolo[1,5-a]pyrimidin-5-amine* (**4b**)*:*

Prepared according to procedure B using 2,4-diazidopyrido[3,2-*d*]pyrimidine (**2**) (50 mg, 0.235 mmol, 1 eq) and hexylamine (71 mg, *d* = 0.77 g/mL, v = 92 µL, 0.705 mmol, 1 eq). White amorphous solid (64 mg, 98%). Further crystallization from *n*-PrOH yielded white crystals (m.p. 132 °C).

Alternative preparation from **9**: 2-Chloro-*N*-hexylpyrido[3,2-*d*]pyrimidin-4-amine (**9**) (50 mg, 0.189 mmol, 1 eq) and NaN_3_ (25 mg, 0.378 mmol, 2 eq) were weighed in a 10 mL vial. Acetone (1 mL) and water (0.1 mL) was added, and the mixture was stirred at 60 °C for 3 days. After the reaction completion (monitored by HPLC), the reaction mixture was cooled to room temperature and the solvent was evaporated under reduced pressure. Water (10 mL) was added to the mixture and extracted with dichloromethane (3 × 10 mL). Combined organic phases were washed with saturated NaCl solution (2 × 5 mL) and dried over anhydrous Na_2_SO_4_, filtered, and evaporated under reduced pressure. Product was obtained as a white amorphous solid (49 mg, 96%).

^1^H NMR (500 MHz, DMSO-*d*_6_) δ 9.12 (t, 1H, ^3^*J* = 6.7 Hz, H-N), 8.96 (d, 1H, ^3^*J* = 4.3 Hz, H-C(7)), 8.76 (d, 1H, ^3^*J* = 8.4 Hz, H-C(9)), 8.05 (dd, 1H, ^3^*J* = 8.4, 4.3 Hz, H-C(8)), 3.59 (q, 2H, ^3^*J* = 6.7 Hz, H_2_-C(1′)), 1.69 (quintet, 2H, ^3^*J* = 6.7 Hz, H_2_-C(2′)), 1.24–1.42 (m, 6H, H_2_-C(3′), H_2_-C(4′), H_2_-C(5′)), 0.82–0.92 (m, 3H, H_3_-C(6′)) ppm. ^13^C NMR (125 MHz, DMSO-*d*_6_) δ 157.0, 153.9, 149.1, 129.2, 129.1, 129.0, 124.6, 40.6, 31.0, 28.1, 26.2, 22.1, 13.9 ppm.

IR (KBr): 3388, 3068, 2967, 2936, 2854, 1613, 1576, 1539, 1525 cm^−1^.

HRMS calculated for [C_13_H_17_N_7_ + H^+^] = 272.1624, found 272.1635.

*2-Azido-N-hexylpyrido[3,2-d]pyrimidin-4-amine* (**4bA**) *and N-hexylpyrido[2,3-e]tetrazolo[1,5-a]pyrimidin-5-amine* (**4b**)*:* observed in CDCl_3_ solution as a tautomer mixture in 1:19 ratio.

*Azide:*^1^H NMR (500 MHz, CDCl_3_) δ 8.56 (d, 1H, ^3^*J* = 4.6 Hz, H-C(6)), 7.92 (d, 1H, ^3^*J* = 8.5 Hz, H-C(8)), 7.53–7.63 (m, 2H, H-N, H-C(7)), 3.61–3.67 (m, 2H, H_2_-C(1′)), 1.70–1.82 (m, 2H, H_2_-C(2′)), 1.28–1.50 (m, 6H, H_2_-C(3′), H_2_-C(4′), H_2_-C(5′)), 0.85-0.96 (m, 3H, H_3_-C(6′)) ppm.

*Tetrazole*: ^1^H NMR (500 MHz, CDCl_3_) δ 8.87 (d, 1H, ^3^*J* = 4.6 Hz, H-C(7)), 8.71 (d, 1H, ^3^*J* = 8.4 Hz, H-C(9)), 7.92 (dd, 1H, ^3^*J* = 8.4, 4.6 Hz, H-C(8)), 7.59 (bs, 1H, H-N), 3.77 (td, 2H, ^3^*J* = 7.5 Hz, 5.4 Hz, H_2_-C(1′)), 1.78 (quintet, 2H, ^3^*J* = 7.5 Hz, H_2_-C(2‘)) 1.28–1.51 (m, 6H, H_2_-C(3′), H_2_-C(4′), H_2_-C(5′)), 0.90 (t, 3H, ^3^*J* = 7.0 Hz, H-C(6′)) ppm.

*5-(Piperidin-1-yl)pyrido[2,3-e]tetrazolo[1,5-a]pyrimidine* (**4c**)*:*

Prepared according to procedure B using 2,4-diazidopyrido[3,2-*d*]pyrimidine (**2**) (50 mg, 0.235 mmol, 1 eq) and piperidine (60 mg, *d* = 0.86 g/mL, v = 70 µL, 0.705 mmol, 1 eq). White amorphous solid (58 mg, 95%). Further crystallization from *n*-PrOH yielded white crystals (m.p. 182 °C).

^1^H NMR (500 MHz, DMSO-*d*_6_) δ 8.98 (dd, 1H, ^3^*J* = 4.4 Hz, ^4^*J* = 1.6 Hz, H-C(7)), 8.79 (dd, 1H, ^3^*J* = 8.5 Hz, ^4^*J* = 1.6 Hz, H-C(9)), 8.03 (dd, 1H, ^3^*J* = 8.5, 4.4 Hz, H-C(8)), 4.10–4.60 (m, 4H, 4 × H-CH), 1.65–1.75 (m, 6H, 6 × H-CH) ppm. ^13^C NMR (125 MHz, DMSO-*d*_6_) δ 157.2, 152.8, 147.9, 130.9, 130.6, 128.3, 124.7, 49.2, 26.0, 24.1 ppm.

IR (KBr): 3049, 2938, 2849, 1605, 1587, 1538 cm^−1^.

HRMS calculated for [C_12_H_13_N_7_ + H^+^] = 256.1305, found 256.1314.

*2-Azido-4-(piperidin-1-yl)pyrido[3,2-d]pyrimidine* (**4cA**) *and 5-(piperidin-1-yl)pyrido[2,3-e]tetrazolo[1,5-a]pyrimidine* (**4c**): observed in CDCl_3_ solution as a tautomer mixture in 7:43 ratio.

*Azide*: ^1^H NMR (500 MHz, CDCl_3_) δ 8.58 (dd, 1H, ^3^*J* = 4.1 Hz, ^4^*J* = 1.6 Hz, H-C(6)), 7.92 (dd, 1H, ^3^*J* = 8.5 Hz, ^4^*J* = 1.6 Hz, H-C(8)), 7.51 (dd, 1H, ^3^*J* = 8.5, 4.1 Hz, H-C(7)), 4.20–4.80 (m, 4H, 4 × H-CH) 1.74–1.83 (m, 6H, 6 × H-CH) ppm.

*Tetrazole*: ^1^H NMR (500 MHz, CDCl_3_) δ 8.91 (dd, 1H, ^3^*J* = 4.4 Hz, ^4^*J* = 1.5 Hz, H-C(7)), 8.75 (dd, 1H, ^3^*J* = 8.4 Hz, ^4^*J* = 1.5 Hz, H-C(9)), 7.82 (dd, 1H, ^3^*J* = 8.4, 4.4 Hz, H-C(8)), 4.20–4.80 (m, 4H, 4 × H-CH) 1.74–1.83 (m, 6H, 6 × H-CH) ppm.

*4-(Pyrido[2,3-e]tetrazolo[1,5-a]pyrimidin-5-yl)morpholine* (**4d**)*:*

Prepared according to procedure B using *diazide* **2** (50 mg, 0.235 mmol, 1 eq) and morpholine (61 mg, *d* = 1.01 g/mL, v = 61 µL, 0.705 mmol, 1 eq). White amorphous solid (57 mg, 94%). Further crystallization from *n*-PrOH yielded white crystals (m.p. 215 °C). A single crystal for X-ray analysis was obtained by slow evaporation from DCM/Hex.

^1^H NMR (500 MHz, DMSO-*d*_6_) δ 8.98 (dd, 1H, ^3^*J* = 4.4 Hz, ^4^*J* = 1.4 Hz, H-C(7)), 8.83 (dd, 1H, ^3^*J* = 8.5 Hz, ^4^*J* = 1.4 Hz, H-C(9)), 8.06 (dd, 1H, ^3^*J* = 8.5, 4.4 Hz, H-C(8)), 4.18–4.63 (m, 4H, 4 × H-C(1′)), 3.78–3.84 (m, 4H, 4 × H-C(2′)) ppm. ^13^C NMR (125 MHz, DMSO-*d*_6_) δ 157.4, 152.6, 148.0, 130.9, 130.6, 128.5, 124.8, 66.2, 48.6 ppm.

IR (KBr): 3045, 3007, 2904, 2858, 1604, 1589, 1550, 1523 cm^−1^.

HRMS calculated for [C_11_H_11_N_7_O + H^+^] = 258.1098, found 258.1099.

*4-(2-Azidopyrido[3,2-d]pyrimidin-4-yl)morpholine* (**4dA***) and 4-(pyrido[2,3-e]tetrazolo[1,5-a]pyrimidin-5-yl)morpholine* (**4d**): observed in CDCl_3_ solution as a tautomer mixture in 1:4 ratio.

*Azide*: ^1^H NMR (500 MHz, CDCl_3_) δ 8.59 (dd, 1H, ^3^*J* = 4.1 Hz, ^4^*J* = 1.6 Hz, H-C(7)), 7.96 (dd, 1H, ^3^*J* = 8.5 Hz, ^4^*J* = 1.6 Hz, H-C(9)), 7.55 (dd, 1H, ^3^*J* = 8.5, 4.1 Hz, H-C(8)), 4.34–4.81 (m, 4H, 4 × H-C(1′)), 3.86-3.93 (m, 4H, 4 × H-C(2′)) ppm.

*Tetrazole*: ^1^H NMR (500 MHz, CDCl_3_) δ 8.91 (dd, 1H, ^3^*J* = 4.4 Hz, ^4^*J* = 1.5 Hz, H-C(7)), 8.79 (dd, 1H, ^3^*J* = 8.5 Hz, ^4^*J* = 1.5 Hz, H-C(9)), 7.88 (dd, 1H, ^3^*J* = 8.5, 4.4 Hz, H-C(8)), 4.34–4.81 (m, 4H, 4 × H-C(1′)), 3.86-3.93 (m, 4H, 4 × H-C(2′)) ppm.

*Pyrido[2,3-e]tetrazolo[1,5-a]pyrimidin-5-amine* (**4e**):

Methanolic ammonia (100 µL, *w* = 25%) was added to a solution of 2,4-diazidopyrido[3,2-*d*]pyrimidine (**2**) (50 mg, 0.235 mmol, 1 eq) dissolved in DCM (1 mL) in a 10 mL glass vial and reaction mixture was stirred overnight. After the reaction completion (HPLC monitoring), crude mixture was evaporated under reduced pressure and crystallized from pyridine to yield brown crystals (27 mg, 61%, m.p. 270 °C).

^1^H NMR (500 MHz, DMSO-*d*_6_) δ 8.98 (d, 1H, ^3^*J* = 4.5 Hz, H-C(7)), 8.77 (d, 1H, ^3^*J* = 8.4 Hz, H-C(9)), 8.62 (bs, 1H, H-N), 8.47 (bs, 1H, H-N), 8.07 (dd, 1H, ^3^*J* = 8.4, 4.5 Hz, H-C(8)) ppm. ^13^C NMR (125 MHz, DMSO-*d*_6_) δ 159.8, 153.7, 149.3, 129.5, 129.2, 128.8, 124.4 ppm.

IR (KBr): 3368, 3303, 3168, 3074, 2480, 1665, 1596, 1545, 1509 cm^−1^.

HRMS calculated for [C_7_H_5_N_7_ + H^+^] = 188.0679, found 188.0684.

*5-Hydrazinylpyrido[2,3-e]tetrazolo[1,5-a]pyrimidine* (**4f**):

Hydrazine hydrate (50 mg, 1g/mL, 50 µL, 1.560 mmol, 6.6 eq) was added to a solution of 2,4-diazidopyrido[3,2-*d*]pyrimidine (**2**) (50 mg, 0.235 mmol, 1 eq) dissolved in DCM (1 mL) in a 10 mL glass vial and stirred for 15 min at ambient temperature. After the reaction completion (HPLC monitoring), the crude mixture was washed with distilled water and MTBE to yield an orange amorphous solid (27 mg, 61%).

^1^H NMR (500 MHz, DMSO-*d*_6_) δ 10.49 (bs, 1H, H-N), 8.91 (d, 1H, ^3^*J* = 4.6 Hz, H-C(7)), 8.71 (d, 1H, ^3^*J* = 8.5 Hz, H-C(9)), 8.01 (dd, 1H, ^3^*J* = 8.5, 4.6 Hz, H-C(8)), 5.13 (bs, 2H, H_2_-N) ppm. ^13^C NMR (125 MHz, DMSO-*d*_6_) δ 154.5, 154.0, 149.1, 129.0, 128.8, 128.7, 124.4 ppm.

IR (KBr): 3324, 3234, 3059, 1615, 1596, 1573, 1538, 1517 cm^−1^.

HRMS calculated for [C_7_H_6_N_8_ + H^+^] = 203.0788, found 203.0806.

*5-(4-Methylpiperazin-1-yl)pyrido[2,3-e]tetrazolo[1,5-a]pyrimidine* (**4g**)*:*

*N*-Methylpiperazine (70 mg, 0.90 g/mL, 78 µL, 0.705 mmol, 3 eq) was added to a solution of 2,4-diazidopyrido[3,2-*d*]pyrimidine (**2**) (50 mg, 0.235 mmol, 1 eq) dissolved in DCM (1 mL) in a 10 mL glass vial and stirred for 15 min at ambient temperature. After the reaction completion (HPLC monitoring), the crude mixture was evaporated under reduced pressure and purified by silica gel column chromatography (DCM/MeOH, gradient 0→2→5%) to yield pale red crystals (48 mg, 77%, R_f_ = 0.15 in DCM/MeOH 5%, m.p. 185 °C).

^1^H NMR (500 MHz, DMSO-*d*_6_) δ 8.98 (dd, 1H, ^3^*J* = 4.4 Hz, ^4^*J* = 1.5 Hz, H-C(7)), 8.81 (dd, 1H, ^3^*J* = 8.5 Hz, ^4^*J* = 1.5 Hz, H-C(9)), 8.05 (dd, 1H, ^3^*J* = 8.5, 4.4 Hz, H-C(8)), 4.05–4.75 (m, 4H, 2 × H_2_-C(1′)), 2.51–2.56 (m, 4H, 2 × H_2_-C(2′)), 2.24 (s, 3H, H_3_-C(3′)) ppm. ^13^C NMR (125 MHz, DMSO-*d*_6_) δ 157.4, 152.7, 148.0, 130.8, 130.6, 128.5, 124.8, 54.7, 47.8, 45.5 ppm.

IR (KBr): 3077, 3047, 3012, 2913, 2856, 2804, 2774, 1607, 1591, 1542, 1527 cm^−1^.

HRMS calculated for [C_12_H_14_N_8_ + H^+^] = 271.1414, found 271.1436.

*2-Azido-4-(4-methylpiperazin-1-yl)pyrido[3,2-d]pyrimidine* (***4gA***) *and 5-(4-methylpiperazin-1-yl)pyrido[2,3-e]tetrazolo[1,5-a]pyrimidine* (***4g***): observed in CDCl_3_ solution as a tautomer mixture in a 21:79 ratio.

*Azide*: ^1^H NMR (500 MHz, CDCl_3_) δ 8.59 (dd, 1H, ^3^*J* = 4.1 Hz, ^4^*J* = 1.7 Hz, H-C(6)), 7.94 (dd, 1H, ^3^*J* = 8.5 Hz, ^4^*J* = 1.7 Hz, H-C(8)), 7.54 (dd, 1H, ^3^*J* = 8.5, 4.1 Hz, H-C(7)), 4.15–4.95 (m, 4H, 2 × H_2_-C(1′)), 2.57–2.61 (m, 4H, 2 × H_2_-C(2′)), 2.36 (s, 3H, H_3_-C(3′)) ppm.

*Tetrazole*: ^1^H NMR (500 MHz, CDCl_3_) δ 8.92 (dd, 1H, ^3^*J* = 4.4 Hz, ^4^*J* = 1.6 Hz, H-C(7)), 8.78 (dd, 1H, ^3^*J* = 8.4 Hz, ^4^*J* = 1.6 Hz, H-C(9)), 7.85 (dd, 1H, ^3^*J* = 8.4, 4.4 Hz, H-C(8)), 4.15–4.95 (m, 4H, 2 × H_2-_C(1′)), 2.61–2.65 (m, 4H, 2 × H_2_-C(2′)), 2.37, (s, 3H, H_3_-C(3′)) ppm.

*5-(Cyclopentyloxy)pyrido[2,3-e]tetrazolo[1,5-a]pyrimidine* (**5a**)*:*

Cyclopentanol (24 mg, 0.28 mmol, 1.2 eq) was added to 2,4-diazidopyrido[3,2-*d*]pyrimidine (**2**) (50 mg, 0.23 mmol, 1 eq) and K_2_CO_3_ (39 mg, 0.28 mmol, 1.2 eq) solution in MeCN (1 mL) under a N_2_ atmosphere and the resulting reaction mixture was stirred at 80 °C for 3 days. Water (10 mL) was added to the reaction mixture and extracted with DCM (3 × 5 mL). The combined organic phase was washed with saturated aqueous NaCl solution (2 × 10 mL), dried over anhydrous Na_2_SO_4_, filtered, and evaporated under reduced pressure. The crude product was purified by silica gel column chromatography (DCM/MeOH, gradient 0→1%) to yield a white amorphous solid (19 mg, 32%, R_f_ = 0.50 in 5% DCM/MeOH).

^1^H NMR (500 MHz, DMSO-*d*_6_) δ 9.09 (dd, 1H, ^3^*J* = 4.5 Hz, ^4^*J* = 1.5 Hz, H-C(7)), 8.91 (dd, 1H, ^3^*J* = 8.5 Hz, ^4^*J* = 1.5 Hz, H-C(9)), 8.14 (dd, 1H, ^3^*J* = 8.5, 4.5 Hz, H-C(8)), 5.78 (tt, 1H, ^3^*J* = 6.1, 3.0 Hz, H-C(1′)), 2.08–2.17 (m, 2H, 2 × H-CH), 1.94–2.02 (m, 2H, 2 × H-CH), 1.76–1.85 (m, 2H, 2 × H-CH), 1.65–1.74 (m, 2H, 2 × H-CH) ppm. ^13^C NMR (125 MHz, DMSO-*d*_6_) δ 163.5, 152.3, 150.5, 130.9, 129.7, 129.6, 124.4, 81.7, 32.2, 23.6 ppm.

IR (KBr): 3065, 3041, 2975, 2945, 2874, 1605, 1542 cm^−1^.

HRMS calculated for [C_12_H_12_N_6_O + H^+^] = 257.1145, found 257.1167.

*2-Azido-4-(cyclopentyloxy)pyrido[3,2-d]pyrimidine* (**5aA**) *and 5-(cyclopentyloxy)pyrido[2,3-e]tetrazolo[1,5-a]pyrimidine* (**5a**): observed in CDCl_3_ solution as a tautomer mixture in 7:93 ratio.

*Azide*: ^1^H NMR (500 MHz, CDCl_3_) δ 8.87 (dd, 1H, ^3^*J* = 4.1 Hz, ^4^*J* = 1.5 Hz, H-C(6)), 8.08 (dd, 1H, ^3^*J* = 8.5 Hz, ^4^*J* = 1.5 Hz, H-C(8)), 7.69 (dd, 1H, ^3^*J* = 8.5, 4.1 Hz, H-C(7)), 5.76 (tt, 1H, ^3^*J* = 6.6, 3.6 Hz, H-C(1′)), 2.19–2.28 (m, 2H, 2 × H-CH), 2.04–2.13 (m, 2H, 2 × H-CH), 1.88–1.98 (m, 2H, 2 × H-CH), 1.68–1.77 (m, 2H, 2 × H-CH) ppm.

*Tetrazole*: ^1^H NMR (500 MHz, CDCl_3_) δ 9.13 (dd, 1H, ^3^*J* = 4.6 Hz, ^4^*J* = 1.5 Hz, H-C(7)), 8.85 (dd, 1H, ^3^*J* = 8.4 Hz, ^4^*J* = 1.5 Hz, H-C(9)), 7.97 (dd, 1H, ^3^*J* = 8.4, 4.6 Hz, H-C(8)), 5.95 (tt, 1H, ^3^*J* = 6.5, 3.6 Hz, H-C(1′)), 2.19–2.28 (m, 2H, 2 × H-CH), 2.04–2.13 (m, 2H, 2 × H-CH), 1.88–1.98 (m, 2H, 2 × H-CH), 1.68–1.77 (m, 2H, 2 × H-CH) ppm.

*5-Propoxypyrido[2,3-e]tetrazolo[1,5-a]pyrimidine* (**5b**)*:*

*n*-Propanol (17 mg, 0.28 mmol, 1.2 eq) was added to 2,4-diazidopyrido[3,2-*d*]pyrimidine (**2**) (50 mg, 0.23 mmol, 1 eq) and K_2_CO_3_ (39 mg, 0.28 mmol, 1.2 eq) solution in MeCN (1 mL) under a N_2_ atmosphere and the resulting reaction mixture was stirred at 80 °C for 3 days. Water (10 mL) was added to the reaction mixture and extracted with DCM (3 × 5 mL). The combined organic phase was washed with a saturated aqueous NaCl solution (2 × 10 mL), dried over anhydrous Na_2_SO_4_, filtered, and evaporated under reduced pressure. The crude product was purified by silica gel column chromatography (DCM/MeOH, gradient 0→1%) to yield a white amorphous solid (11 mg, 20%, R_f_ = 0.65 in 5% DCM/MeOH).

^1^H NMR (500 MHz, DMSO-*d*_6_) δ 9.09 (dd, 1H, ^3^*J* = 4.5 Hz, ^4^*J* = 1.5 Hz, H-C(7)), 8.91 (dd, 1H, ^3^*J* = 8.5 Hz, ^4^*J* = 1.5 Hz, H-C(9)), 8.15 (dd, 1H, ^3^*J* = 8.5, 4.5 Hz, H-C(8)), 4.63 (t, 2H, ^3^*J* = 6.7 Hz, H-C(1′)), 1.93 (qt, 2H, ^3^*J* = 7.4, 6.7 Hz, H-C(2′)), 1.07 (t, 3H, ^3^*J* = 7.4 Hz, H-C(3′)) ppm. ^13^C NMR (125 MHz, DMSO-*d*_6_) δ 164.0, 152.3, 150.6, 131.0, 129.7, 129.5, 124.4, 70.3, 21.4, 10.4 ppm.

IR (KBr): 3062, 2974, 2942, 2884, 1607, 1543 cm^−1^.

HRMS calculated for [C_10_H_10_N_6_O + H^+^] = 231.0989, found 231.0991.

*2-Azido-4-propoxypyrido[3,2-d]pyrimidine* (***5bA***) *and 5-propoxypyrido[2,3-e]tetrazolo[1,5-a]pyrimidine* (***5b***): observed in CDCl_3_ solution as a tautomer mixture in a 2:23 ratio.

*Azide*: ^1^H NMR (500 MHz, CDCl_3_) δ 8.87 (dd, 1H, ^3^*J* = 4.2 Hz, ^4^*J* = 1.5 Hz, H-C(6)), 8.08 (dd, 1H, ^3^*J* = 8.6 Hz, ^4^*J* = 1.5 Hz, H-C(8)), 7.70 (dd, 1H, ^3^*J* = 8.6, 4.2 Hz, H-C(7)), 4.63 (t, 2H, ^3^*J* = 7.0 Hz, H-C(1′)), 2.01 (sextet, 2H, ^3^*J* = 7.2 Hz, H-C(2′)), 1.08 (t, 3H, ^3^*J* = 7.5 Hz, H-C(3′)) ppm.

*Tetrazole*: ^1^H NMR (500 MHz, CDCl_3_) δ 9.13 (dd, 1H, ^3^*J* = 4.5 Hz, ^4^*J* = 1.5 Hz, H-C(7)), 8.86 (dd, 1H, ^3^*J* = 8.5 Hz, ^4^*J* = 1.5 Hz, H-C(9)), 8.00 (dd, 1H, ^3^*J* = 8.5, 4.5 Hz, H-C(8)), 4.80 (t, 2H, ^3^*J* = 7.0 Hz, H-C(1′)), 2.06 (sextet, 2H, ^3^*J* = 7.2 Hz, H-C(2′)), 1.11 (t, 3H, ^3^*J* = 7.2 Hz, H-C(3′)) ppm.

*N-(4-Methoxybenzyl)-2-(4-phenyl-1H-1,2,3-triazol-1-yl)pyrido[3,2-d]pyrimidin-4-amine* (**6a**)*:*

Prepared according to procedure C using *N*-(4-methoxybenzyl)pyrido[2,3-*e*]tetrazolo[1,5-*a*]pyrimidin-5-amine (**4a**) (42 mg, 0.137 mmol, 1 eq), phenylacetylene (21 mg, *d* = 0.93 g/mL, v = 23 µL, 0.205 mmol, 1.5 eq), CuSO_4_·5H_2_O (7 mg, 0.027 mmol, 0.2 eq), sodium ascorbate (11 mg, 0.055 mmol, 0.4 eq), and triethylamine (28 mg, *d* = 0.73 g/mL, v = 38 µL, 0.273 mmol, 2 eq). Purified by silica gel column chromatography (DCM/MeOH, gradient 0→1%) to yield white amorphous solid (46 mg, 82%, R_f_ = 0.55 in 5% DCM/MeOH).

^1^H NMR (500 MHz, DMSO-*d*_6_) δ 9.64 (t, 1H, ^3^*J* = 6.4 Hz, H-N), 9.36 (s, 1H, H-C(1″)), 8.84 (dd, 1H, ^3^*J* = 4.3 Hz, ^4^*J* = 1.5 Hz, H-C(6)), 8.22 (dd, 1H, ^3^*J* = 8.5 Hz, ^4^*J* = 1.5 Hz, H-C(8)), 8.07 (d, 2H, ^3^*J* = 7.7 Hz, 2 × H-C(2″)), 7.90 (dd, 1H, ^3^*J* = 8.5, 4.3 Hz, H-C(7)), 7.51 (t, 2H, ^3^*J* = 7.7 Hz, 2 × H-C(3″)), 7.49 (d, 2H, ^3^*J* = 8.7 Hz, 2 × H-C(2′)), 7.40 (t, 1H, ^3^*J* = 7.7 Hz, H-C(4″)), 6.88 (d, 2H, ^3^*J* = 8.7 Hz, 2 × H-C(3′)), 4.85 (d, 2H, ^3^*J* = 6.4 Hz, H_2_-C(1′)), 3.69 (s, 3H, H_3_-CO) ppm. ^13^C NMR (125 MHz, DMSO-*d*_6_) δ 160.6, 158.4, 150.8, 148.6, 146.5, 144.8, 135.4, 131.2, 130.8, 130.1, 129.5, 129.1, 129.0, 128.3, 125.6, 120.0, 113.7, 55.0, 43.3 ppm.

^1^H NMR (500 MHz, CDCl_3_) δ 8.84 (s, 1H, H-C(1″)), 8.69 (dd, 1H, ^3^*J* = 4.2 Hz, ^4^*J* = 1.4 Hz, H-C(6)), 8.27 (dd, 1H, ^3^*J* = 8.5 Hz, ^4^*J* = 1.4 Hz, H-C(8)), 7.99 (d, 2H, ^3^*J* = 7.8 Hz, 2x H-C(2″)), 7.72 (dd, 1H, ^3^*J* = 8.5, 4.2 Hz, H-C(7)), 7.72 (s, 1H, H-N), 7.47 (t, 2H, ^3^*J* = 7.8 Hz, 2 × H-C(3″)), 7.42 (d, 2H, ^3^*J* = 8.7 Hz, 2 × H-C(2′)), 7.38 (t, 1H, ^3^*J* = 7.8 Hz, H-C(4″)), 6.93 (d, 2H, ^3^*J* = 8.7 Hz, 2 × H-C(3′)), 4.91 (d, 2H, ^3^*J* = 5.8 Hz, H_2_-C(1′)), 3.81 (s, 3H, H_3_-CO) ppm. ^13^C NMR (125 MHz, CDCl_3_) δ 161.1, 159.6, 151.4, 148.5, 147.7, 145.3, 136.2, 131.5, 130.5, 129.7, 129.3, 129.0, 128.7, 128.5, 126.2, 119.0, 114.5, 55.5, 45.0 ppm.

IR (KBr): 2930, 1609, 1593, 1513 cm^−1^.

HRMS calculated for [C_23_H_19_N_7_O + H^+^] = 410.1724, found 410.1730.

*N-(4-Methoxybenzyl)-2-(4-(p-tolyl)-1H-1,2,3-triazol-1-yl)pyrido[3,2-d]pyrimidin-4-amine* (**6b**)*:*

Prepared according to procedure C using *N*-(4-methoxybenzyl)pyrido[2,3-*e*]tetrazolo[1,5-*a*]pyrimidin-5-amine (**4a**) (71 mg, 0.231 mmol, 1 eq), tolylacetylene (40 mg, *d* = 0.92 g/mL, v = 44 µL, 0.347 mmol, 1.5 eq), CuSO_4_·5H_2_O (12 mg, 0.046 mmol, 0.2 eq), sodium ascorbate (18 mg, 0.092 mmol, 0.4 eq), and triethylamine (47 mg, *d* = 0.73 g/mL, v = 64 µL, 0.462 mmol, 2 eq). Purified by silica gel column chromatography (DCM/MeOH, gradient 0→1%) to yield a white amorphous solid (70 mg, 71%, R_f_ = 0.60 in 5% DCM/MeOH).

^1^H NMR (500 MHz, DMSO-*d*_6_) δ 9.63 (t, 1H, ^3^*J* = 6.3 Hz, H-N), 9.29 (s, 1H, H-C(1″)), 8.84 (dd, 1H, ^3^*J* = 4.3 Hz, ^4^*J* = 1.5 Hz, H-C(6)), 8.22 (dd, 1H, ^3^*J* = 8.4 Hz, ^4^*J* = 1.5 Hz, H-C(8)), 7.95 (d, 2H, ^3^*J* = 8.1 Hz, 2 × H-C(2″)), 7.90 (dd, 1H, ^3^*J* = 8.4, 4.3 Hz, H-C(7)), 7.49 (d, 2H, ^3^*J* = 8.7 Hz, 2 × H-C(2′)), 7.31 (d, 2H, ^3^*J* = 8.1 Hz, 2 × H-C(3″)), 6.88 (d, 2H, ^3^*J* = 8.7 Hz, 2 × H-C(3′)), 4.84 (d, 2H, ^3^*J* = 6.3 Hz, H_2_-C(1′)), 3.69 (s, 3H, H_3_-CO), 2.36 (s, 3H, H_3_-C(4″)) ppm. ^13^C NMR (125 MHz, DMSO-*d*_6_) δ 160.6, 158.4, 150.8, 148.6, 146.5, 144.8, 137.7, 135.3, 131.2, 130.8, 129.5 (2 × C), 129.1, 127.3, 125.5, 119.5, 113.7, 55.0, 43.3, 20.9 ppm.

^1^H NMR (500 MHz, CDCl_3_) δ 8.80 (s, 1H, H-C(1″)), 8.68 (dd, 1H, ^3^*J* = 4.3 Hz, ^4^*J* = 1.5 Hz, H-C(6)), 8.26 (dd, 1H, ^3^*J* = 8.4 Hz, ^4^*J* = 1.5 Hz, H-C(8)), 7.87 (d, 2H, ^3^*J* = 8.0 Hz, 2 × H-C(2″)), 7.71 (dd, 1H, ^3^*J* = 8.4, 4.3 Hz, H-C(7)), 7.71 (s, 1H, H-N), 7.41 (d, 2H, ^3^*J* = 8.7 Hz, 2 × H-C(2′)), 7.28 (d, 2H, ^3^*J* = 8.0 Hz, 2 × H-C(3″)), 6.92 (d, 2H, ^3^*J* = 8.7 Hz, 2 × H-C(3′)), 4.91 (d, 2H, ^3^*J* = 5.8 Hz, H_2_-C(1′)), 3.81 (s, 3H, H_3_-CO), 2.41 (s, 3H, H_3_-C(4″)) ppm. ^13^C NMR (125 MHz, CDCl_3_) δ 161.1, 159.6, 151.4, 148.4, 147.8, 145.3, 138.4, 136.2, 131.5, 129.7 (2 × C), 129.4, 128.7, 127.6, 126.1, 118.6, 114.5, 55.5, 44.9, 21.5 ppm.

IR (KBr): 3163, 3058, 2921, 2834, 1609, 1594, 1563, 1511 cm^−1^.

HRMS calculated for [C_24_H_21_N_7_O + H^+^] = 424.1880, found 424.1897.

*4-(1-(4-((4-Methoxybenzyl)amino)pyrido[3,2-d]pyrimidin-2-yl)-1H-1,2,3-triazol-4-yl)benzonitrile* (**6c**)*:*

Prepared according to procedure C using *N*-(4-methoxybenzyl)pyrido[2,3-*e*]tetrazolo[1,5-*a*]pyrimidin-5-amine (**4a**) (50 mg, 0.162 mmol, 1 eq), *p*-cyanophenylacetylene (31 mg, 0.243 mmol, 1.5 eq), CuSO_4_·5H_2_O (8 mg, 0.032 mmol, 0.2 eq), sodium ascorbate (13 mg, 0.065 mmol, 0.4 eq), and triethylamine (33 mg, *d* = 0.73 g/mL, v = 45 µL, 0.324 mmol, 2 eq). Purified by silica gel column chromatography (DCM/MeOH, gradient 0→1%) to yield a white amorphous solid (49 mg, 69%, R_f_ = 0.70 in 5% DCM/MeOH). A single crystal for X-ray analysis was obtained by slow evaporation from CHCl_3_/MeOH.

^1^H NMR (500 MHz, DMSO-*d*_6_) δ 9.67 (t, 1H, ^3^*J* = 6.3 Hz, H-N), 9.61 (s, 1H, H-C(1″)), 8.85 (dd, 1H, ^3^*J* = 4.3 Hz, ^4^*J* = 1.5 Hz, H-C(6)), 8.28 (d, 2H, ^3^*J* = 8.2 Hz, 2 × H-C(2″)), 8.23 (dd, 1H, ^3^*J* = 8.5 Hz, ^4^*J* = 1.5 Hz, H-C(8)), 7.98 (d, 2H, ^3^*J* = 8.2 Hz, 2 × H-C(3″)), 7.91 (dd, 1H, ^3^*J* = 8.5, 4.3 Hz, H-C(7)), 7.49 (d, 2H, ^3^*J* = 8.7 Hz, 2 × H-C(2′)), 6.88 (d, 2H, ^3^*J* = 8.7 Hz, 2 × H-C(3′)), 4.86 (d, 2H, ^3^*J* = 6.3 Hz, H_2_-C(1′)), 3.69 (s, 3H, H_3_-CO) ppm. ^13^C NMR (125 MHz, DMSO-*d*_6_) δ 160.6, 158.4, 150.6, 148.8, 144.9, 144.7, 135.4, 134.6, 133.0, 131.3, 130.7, 129.6, 129.1, 126.2, 121.8, 118.8, 113.7, 110.6, 55.0, 43.3 ppm.

^1^H NMR (500 MHz, CDCl_3_) δ 8.93 (s, 1H, H-C(1″)), 8.71 (dd, 1H, ^3^*J* = 4.3 Hz, ^4^*J* = 1.5 Hz, H-C(6)), 8.26 (dd, 1H, ^3^*J* = 8.5 Hz, ^4^*J* = 1.5 Hz, H-C(8)), 8.10 (d, 2H, ^3^*J* = 8.4 Hz, 2 × H-C(2″)), 7.76 (d, 2H, ^3^*J* = 8.4 Hz, 2 × H-C(3″)), 7.75 (t, 1H, ^3^*J* = 5.8 Hz, H-N), 7.73 (dd, 1H, ^3^*J* = 8.5, 4.3 Hz, H-C(7)), 7.41 (d, 2H, ^3^*J* = 8.7 Hz, 2 × H-C(2′)), 6.93 (d, 2H, ^3^*J* = 8.7 Hz, 2 × H-C(3′)), 4.91 (d, 2H, ^3^*J* = 5.8 Hz, H_2_-C(1′)), 3.82 (s, 3H, H_3_-CO) ppm. ^13^C NMR (125 MHz, CDCl_3_) δ 161.1, 159.6, 151.2, 148.7, 145.9, 145.2, 136.2, 134.9, 132.9, 131.5, 129.6, 129.1, 128.8, 126.6, 120.2, 118.9, 114.5, 111.9, 55.5, 45.0 ppm.

IR (KBr): 3414, 2223, 1610, 1586, 1556, 1510 cm^−1^.

HRMS calculated for [C_24_H_18_N_8_O + H^+^] = 435.1676, found 435.1694.

*2-(4-Hexyl-1H-1,2,3-triazol-1-yl)-N-(4-methoxybenzyl)pyrido[3,2-d]pyrimidin-4-amine* (**6d**)*:*

Prepared according to procedure C using *N*-(4-methoxybenzyl)pyrido[2,3-*e*]tetrazolo[1,5-*a*]pyrimidin-5-amine (**4a**) (71 mg, 0.231 mmol, 1 eq), 1-octyne (38 mg, *d* = 0.72 g/mL, v = 53 µL, 0.347 mmol, 1.5 eq), CuSO_4_·5H_2_O (12 mg, 0.046 mmol, 0.2 eq), sodium ascorbate (18 mg, 0.092 mmol, 0.4 eq), and triethylamine (47 mg, *d* = 0.73 g/mL, v = 64 µL, 0.462 mmol, 2 eq). Purified by silica gel column chromatography (DCM/MeOH, gradient 0→1%) to yield aa white amorphous solid (80 mg, 83%, R_f_ = 0.55 in 5% DCM/MeOH).

^1^H NMR (500 MHz, DMSO-*d*_6_) δ 9.61 (t, 1H, ^3^*J* = 6.3 Hz, H-N), 8.82 (dd, 1H, ^3^*J* = 4.3 Hz, ^4^*J* = 1.5 Hz, H-C(6)), 8.60 (s, 1H, H-C(1″)), 8.18 (dd, 1H, ^3^*J* = 8.4, ^4^*J* = 1.5 Hz, H-C(8)), 7.88 (dd, 1H, ^3^*J* = 8.4, 4.3 Hz, H-C(7)), 7.45 (d, 2H, ^3^*J* = 8.7 Hz, 2 × H-C(2′)), 6.86 (d, 2H, ^3^*J* = 8.7 Hz, 2 × H-C(3′)), 4.77 (d, 2H, ^3^*J* = 6.3 Hz, H_2_-C(1′)), 3.70 (s, 3H, H_3_-CO), 2.73 (t, 2H, ^3^*J* = 7.4 Hz, H-C(2″)), 1.68 (quintet, 2H, ^3^*J* = 7.4 Hz), 1.25–1.40 (m, 6H, H_2_-C(4″), H_2_-C(5″), H_2_-C(6″)), 0.87 (t, 3H, ^3^*J* = 7.0 Hz, H-C(7″)) ppm. ^13^C NMR (125 MHz, DMSO-*d*_6_) δ 160.6, 158.4, 150.8, 148.5, 147.4, 144.8, 135.3, 131.1, 130.7, 129.4, 129.0, 120.7, 113.7, 55.0, 43.3, 31.0, 28.7, 28.2, 24.8, 22.0, 13.9 ppm.

^1^H NMR (500 MHz, CDCl_3_) δ 8.67 (dd, 1H, ^3^*J* = 4.3 Hz, ^4^*J* = 1.5 Hz, H-C(6)), 8.36 (s, 1H, H-C(1″)), 8.25 (dd, 1H, ^3^*J* = 8.5 Hz, ^4^*J* = 1.5 Hz, H-C(8)), 7.69 (dd, 1H, ^3^*J* = 8.5, 4.3 Hz, H-C(7)), 7.67 (t, 1H, ^3^*J* = 5.8 Hz, H-N), 7.39 (d, 2H, ^3^*J* = 8.7 Hz, 2 × H-C(2′)), 6.92 (d, 2H, ^3^*J* = 8.7 Hz, 2 × H-C(3′)), 4.88 (d, 2H, ^3^*J* = 5.8 Hz, H_2_-C(1′)), 3.81 (s, 3H, H_3_-CO), 2.83 (t, 2H, ^3^*J* = 7.6 Hz, H-C(2″)), 1.76 (quintet, 2H, ^3^*J* = 7.6 Hz, H-C(3″)), 1.30–1.45 (m, 6H, H_2_-C(4″), H_2_-C(5″), H_2_-C(6″)), 0.89 (t, 3H, ^3^*J* = 7.0 Hz, H-C(7″) ppm. ^13^C NMR (125 MHz, CDCl_3_) δ 161.0, 159.5, 151.5, 148.6, 148.3, 145.3, 136.2, 131.4, 129.6, 129.4, 128.6, 120.2, 114.5, 55.5, 44.9, 31.8, 29.5, 29.1, 25.9, 22.7, 14.2 ppm.

IR (KBr): 3431, 3351, 3057, 2928, 2856, 1651, 1609, 1591, 1568, 1557, 1511 cm^−1^.

HRMS calculated for [C_23_H_27_N_7_O + H^+^] = 418.2350, found 418.2362.

*Methyl 1-(4-((4-methoxybenzyl)amino)pyrido[3,2-d]pyrimidin-2-yl)-1H-1,2,3-triazole-4-carboxylate* (**6e**)*:*

Prepared according to procedure C using *N*-(4-methoxybenzyl)pyrido[2,3-*e*]tetrazolo[1,5-*a*]pyrimidin-5-amine (**4a**) (52 mg, 0.169 mmol, 1 eq), methylpropionate (21 mg, *d* = 0.95 g/mL, v = 23 µL, 0.254 mmol, 1.5 eq), CuSO_4_·5H_2_O (8 mg, 0.034 mmol, 0.2 eq), sodium ascorbate (13 mg, 0.068 mmol, 0.4 eq), and triethylamine (34 mg, *d* = 0.73 g/mL, v = 47 µL, 0.338 mmol, 2 eq). Purified by silica gel column chromatography (DCM/MeOH, gradient 0→1%) and crystallization from *n*-PrOH to yield white crystals (13 mg, 20%, R_f_ = 0.50 in 5% DCM/MeOH, m.p. 154 °C).

^1^H NMR (500 MHz, CDCl_3_) δ 9.17 (s, 1H, H-C(1″)), 8.71 (dd, 1H, ^3^*J* = 4.3 Hz, ^4^*J* = 1.5 Hz, H-C(6)), 8.28 (dd, 1H, ^3^*J* = 8.5 Hz, ^4^*J* = 1.5 Hz, H-C(8)), 7.76 (t, 1H, ^3^*J* = 5.8 Hz, H-N), 7.73 (dd, 1H, ^3^*J* = 8.5, 4.3 Hz, H-C(7)), 7.38 (d, 2H, ^3^*J* = 8.6 Hz, 2 × H-C(2′)), 6.92 (d, 2H, ^3^*J* = 8.6 Hz, 2 × H-C(3′)), 4.88 (d, 2H, ^3^*J* = 5.8 Hz, H_2_-C(1′)), 4.02 (s, 3H, H_3_-C(2″)), 3.81 (s, 3H, H_3_-C(4′)) ppm. ^13^C NMR (125 MHz, CDCl_3_) δ 161.3, 161.1, 159.6, 150.9, 148.9, 145.1, 139.9, 136.3, 131.6, 129.6, 129.0, 128.9, 127.4, 114.5, 55.5, 52.5, 45.0 ppm.

IR (KBr): 3326, 3179, 3056, 2949, 1736, 1608, 1592, 1573, 1558, 1513 cm^−1^.

HRMS calculated for [C_19_H_17_N_7_O_3_ + H^+^] = 392.1466, found 392.1472.

*2-(4-Phenyl-1H-1,2,3-triazol-1-yl)pyrido[3,2-d]pyrimidin-4-amine* (**7**)*:*

2,4-Diazidopyrido[3,2-*d*]pyrimidine (50 mg, 0.235 mmol, 1 eq), phenylacetylene (120 mg, 1.175 mmol, 5 eq), sodium ascorbate (19 mg, 0.094 mmol, 0.4 eq), CuSO_4_·5H_2_O (12 mg, 0.047 mmol, 0.2 eq), and NEt_3_ (47 mg, *d* = 0.73 g/mL, 65 µL, 0.469 mmol, 2 eq) were dissolved in THF (1 mL), H_2_O (0.1 mL) in a 10 mL glass vial and stirred at 70 °C overnight. After the reaction completion (HPLC monitoring), the resulting mixture was filtered through the silica gel/Na_2_SO_4_ plug and evaporated under reduced pressure. The crude product was purified by silica gel column chromatography (DCM/MeOH, gradient 0→5→10%) to yield a white amorphous solid (10 mg, 15%, R_f_ = 0.20 in 5% DCM/MeOH).

^1^H NMR (500 MHz, DMSO-*d*_6_) δ 9.24 (s, 1H, H-C(1′)), 8.85 (dd, 1H, ^3^*J* = 4.3 Hz, ^4^*J* = 1.5 Hz, H-C(7)), 8.71 (s, 1H, H-N), 8.60 (s, 1H, H-N), 8.21 (dd, 1H, ^3^*J* = 8.5 Hz, ^4^*J* = 1.5 Hz, H-C(9)), 8.04 (d, 2H, ^3^*J* = 7.7 Hz, 2 × H-C(2′)), 7.91 (dd, 1H, ^3^*J* = 8.5, 1.5 Hz, H-C(8)), 7.50 (t, 2H, ^3^*J* = 7.7 Hz, 2 × H-C(3′)), 7.39 (t, 1H, ^3^*J* = 7.7 Hz, H-C(4′)) ppm. ^13^C NMR (125 MHz, DMSO-*d*_6_) δ 163.7, 151.2, 148.7, 146.4, 145.1, 135.2, 130.9, 130.0, 129.3, 129.0, 128.3, 125.6, 119.7 ppm.

IR (KBr): 3461, 3282, 3164, 1647, 1578, 1556, 1513 cm^−1^.

HRMS calculated for [C_15_H_11_N_7_ + H^+^] = 290.1149, found 290.1177.

*N-Hexyl-2-((triphenylphosphoronylidene)amino)pyrido[3,2-d]pyrimidin-4-amine* (**8**):

Triphenylphosphine (116 mg, 0.442 mmol, 1.5 eq) and *N*-hexylpyrido[2,3-*e*]tetrazolo[1,5-*a*]pyrimidin-5-amine (**4b**) (80 mg, 0.295mmol, 1 eq) were dissolved in DCM (1 mL) and stirred for 3 days at room temperature. The reaction mixture was evaporated under reduced pressure and the crude product was purified by silica gel column chromatography (DCM/MeOH, gradient 0→1→2%) and precipitation from DCM/MTBE as a white amorphous solid (48 mg, 32%, R_f_ = 0.20 in 5% DCM/MeOH). A crystalline sample of the product was obtained for its HCl salt, ((4-(hexylamino)pyrido[3,2-*d*]pyrimidin-2-(1*H*)-ylidene)amino)triphenylphosphonium chloride (**8′**), which was obtained by precipitation from HCl containing the DCM/MTBE system. A single crystal for X-ray analysis was obtained by slow evaporation from CHCl_3_.

^1^H NMR (500 MHz, DMSO-*d*_6_) δ 12.60 (s, 1H, H-N), 9.39 (t, 1H, ^3^*J* = 6.2 Hz, H-N), 8.57 (dd, 1H, ^3^*J* = 4.3 Hz, ^4^*J* = 1.5 Hz, H-C(6)), 7.85 (dd, 1H, ^3^*J* = 8.6 Hz, ^4^*J* = 1.5 Hz, H-C(8)), 7.82 (ddd, 6H, ^3^*J*_H-P_ = 12.6 Hz, ^3^*J* = 7.6 Hz, ^4^*J* = 1.4 Hz, 6 × H-C(1″)), 7.80 (dd, 1H, ^3^*J* = 8.6, 4.3 Hz, H-C(7)), 7.74 (tdt, 3H, ^3^*J* = 7.6 Hz, ^5^*J*_H-P_ = 1.7 Hz, ^4^*J* = 1.4 Hz, 3 × H-C(3″)), 7.64 (td, 6H, ^3^*J* = 7.6 Hz, ^4^*J*_H-P_ = 3.4 Hz, 6 × H-C(2″)), 2.81 (td, 2H, ^3^*J* = 7.5, 6.2 Hz, H_2_-C(1′)), 0.86–1.19 (m, 8H, H_2_-C(2′), H_2_-C(3′), H_2_-C(4′), H_2_-C(5′)), 0.82 (t, 3H, ^3^*J* = 7.4 Hz, H-C(6′)) ppm.

^1^H NMR (500 MHz, CDCl_3_) δ 14.08 (s, 1H, H-N), 8.89 (dd, 1H, ^3^*J* = 8.5 Hz, ^4^*J* = 1.4 Hz, H-C(6)), 8.36 (dd, 1H, ^3^*J* = 4.4 Hz, ^4^*J* = 1.4 Hz, H-C(8)), 7.80–7.86 (m, 6H, 6 × H-C(1″)), 7.57–7.63 (m, 3H, 3 × H-C(3″)), 7.53 (dd, 1H, ^3^*J* = 8.5, 4.4 Hz, H-C(7)), 7.48–7.54 (m, 6H, 6 × H-C(2″)), 7.37 (t, 1H, ^3^*J* = 6.1 Hz, H-N), 2.93 (td, 2H, ^3^*J* = 7.2, 6.1 Hz, H_2_-C(1′)), 1.11–1.37 (m, 8H, H_2_-C(2′), H_2_-C(3′), H_2_-C(4′), H_2_-C(5′)), 0.89 (t, 3H, ^3^*J* = 7.2 Hz, H-C(6′)) ppm. ^13^C NMR (125 MHz, CDCl_3_) δ 159.2, 156.9, 145.3, 137.8 (d, ^2^*J*_C-P_ = 4.4 Hz), 133.3 (d, ^3^*J*_C-P_ = 10.3 Hz), 132.9 (d, ^4^*J*_C-P_ = 2.8 Hz), 129.1, 129.1 (d, ^2^*J*_C-P_ = 12.8 Hz), 127.3, 127.2 (d, ^1^*J*_C-P_ = 103.3 Hz), 126.3, 41.0, 31.5, 29.0, 26.7, 22.7, 14.2 ppm. ^31^P NMR (202 MHz, CDCl_3_) δ 19.6 ppm.

IR (KBr): 3255, 3048, 2952, 2927, 2855, 2694, 1624, 1609, 1586, 1547, 1516 cm^−1^.

HRMS calculated for [C_31_H_32_N_5_P + H^+^] = 506.2468, found 506.2468.

*2-Chloro-N-hexylpyrido[3,2-d]pyrimidin-4-amine* (**9**):

*n*-Hexylamine (75 mg, 97 µL, 0.75 mmol, 3 eq) was added to 2,4-dichloropyrido[3,2-*d*]pyrimidine (**1**) (50 mg, 0.25 mmol, 1 eq) solution in DCM (1 mL) and stirred for 15 min. After the reaction completion (HPLC monitoring), an additional DCM (5 mL) was added and the mixture was washed with 0.5 M HCl_(aq.)_ solution (2 × 5 mL), followed by a saturated NaCl_(aq.)_ wash (2 × 5 mL). The organic phase was dried over anhydrous Na_2_SO_4_, filtered, and evaporated under reduced pressure. The crude product was purified by silica gel column chromatography (DCM/MeOH, gradient 0→1%) to yield a colorless oil (59 mg, 89%, R_f_ = 0.70 in 5% DCM/MeOH).

^1^H NMR (500 MHz, CDCl_3_) δ 8.66 (d, 1H, ^3^*J* = 4.5 Hz, H-C(6)), 8.01 (d, 1H, ^3^*J* = 8.4 Hz, H-C(8)), 7.64 (dd, 1H, ^3^*J* = 8.4, 4.5 Hz, H-C(7)), 7.33 (bs, 1H, H-N), 3.66 (q, 2H, ^3^*J* = 6.8 Hz, H_2_-C(1′)), 1.69–1.77 (m, 2H, H_2_-C(2′)), 1.45 (m, 2H, H_2_-C(3′)), 1.29–1.39 (m, 4H, H_2_-C(4′), H_2_-C(5′)), 0.90 (t, 3H, ^3^*J* = 6.8 Hz, H_3_-C(6′)) ppm. ^13^C NMR (125 MHz, CDCl_3_) δ 161.0, 158.7, 148.3, 145.6, 135.2, 130.9, 128.4, 41.2, 31.6, 29.3, 26.7, 22.7, 14.2 ppm.

HRMS calculated for [C_13_H_17_ClN_4_ + H^+^] = 265.1215, found 265.1194.

## 4. Conclusions

In conclusion, we found simple and effective reaction conditions for SnAr reactions of 2,4-diazidopyrido[3,2-*d*]pyrimidine with *N*-, *O*-, *S*- nucleophiles and obtained 5-substituted tetrazolo[1,5-*a*]pyrido[2,3-*e*]pyrimidines in high yields. The developed synthesis route via diazide is more rapid and convenient than the conventional route by stepwise substitution of the corresponding 2,4-dichloropyrido[3,2-*d*]pyrimidine. 2,4-Diazidopyrido[3,2-*d*]pyrimidine and the obtained tetrazolo[1,5-*a*]pyrido[2,3-*e*]pyrimidines exist in azide-tetrazole equilibrium in solutions favoring the tetrazole tautomer. The equilibrium is susceptible to solvent polarity, temperature, and substituents in the fused ring system. The calculated thermodynamic values of tautomerization (ΔG_298_ **=** −3.33 to −7.52 kJ/mol) assert a higher tetrazole stability compared to azido tautomers. Substituents with stronger electron-donating properties direct equilibrium toward the tetrazole tautomer. The obtained tetrazolo[1,5-*a*]pyrido[2,3-*e*]pyrimidines can be transformed into 1,2,3-triazoles via the CuAAC reaction using the CuSO_4_/ascorbate catalytic system. Finally, the single crystal X-ray analysis of tetrazolo[1,5-*a*]pyrido[2,3-*e*]pyrimidines depicts the tetrazole tautomer as the most stable form in the solid phase.

## Figures and Tables

**Table 1:** Crystal data and refinement details of compounds **2**, **3b**, **3f**, **4a**, **4d**, **6a**, and **8′**.

Compound	2	3b	3f	4a	4d	6a	8′
Structural formula	C_7_H_3_N_9_	C_31_H_26_Cl_2_N_12_S_2_	C_13_H_14_N_6_S	C_15_H_13_N_7_O	C_11_H_11_N_7_O	C_24_H_18_N_8_O	C_33_H_35_Cl_7_N_5_P
Molar weight (g/mol)	213.18	701.66	286.36	307.32	257.27	434.46	780.78
Crystal system	Monoclinic	Monoclinic	Orthorhombic	Monoclinic	Triclinic	Triclinic	Triclinic
Space group	*P*2_1_/*n*	*P*2/*n*	*Pbca*	*P*2_1_/*c*	*P*-1	*P*-1	*P*-1
*a* (Å)	7.7018 (2)	9.5365 (2)	5.8380 (1)	13.9255 (3)	6.8386 (3)	8.0329 (5)	9.51807 (7)
*b* (Å)	4.9727 (1)	6.9099 (2)	15.1883 (2)	8.3800 (2)	8.7709 (5)	10.3423 (4)	12.55333 (10)
*c* (Å)	22.0045 (4)	24.5953 (4)	30.4815 (4)	13.3706 (3)	10.4546 (5)	13.5771 (4)	16.07767 (12)
α (°)	90.00	90.00	90.00	90.00	67.195 (5)	109.489 (3)	96.7786 (7)
β (°)	92.956 (2)	94.787 (2)	90.00	115.495 (3)	81.477 (4)	97.968 (3)	98.3660 (6)
γ (°)	90.00	90.00	90.00	90.00	71.424 (4)	100.796 (4)	98.8468 (6)
*V* (Å^3^)	841.62 (3)	1615.08 (6)	2702.77(7)	1408.35 (6)	547.72 (5)	1019.59 (8)	1858.49 (2)
Z	4	2	8	4	2	2	2
T (K)	160(2)	150(2)	200(2)	150(2)	160(2)	150(2)	150(2)
Absorption coefficient (mm^−1^)	1.04	3.38	2.13	0.82	0.92	0.76	5.53
Calculated density (mg/m^3^)	1.682	1.443	1.407	1.449	1.560	1.415	1.395
Data collected	7965	15480	24550	13282	9722	12025	35065
*θ*-range for data collection (°)	4.0–76.3	3.6–76.4	2.9–76.1	3.5–75.9	4.6–76.1	3.5–76.4	2.8–76.6
Unique reflections	1491	3180	2757	2735	2171	3983	7454
Symmetry factor (*R_int_)*	0.024	0.037	0.043	0.047	0.051	0.032	0.036
R_sigma_	0.017	0.029	0.020	0.037	0.034	0.031	0.026
Final *R*_1_ factor for *I* > 2σ(*I*)	0.034	0.048	0.033	0.041	0.052	0.037	0.037
wR2 factor for all data	0.094	0.132	0.093	0.108	0.160	0.104	0.103
CCDC deposition number	2208559	2208560	2208556	2208558	2208557	2208561	2208562

**Table 2:** The selected bond lengths for the studied crystal structures and data from the literature *.

	N1-N2	N1-N10	N2-N3	N3-C3a	C3a-N4	C3a-N10	N4-C5
**2**	1.301(1)	1.357(1)	1.357(1)	1.328(1)	1.356(1)	1.357(1)	1.304(1)
**3b**	1.308(3)	1.356(3)	1.364(3)	1.334(3)	1.354(3)	1.354(3)	1.308(3)
**3f**	1.306(2)	1.355(2)	1.358(2)	1.325(2)	1.359(2)	1.357(2)	1.308(2)
**4a**	1.298(2)	1.362(2)	1.359(2)	1.331(2)	1.354(2)	1.363(2)	1.324(2)
**4d**	1.301(2)	1.364(2)	1.348(2)	1.334(2)	1.341(2)	1.353(2)	1.329(2)
Ivajoh	1.304(2)	1.365(23)	1.350(2)	1.336(2)	1.343(2)	1.363(2)	1.324(2)
Pesvuh	1.305(2)	1.358(2)	1.347(2)	1.328(2)	1.350(2)	1.359(2)	1.303(2)
Pulsib	1.306(3)	1.369(3)	1.365(3)	1.330(3)	1.348(3)	1.368(3)	1.308(3)
Sowwik	1.307(7)	1.354(7)	1.367(8)	1.308(7)	1.369(8)	1.365(7)	1.315(8)
Uxazid	1.300(1)	1.351(1)	1.345(1)	1.326(1)	1.348(1)	1.363(1)	1.309(1)
Vendoh	1.289(4)	1.365(5)	1.343(4)	1.330(5)	1.343(4)	1.351(4)	1.316(4)

* Ivajoh [44], Pesvuh [45], Pulsib [46], Sowwik [47], Uxazid [48], Vendoh [49].

**Table 3:** Equilibrium constant and thermodynamic heat of the tautomerization of substituted tetrazolo[1,5-*a*]pyrido[2,3-*e*]pyrimidines **3**–**5** in CDCl_3_; (**A**) azide form, (**T**) tetrazole form.

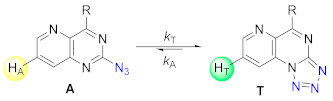						
Compound	R	T (K)	K_(eq)_ *	ΔG_298_ (kJ/mol)	ΔH_298_ (kJ/mol)	ΔS_298_ (J/mol·K)
**3a**	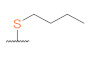	298	8.44	−5.29 ± 0.11	−32.11 ± 1.94	−90.14 ± 6.24
		313	4.32			
		323	3.11			
**3b**	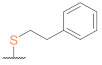	298	6.26	−4.54 ± 0.02	−23.63 ± 0.38	−64.08 ± 1.21
		313	3.92			
		323	2.99			
**3c**	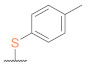	298	12.39	−6.24 ± 0.02	−30.53 ± 0.34	−81.69 ± 1.11
		313	6.37			
		323	4.81			
**3d**	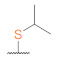	298	6.53	−4.65 ± 0.03	−20.14 ± 0.61	−51.96 ± 1.95
		313	4.49			
		323	3.47			
**3e**	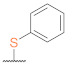	298	4.19	−3.55 ± 0.28	−31.75 ± 4.90	−94.33 ± 15.74
		313	2.57			
		323	1.53			
**3f**	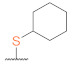	298	15.08	−6.11 ± 0.12	−42.05 ± 2.13	−120.72 ± 6.83
		313	4.96			
		323	3.30			
**4a**	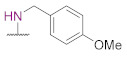	298	20.83	−7.52 ± 0.22	−21.91 ± 3.91	−48.05 ± 12.53
		313	15.05			
		323	10.39			
**4b**	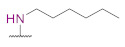	298	19.20	−7.32 ± 0.03	−20.35 ± 0.56	−43.74 ± 1.78
		313	12.77			
		323	10.19			
**4c**	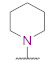	298	5.92	−4.40 ± 0.07	−24.52 ± 1.31	−67.42 ± 4.20
		313	3.81			
		323	2.74			
**4d**	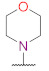	298	3.89	−3.36 ± 0.02	−22.65 ± 0.32	−64.71 ± 1.03
		313	2.53			
		323	1.91			
**4g**	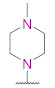	298	3.83	−3.33 ± 0.01	−19.92 ± 0.25	−55.69 ± 0.79
		313	2.59			
		323	2.06			
**5a**	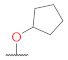	298	8.59	−5.33 ± 0.17	−48.02 ± 2.95	−143.27 ± 9.49
		313	3.36			
		323	1.92			
**5b**	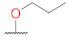	298	11.84	−6.12 ± 0.16	−31.55 ± 2.75	−85.50 ± 8.83
		313	6.00			
		323	4.45			

* Expressed as an integral an integral ratio of the tetrazole/azide tautomeric forms K_(eq)_ = [T]/[A].

## Supplementary Materials

The following supporting information can be downloaded at: https://www.mdpi.com/article/10.3390/molecules27227675/s1, Gibbs and Van’t plots, complete table of thermodynamic values calculated and ^1^H, ^13^C and ^31^P NMR spectra are available in the supporting information.

## Sample Availability

Samples of the compounds are not available.
