# Supplementary material for: SnAr Reactions of 2,4-Diazidopyrido[3,2-d]pyrimidine and Azide-Tetrazole Equilibrium Studies of the Obtained 5-Substituted Tetrazolo[1,5-a]pyrido[2,3-e]pyrimidines
Source: Molecules. 2022 Nov 8;27(22):7675. doi: 10.3390/molecules27227675 (PMC9698326; doi:10.3390/molecules27227675)

Supplementary material

S_NAr Reactions of 2,4-Diazidopyrido[3,2-*e*]pyrimidine and Azide-Tetrazole Equilibrium Studies of the Obtained 5-Substituted Tetrazolo[1,5-*a*]pyrido[2,3-*e*]pyrimidines

Kristaps Leškovskis[1], Anatoly Mishnev[2] and Irina Novosjolova[1,*], Māris Turks[1,*]

[1]Institute of Technology of Organic Chemistry, Faculty of Materials Science and Applied Chemistry, Riga Technical University, P. Valdena str. 3, Riga, LV 1048, Latvia

[2]Latvian Institute of Organic Synthesis, Aizkraukles str. 21, Riga, LV-1006, Latvia

*****Correspondence: Irina.Novosjolova@rtu.lv (I.N.); Maris.Turks@rtu.lv (M.T.)

Contents

Gibbs and van’t Hoff plots 3

Gibbs and van’ t Hoff plot derived thermodynamic values of tautomerization for compounds **3-5** 7

^{1}H NMR spectra of tetrazoles **3-5** in $CDCl_3$ at variable temperatures... 8

^{1}H, ^{13}C and ^{31}P NMR for obtained compounds 15

Gibbs and van’t Hoff plots

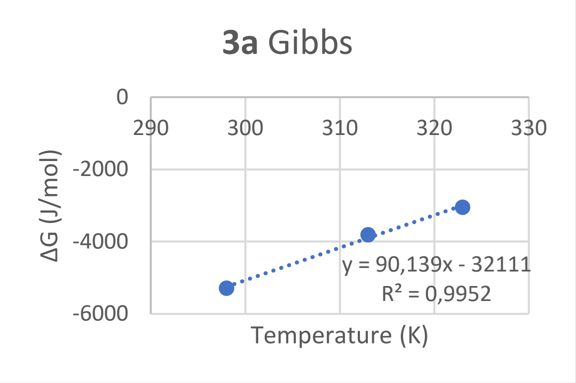

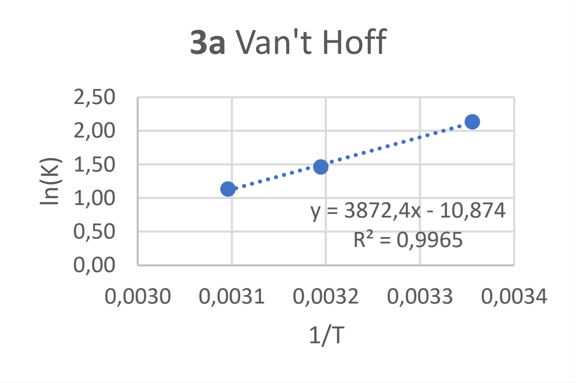

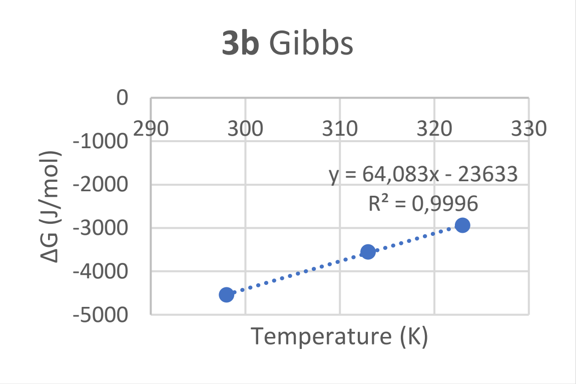

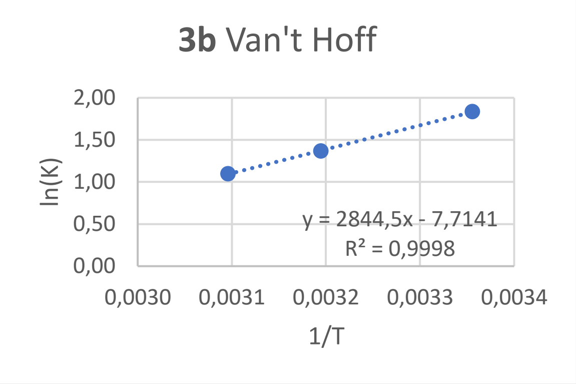

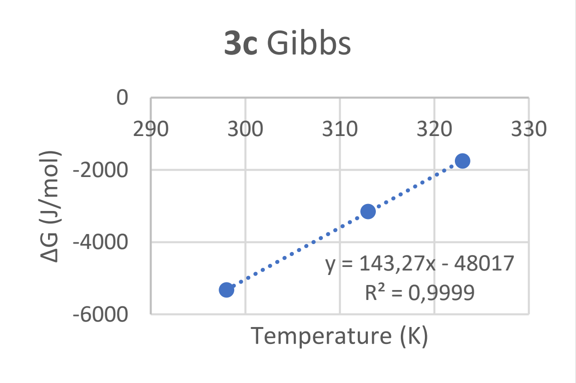

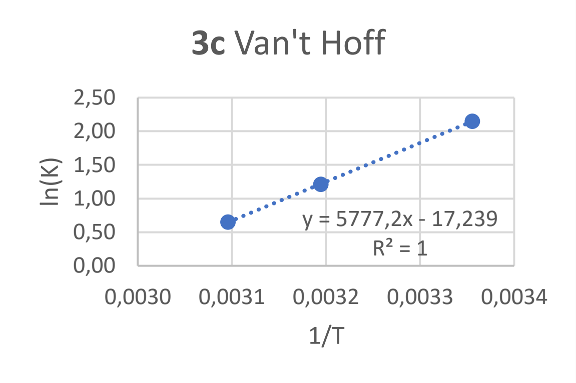

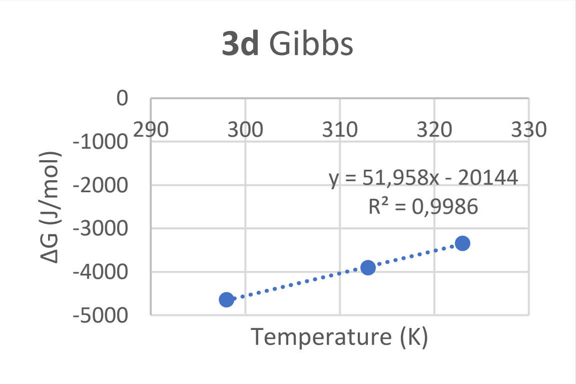

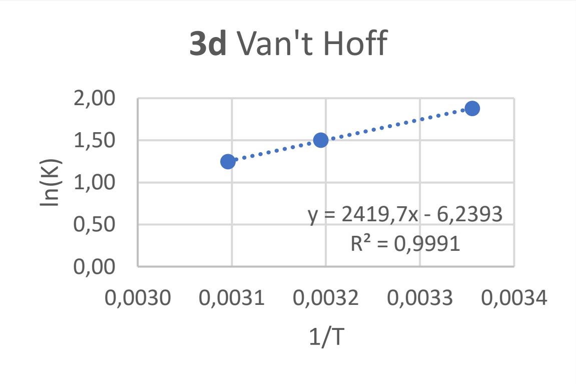

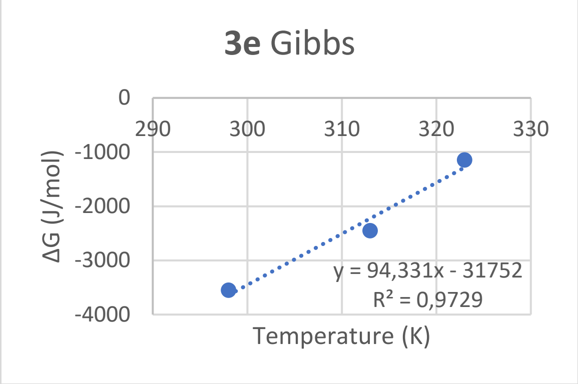
3e Gibbs
ΔG (J/mol)
0
-1000
-2000
-3000
-4000
290
300
310
320
330
y = 94,331x - 31752
R² = 0,9729
Temperature (K)

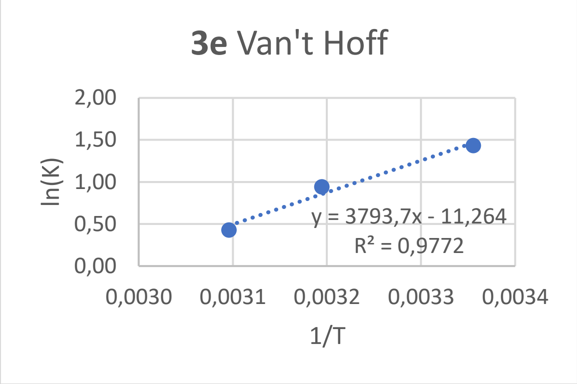
3e Van't Hoff
ln(K)
2,00
1,50
1,00
0,50
0,00
0,0030
0,0031
0,0032
0,0033
0,0034
y = 3793,7x - 11,264
R² = 0,9772
1/T

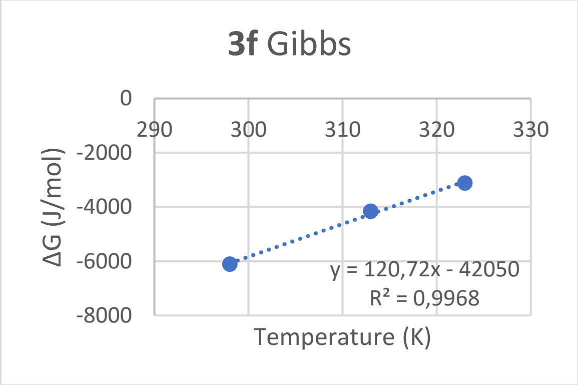
3f Gibbs
ΔG (J/mol)
0
-2000
-4000
-6000
-8000
290
300
310
320
330
y = 120,72x - 42050
R² = 0,9968
Temperature (K)

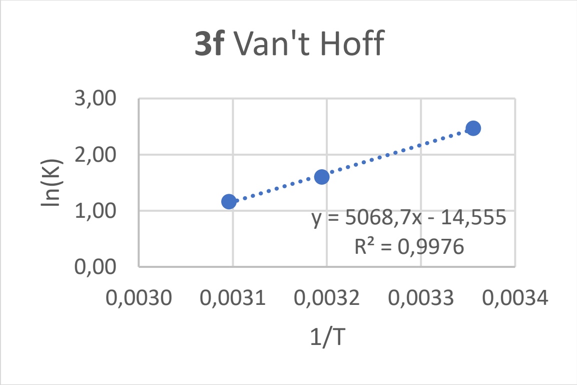
3f Van't Hoff
ln(K)
3,00
2,00
1,00
0,00
0,0030
0,0031
0,0032
0,0033
0,0034
y = 5068,7x - 14,555
R² = 0,9976
1/T

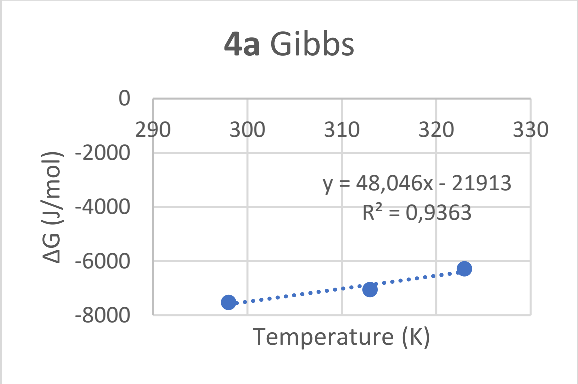
4a Gibbs
ΔG (J/mol)
0
-2000
-4000
-6000
-8000
290
300
310
320
330
y = 48,046x - 21913
R² = 0,9363
Temperature (K)

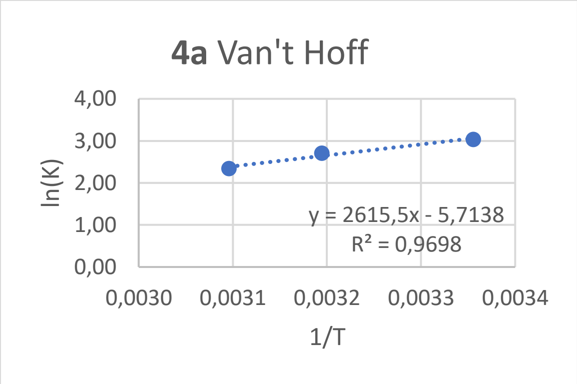
4a Van't Hoff
ln(K)
4,00
3,00
2,00
1,00
0,00
0,0030
0,0031
0,0032
0,0033
0,0034
y = 2615,5x - 5,7138
R² = 0,9698
1/T

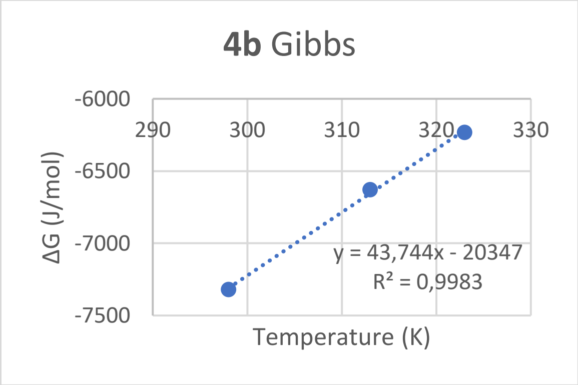
4b Gibbs
ΔG (J/mol)
-6000
-6500
-7000
-7500
290
300
310
320
330
y = 43,744x - 20347
R² = 0,9983
Temperature (K)

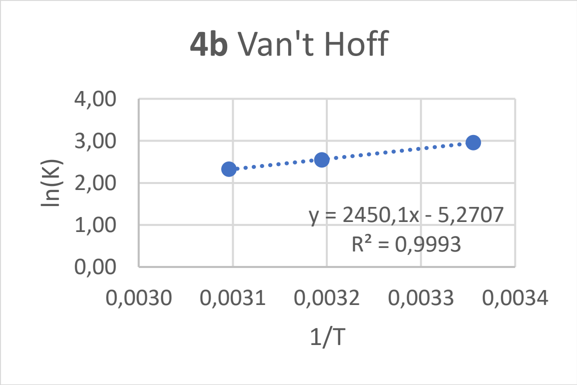
4b Van't Hoff
ln(K)
4,00
3,00
2,00
1,00
0,00
0,0030
0,0031
0,0032
0,0033
0,0034
y = 2450,1x - 5,2707
R² = 0,9993
1/T

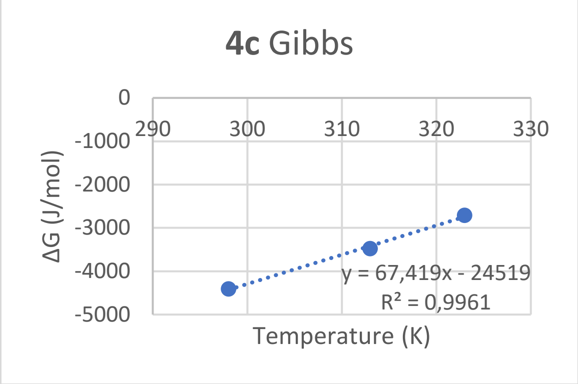
4c Gibbs
0
-1000
-2000
-3000
-4000
-5000
290
300
310
320
330
ΔG (J/mol)
Temperature (K)
y = 67,419x - 24519
R² = 0,9961

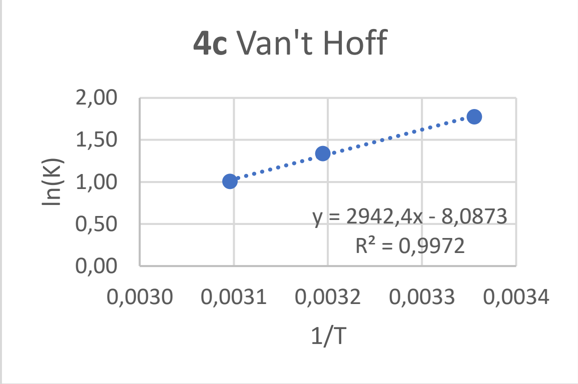
4c Van't Hoff
2,00
1,50
1,00
0,50
0,00
0,0030
0,0031
0,0032
0,0033
0,0034
ln(K)
1/T
y = 2942,4x - 8,0873
R² = 0,9972

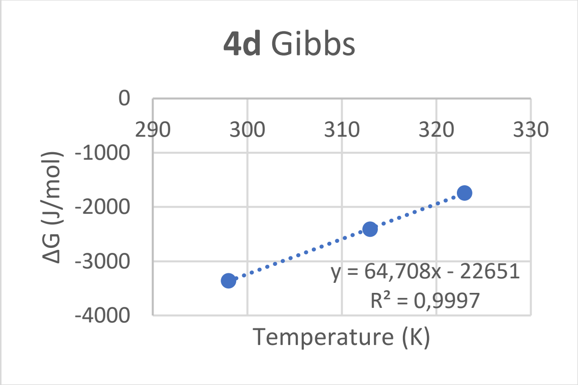
4d Gibbs
0
-1000
-2000
-3000
-4000
290
300
310
320
330
ΔG (J/mol)
Temperature (K)
y = 64,708x - 22651
R² = 0,9997

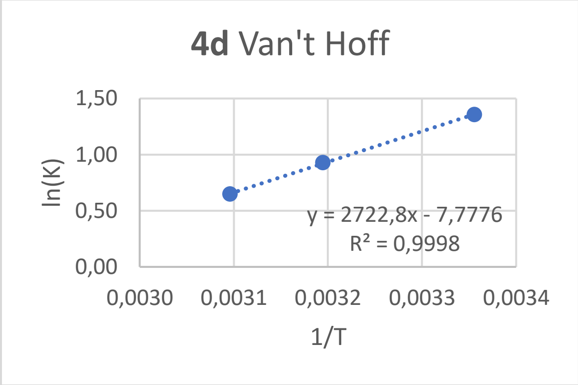
4d Van't Hoff
1,50
1,00
0,50
0,00
0,0030
0,0031
0,0032
0,0033
0,0034
ln(K)
1/T
y = 2722,8x - 7,7776
R² = 0,9998

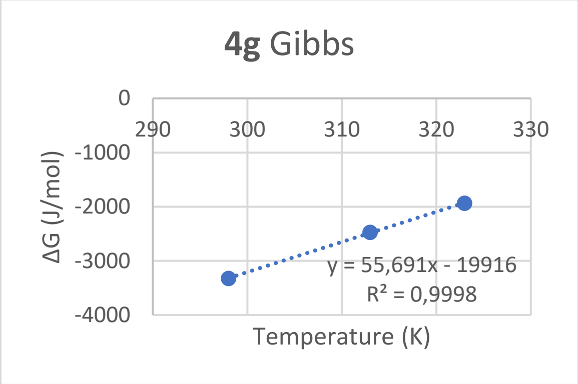
4g Gibbs
0
-1000
-2000
-3000
-4000
290
300
310
320
330
ΔG (J/mol)
Temperature (K)
y = 55,691x - 19916
R² = 0,9998

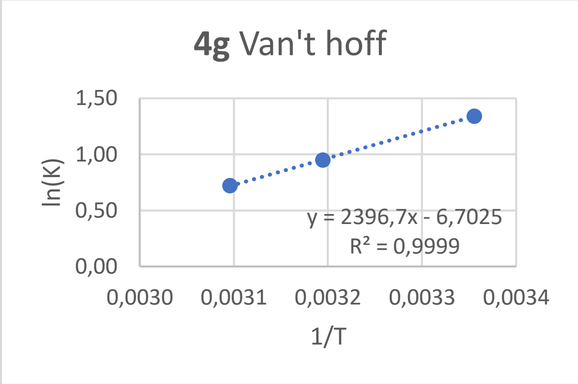
4g Van't hoff
1,50
1,00
0,50
0,00
0,0030
0,0031
0,0032
0,0033
0,0034
ln(K)
1/T
y = 2396,7x - 6,7025
R² = 0,9999

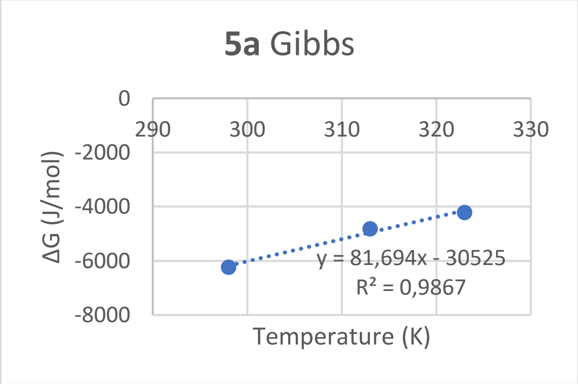
5a Gibbs
0
-2000
-4000
-6000
-8000
290
300
310
320
330
ΔG (J/mol)
Temperature (K)
y = 81,694x - 30525
R² = 0,9867

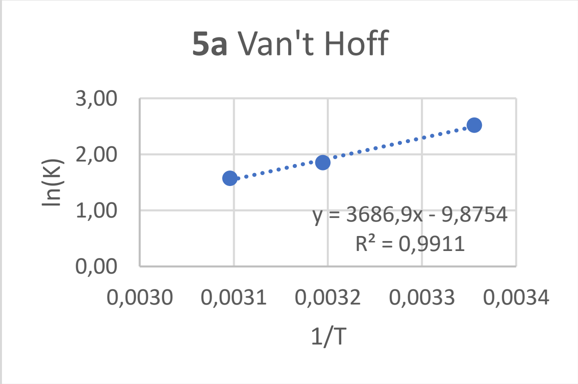
5a Van't Hoff
3,00
2,00
1,00
0,00
0,0030
0,0031
0,0032
0,0033
0,0034
ln(K)
1/T
y = 3686,9x - 9,8754
R² = 0,9911

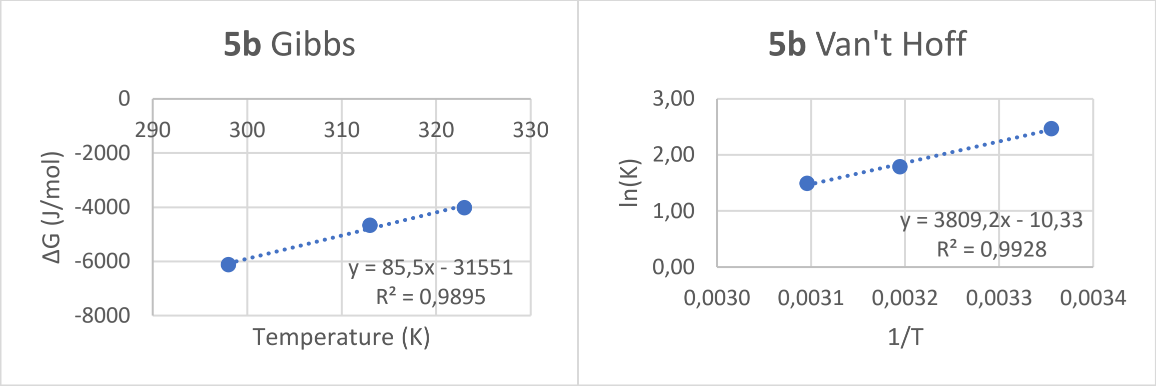
5b Gibbs
ΔG (J/mol)
Temperature (K)
0
-2000
-4000
-6000
-8000
290
300
310
320
330
y = 85,5x - 31551
R² = 0,9895
5b Van't Hoff
ln(K)
1/T
3,00
2,00
1,00
0,00
0,0030
0,0031
0,0032
0,0033
0,0034
y = 3809,2x - 10,33
R² = 0,9928

Gibbs and van' t Hoff plot derived thermodynamic values of tautomerization for compounds 3-5

	T (K)	Gibbs plot values			Van't Hoff plot values		
		ΔG (kJ/mol)	ΔH_{298} (kJ/mol)	ΔS_{298} (J/mol·K)	ΔG (kJ/mol)	ΔH_{298} (kJ/mol)	ΔS_{298} (J/mol·K)
	298	-5.29			-5.25		
**3a**	313	-3.81	-32.11	-90.14	-3.90	-32.20	-90.41
	323	-3.05			-2.99		
	298	-4.54			-4.54		
**3b**	313	-3.56	-23.63	-64.08	-3.58	-23.65	-64.14
	323	-2.95			-2.93		
	298	-5.33			-5.32		
**3c**	313	-3.16	-48.02	-143.27	-3.17	-48.03	-143.33
	323	-1.75			-1.74		
	298	-4.65			-4.66		
**3d**	313	-3.91	-20.14	-51.96	-3.88	-20.12	-51.87
	323	-3.34			-3.36		
	298	-3.55			-3.63		
**3e**	313	-2.45	-31.75	-94.33	-2.23	-31.54	-93.65
	323	-1.15			-1.29		
	298	-6.12			-6.08		
**3f**	313	-4.17	-42.05	-120.72	-4.27	-42.14	-121.01
	323	-3.12			-3.06		
	298	-7.52			-7.59		
**4a**	313	-7.06	-21.91	-48.05	-6.88	-21.75	-47.50
	323	-6.29			-6.40		
	298	-7.32			-7.31		
**4b**	313	-6.63	-20.35	-43.74	-6.65	-20.37	-43.82
	323	-6.23			-6.22		
	298	-4.40			-4.43		
**4c**	313	-3.48	-24.52	-67.42	-3.42	-24.46	-67.24
	323	-2.71			-2.75		
	298	-3.36			-3.37		
**4d**	313	-2.41	-22.65	-64.71	-2.40	-22.64	-64.66
	323	-1.74			-1.75		
	298	-3.33			-3.32		
**4g**	313	-2.47	-19.92	-55.69	-2.49	-19.93	-55.72
	323	-1.94			-1.93		
	298	-6.24			-6.19		
**5a**	313	-4.82	-30.53	-81.69	-4.95	-30.65	-82.10
	323	-4.22			-4.13		
	298	-6.12			-6.08		
**5b**	313	-4.66	-31.55	-85.50	-4.79	-31.67	-85.88
	323	-4.01			-3.93		

^{1}H NMR spectra of tetrazoles 3-5 in $CDCl_3$ at variable temperatures

5-(Butylthio)pyrido[2,3-*e*]tetrazolo[1,5-*a*]pyrimidine (**3a**):

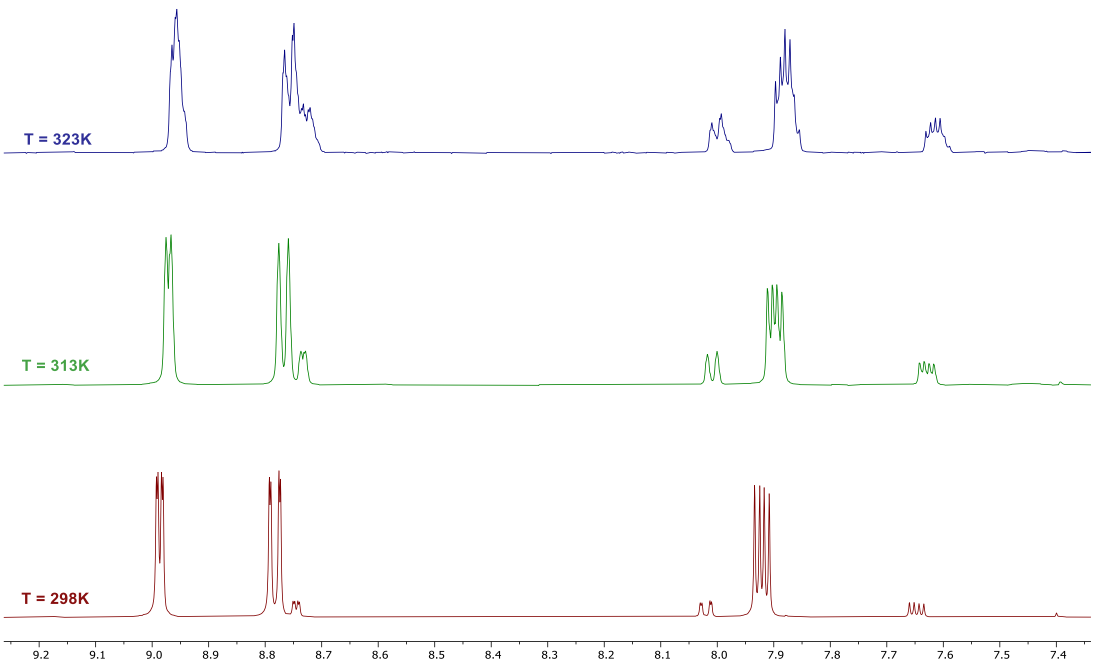

5-(Phenethylthio)pyrido[2,3-*e*]tetrazolo[1,5-*a*]pyrimidine (**3b**):

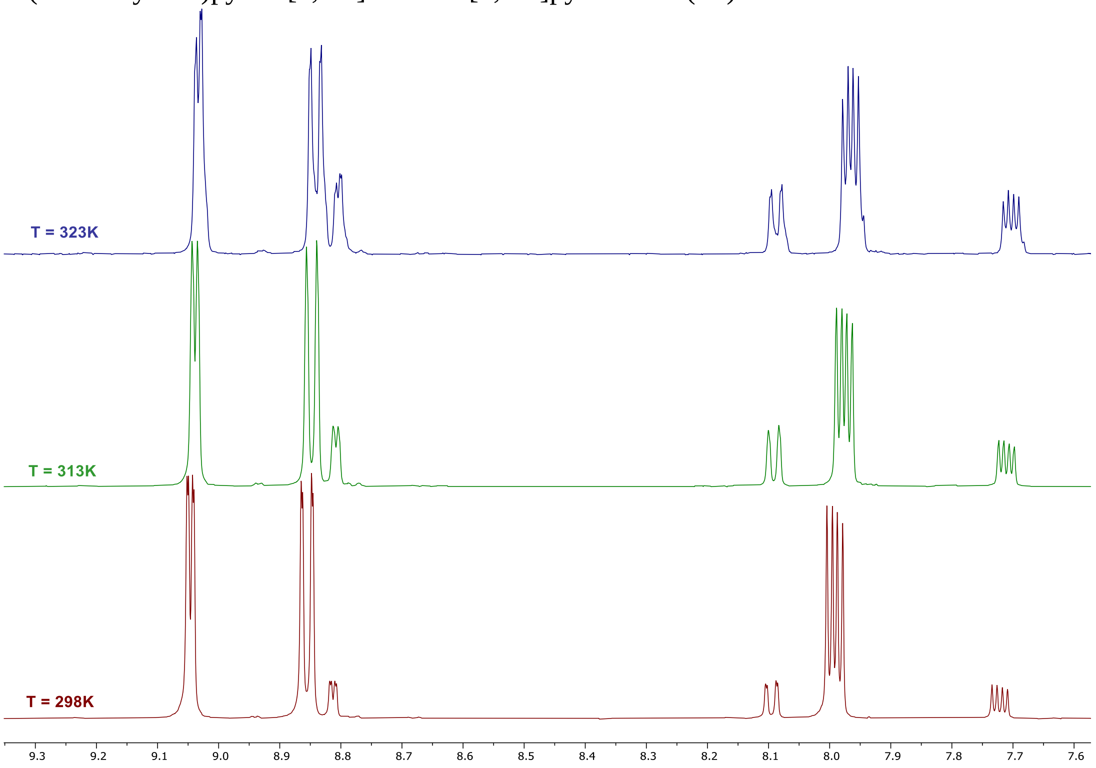

5-(*p*-Tolylthio)pyrido[2,3-*e*]tetrazolo[1,5-*a*]pyrimidine (**3c**):

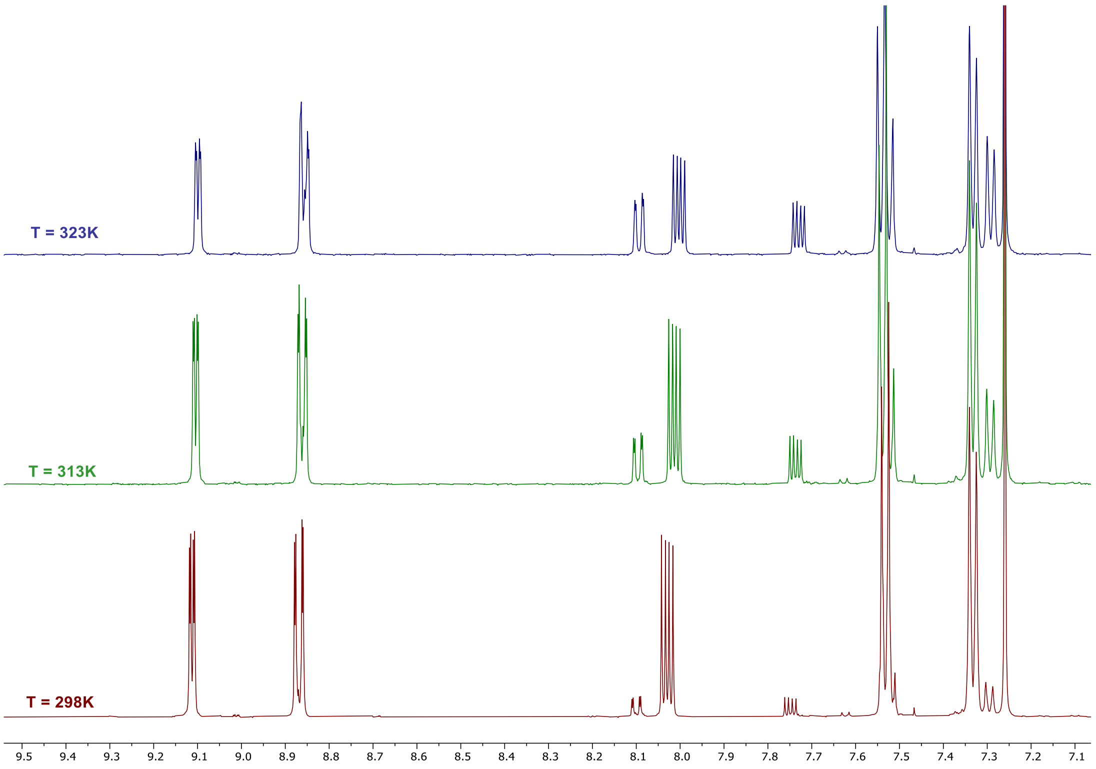

5-(Isopropylthio)pyrido[2,3-*e*]tetrazolo[1,5-*a*]pyrimidine (**3d**):

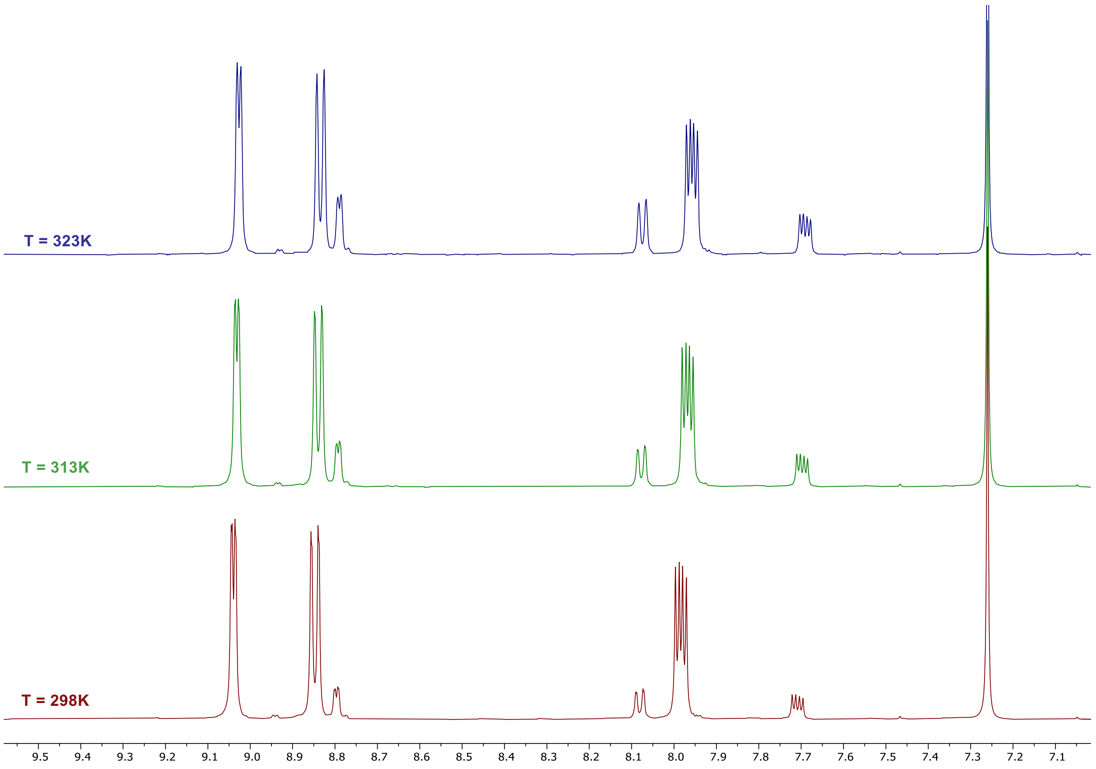

5-(Phenylthio)pyrido[2,3-*e*]tetrazolo[1,5-*a*]pyrimidine (**3e**):

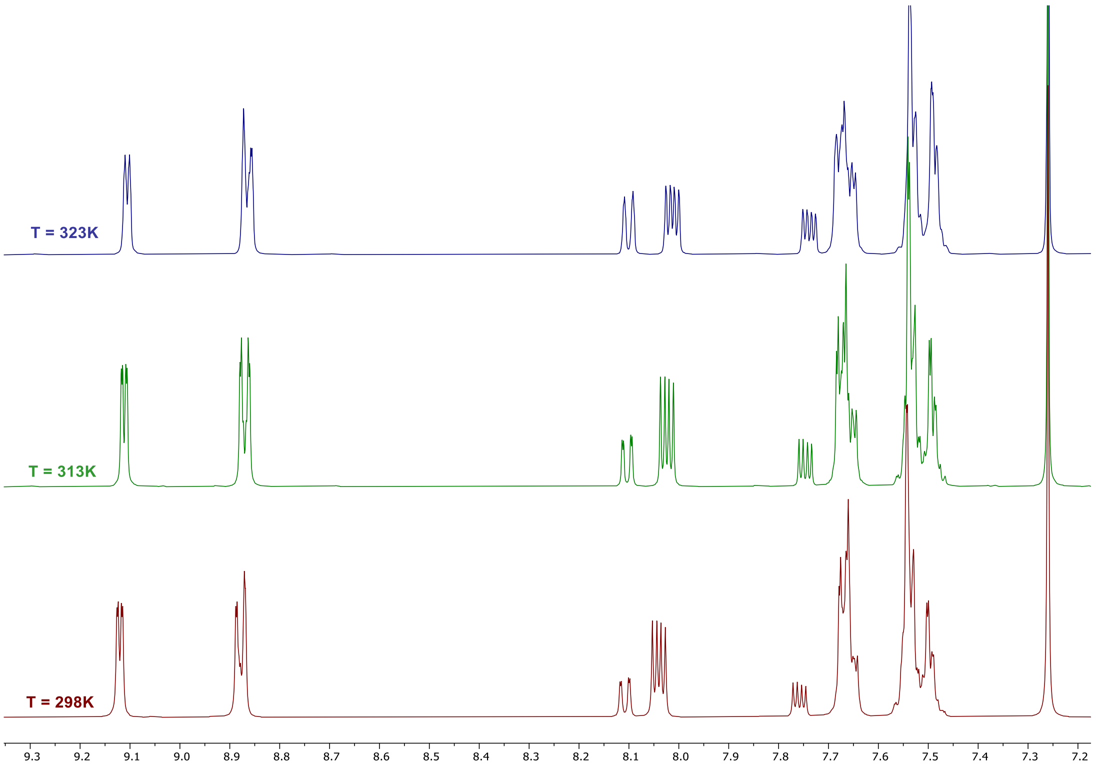

5-(Cyclohexylthio)pyrido[2,3-*e*]tetrazolo[1,5-*a*]pyrimidine (**3f**):

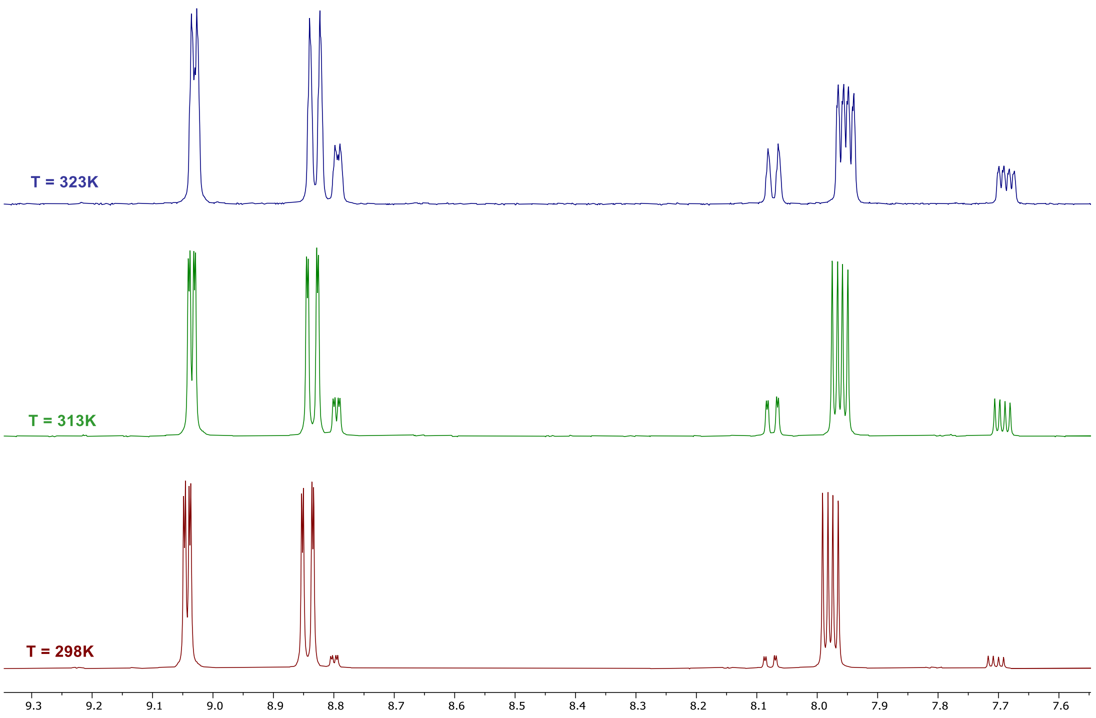

N-(4-Methoxybenzyl)pyrido[2,3-*e*]tetrazolo[1,5-*a*]pyrimidin-5-amine (**4a**):

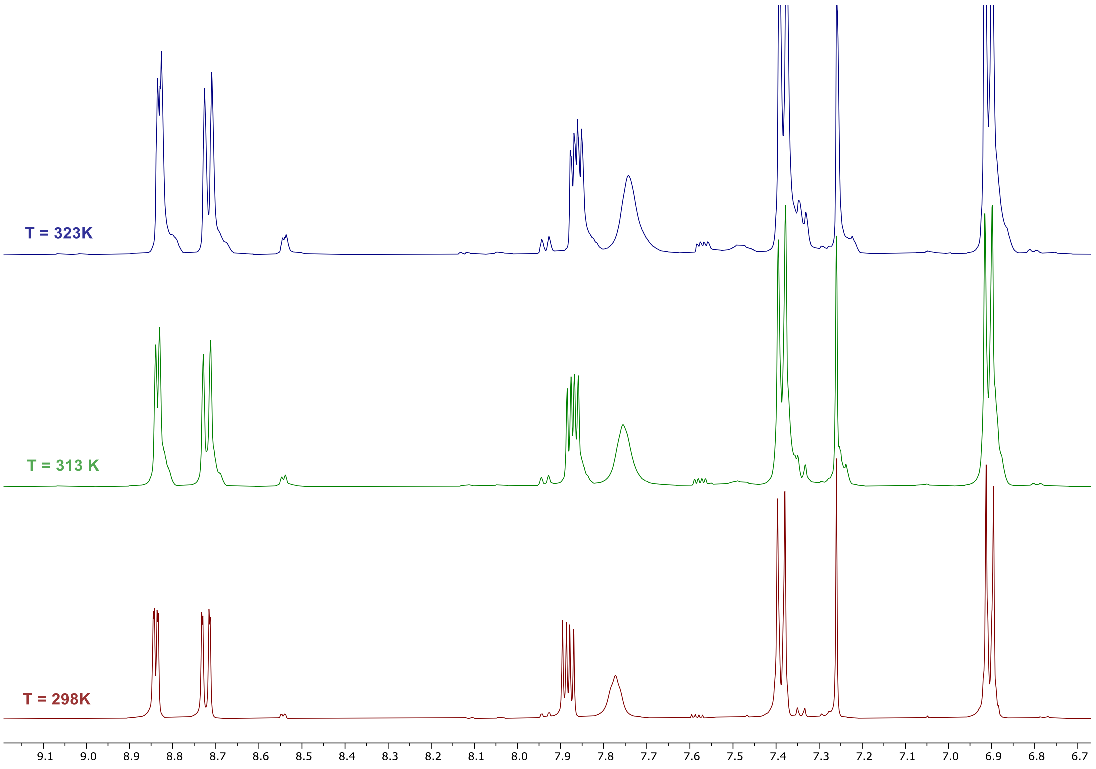

N-Hexylpyrido[2,3-*e*]tetrazolo[1,5-*a*]pyrimidin-5-amine (**4b**):

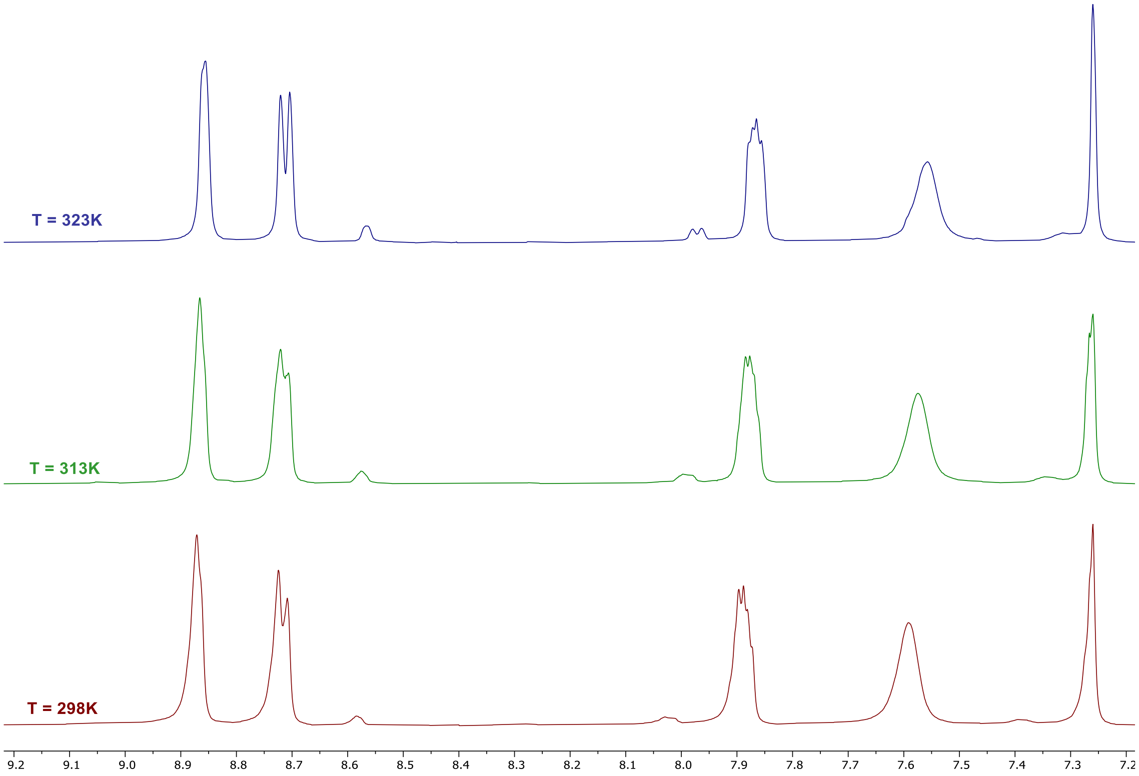

5-(Piperidin-1-yl)pyrido[2,3-*e*]tetrazolo[1,5-*a*]pyrimidine (**4c**):

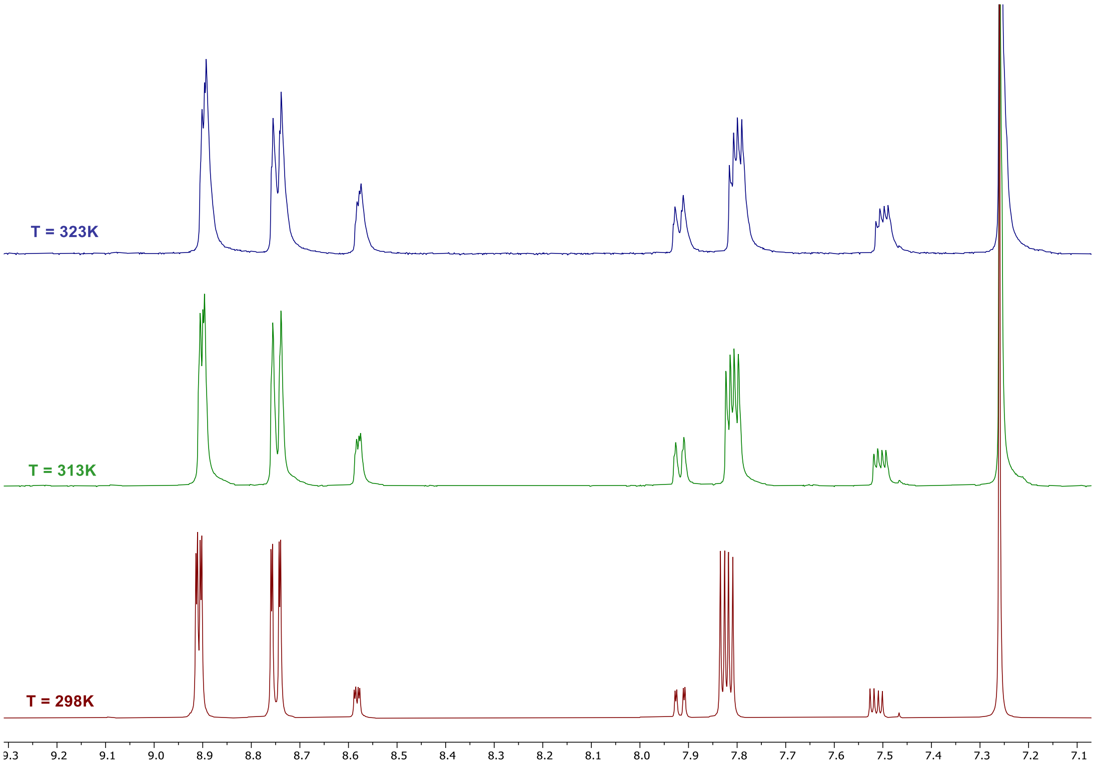

4-(Pyrido[2,3-*e*]tetrazolo[1,5-*a*]pyrimidin-5-yl)morpholine (**4d**):

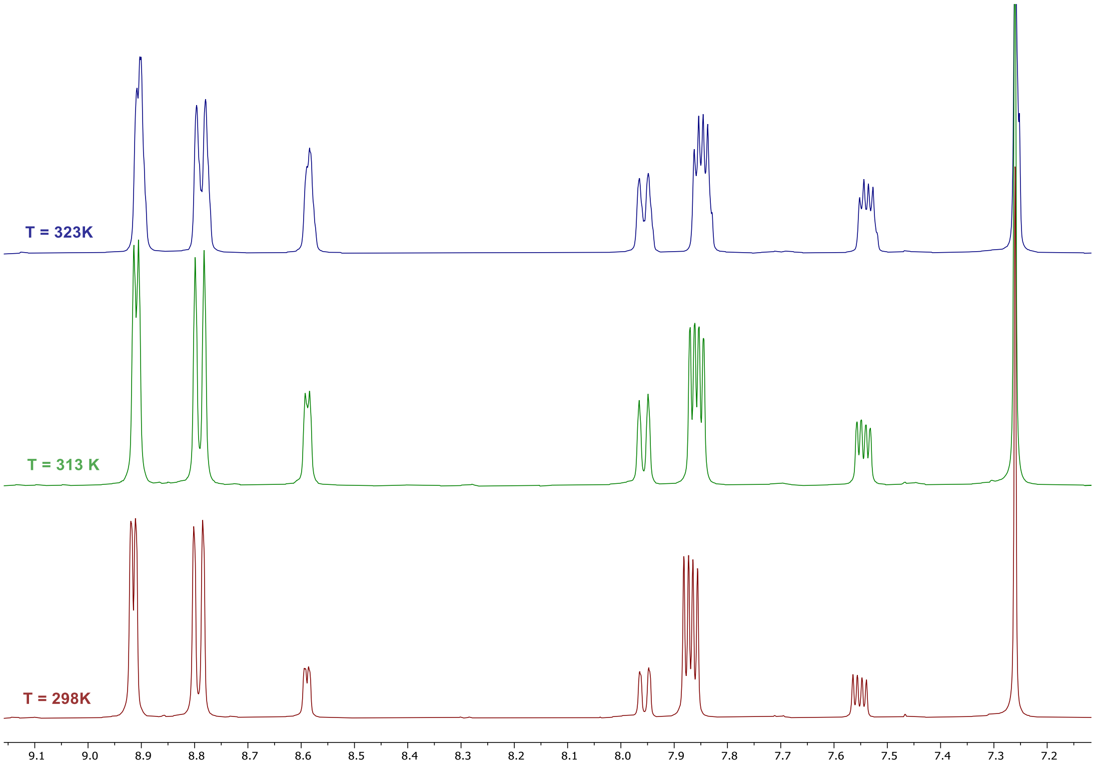

5-(4-Methylpiperazin-1-yl)pyrido[2,3-*e*]tetrazolo[1,5-*a*]pyrimidine (**4g**):

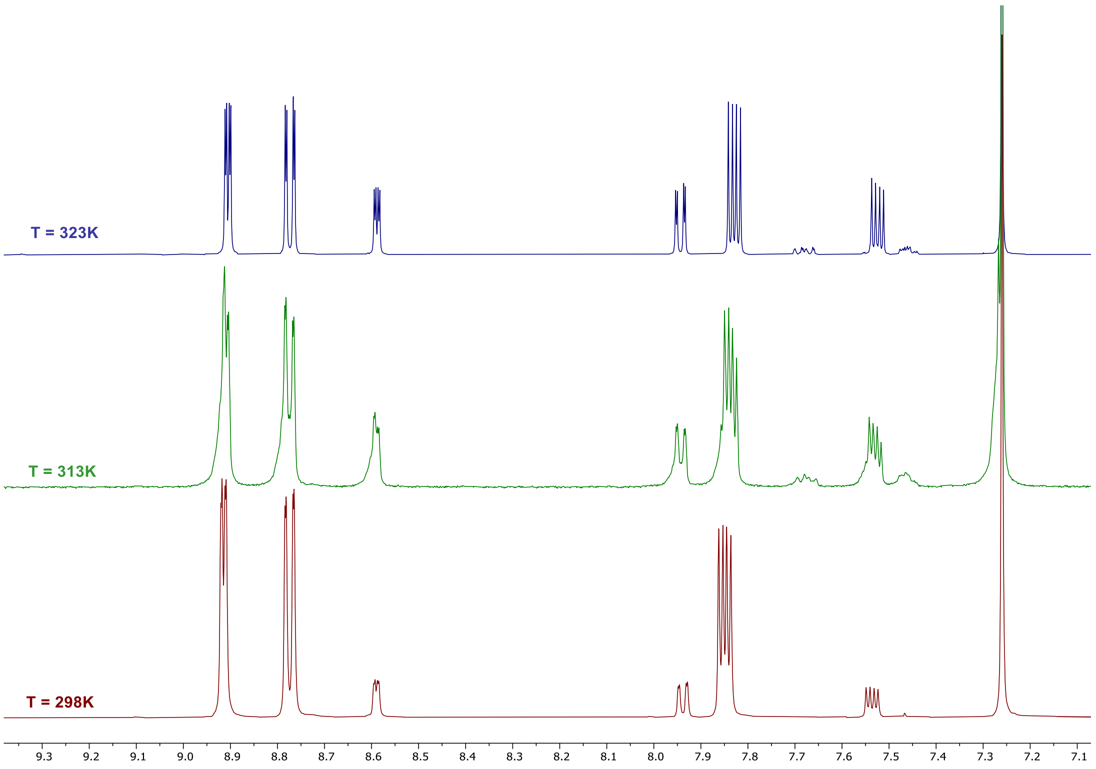

5-(Cyclopentyloxy)pyrido[2,3-*e*]tetrazolo[1,5-*a*]pyrimidine (**5a**):

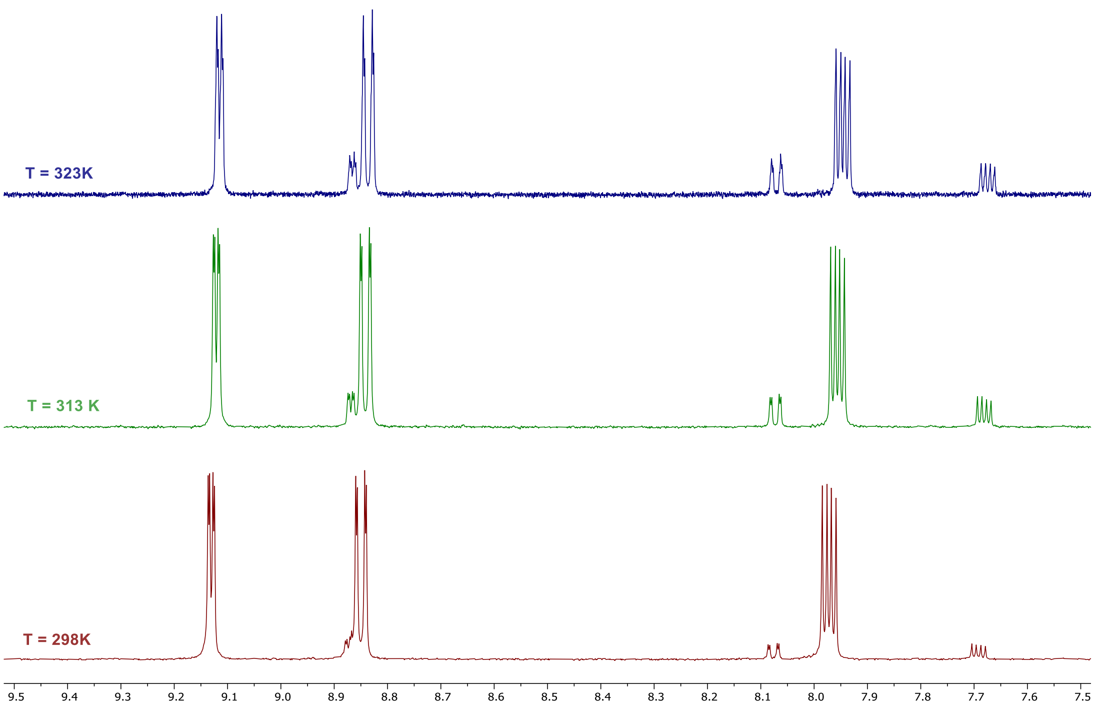

5-Propoxypyrido[2,3-*e*]tetrazolo[1,5-*a*]pyrimidine (**5b**):

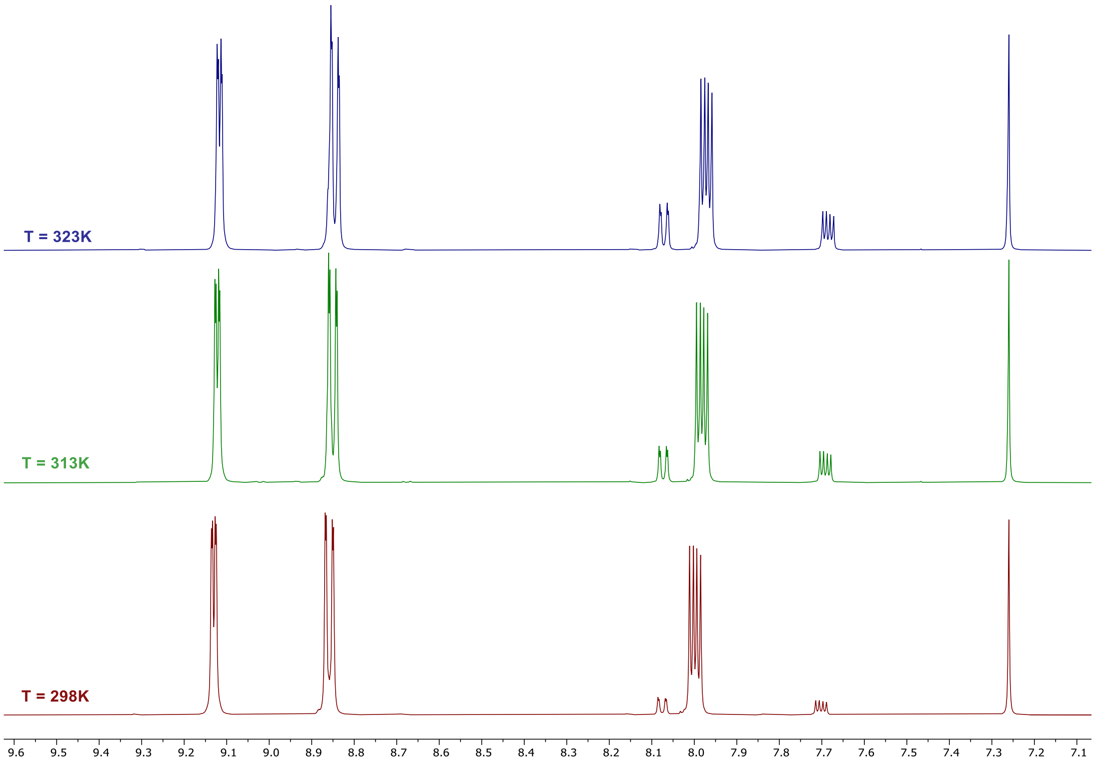

^{1}H, ^{13}C and ^{31}P NMR for obtained compounds

2,4-Diazidopyrido[3,2-*d*]pyrimidine (**2**):

^{1}H NMR (500 MHz, CF_3COOD)

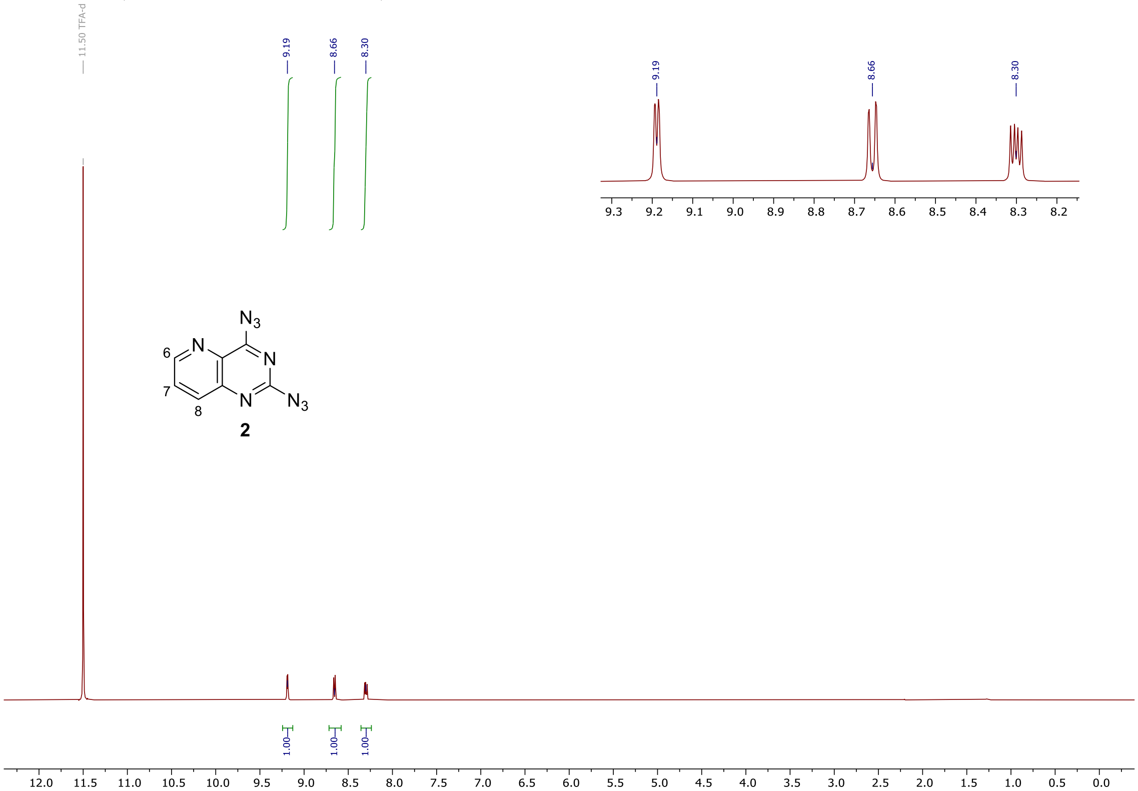

^{13}C NMR (125 MHz, CF_3COOD)

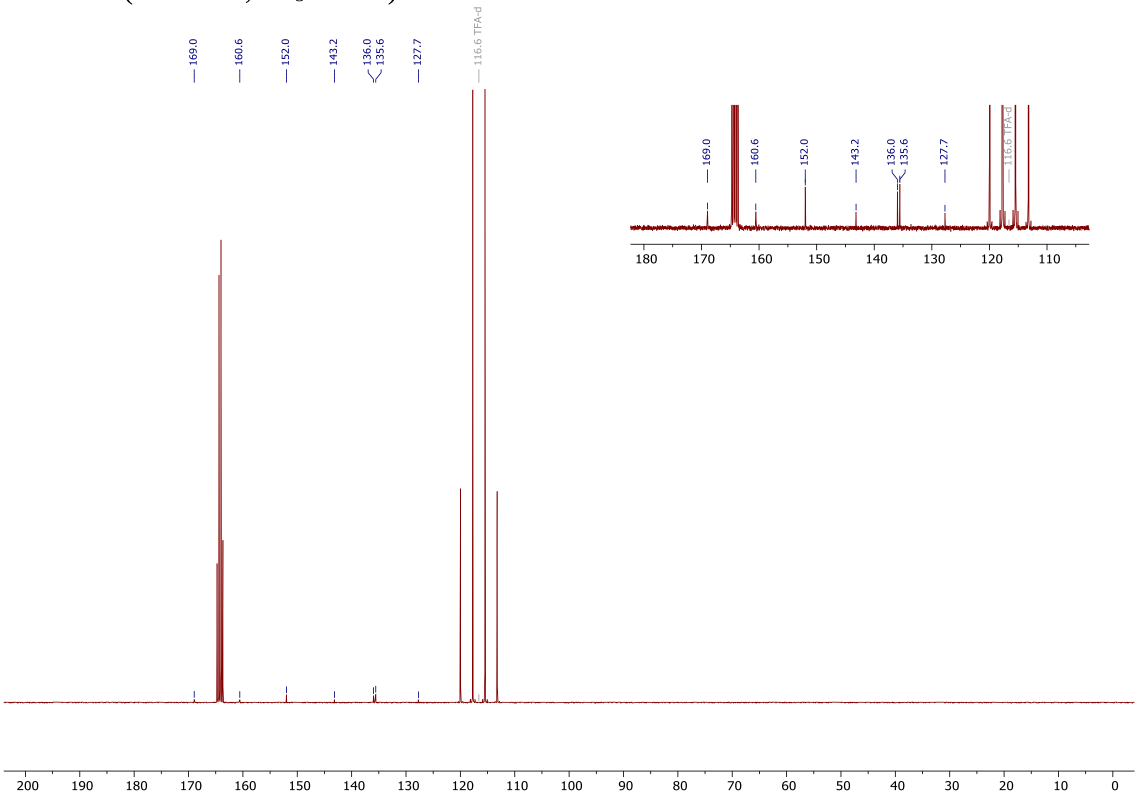

^{1}H NMR (500 MHz, DMSO-d_6)

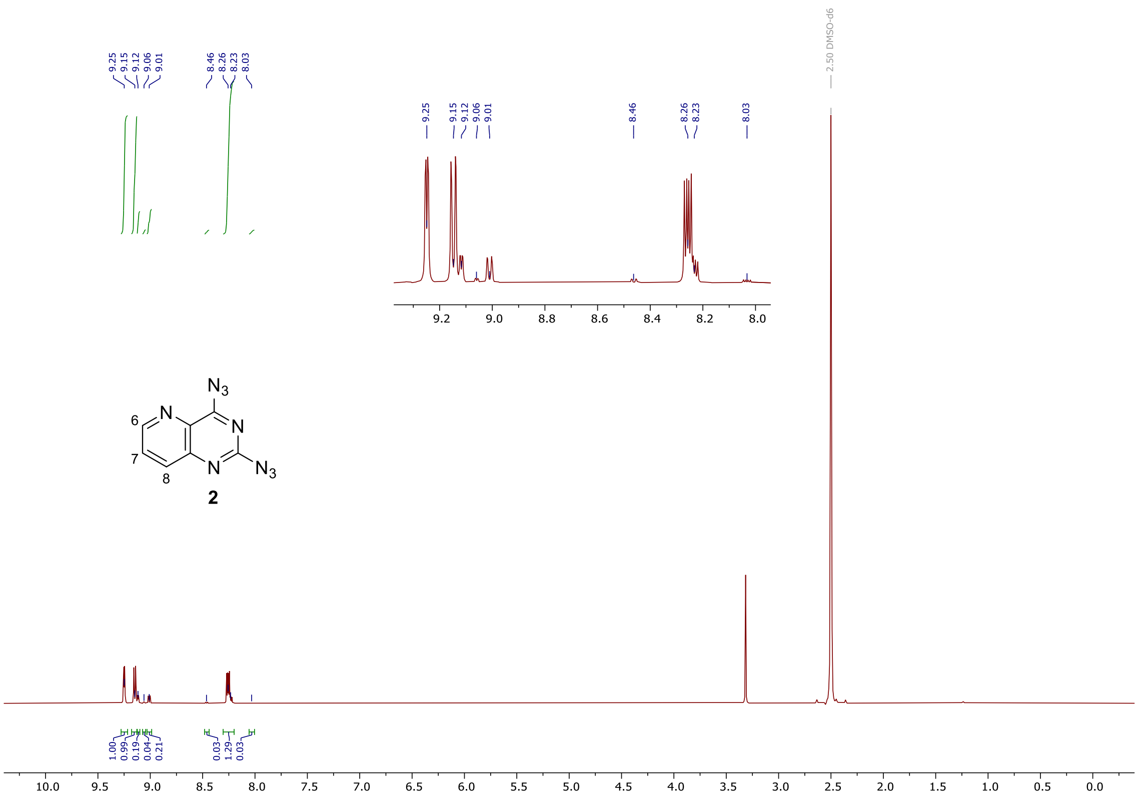

^{1}H NMR (500 MHz, $CDCl_3$)

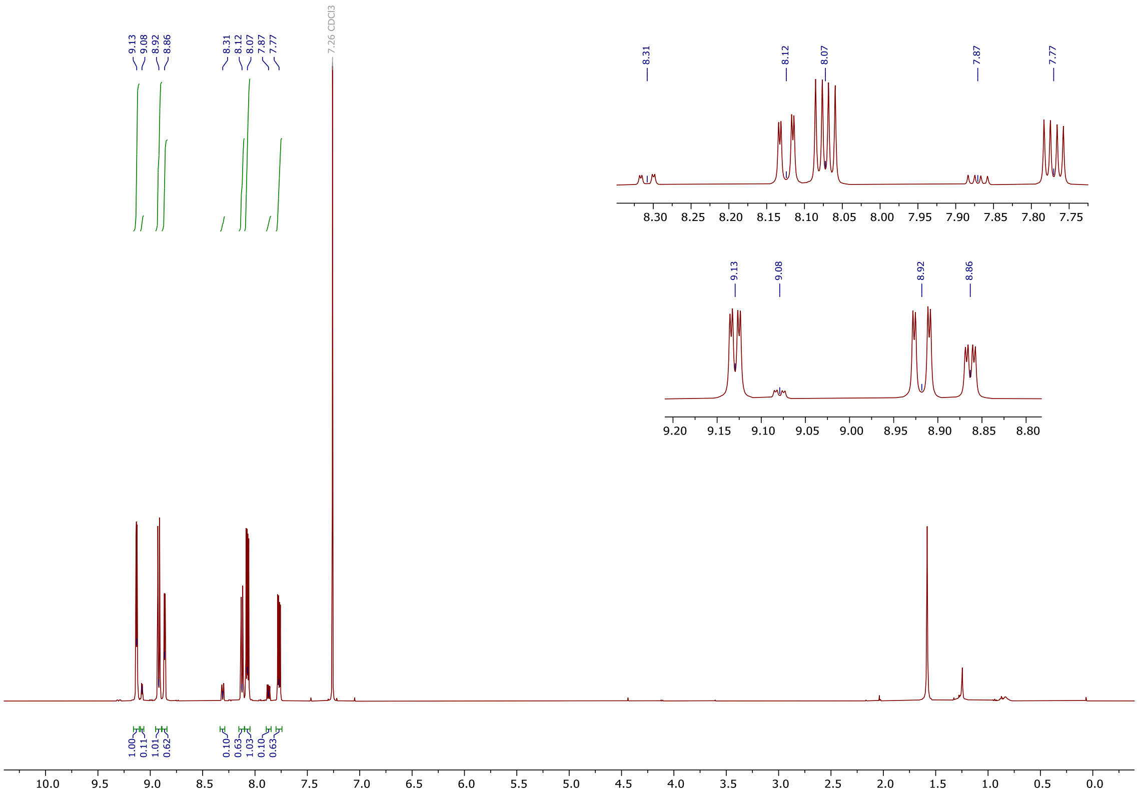

5-(Butylthio)pyrido[2,3-*e*]tetrazolo[1,5-*a*]pyrimidine (**3a**):

^{1}H NMR (500 MHz, DMSO-d_6)

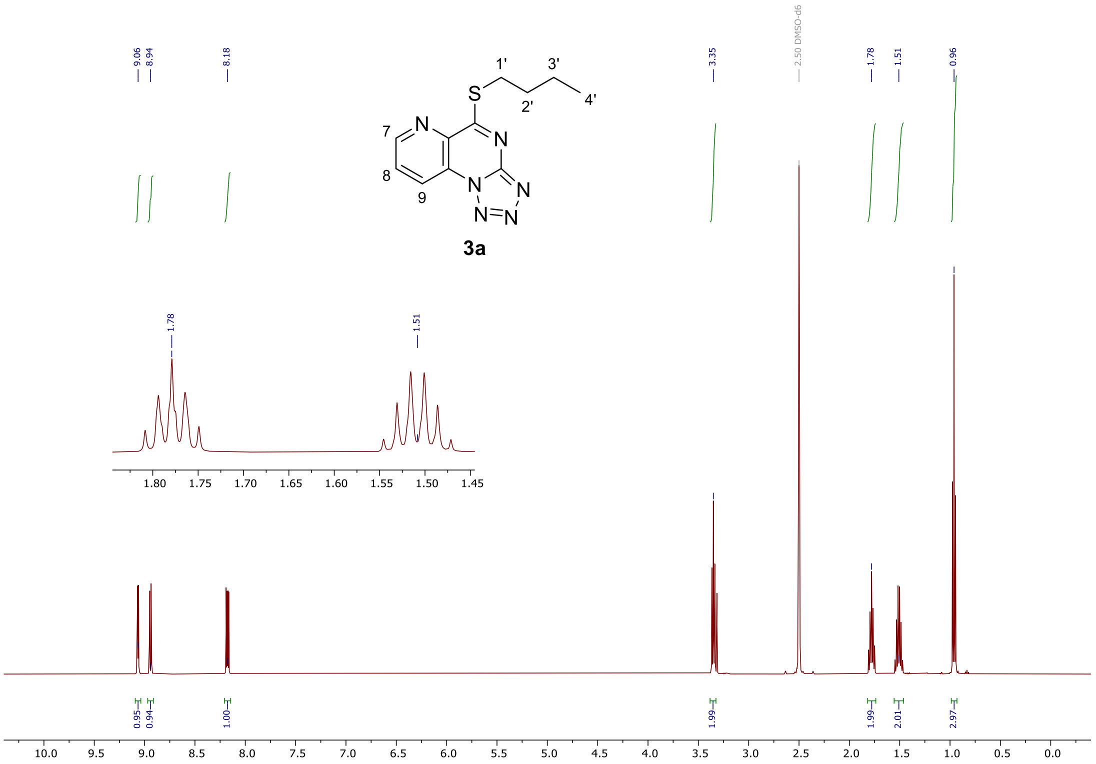

^{13}C NMR (125 MHz, DMSO-d_6)

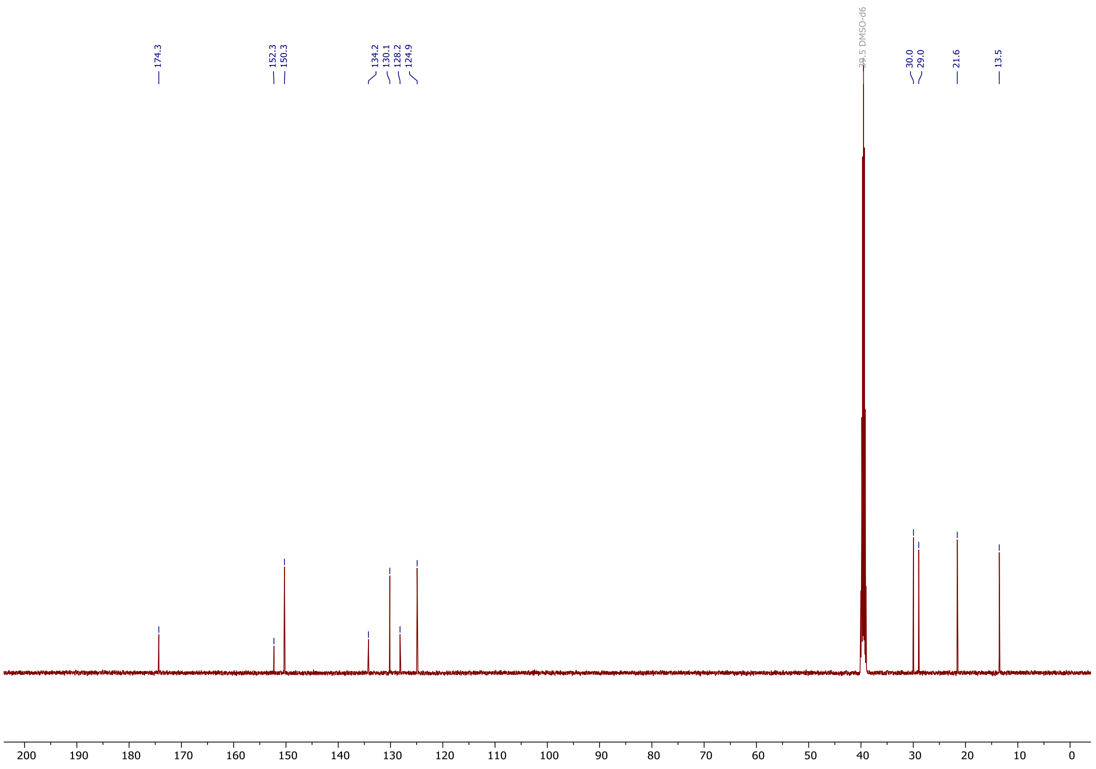

5-(Butylthio)pyrido[2,3-*e*]tetrazolo[1,5-*a*]pyrimidine (**3a**) and 2-azido-4-(butylthio)pyrido[3,2-*d*]pyrimidine (**3aA**):

^{1}H NMR (500 MHz, $CDCl_3$)

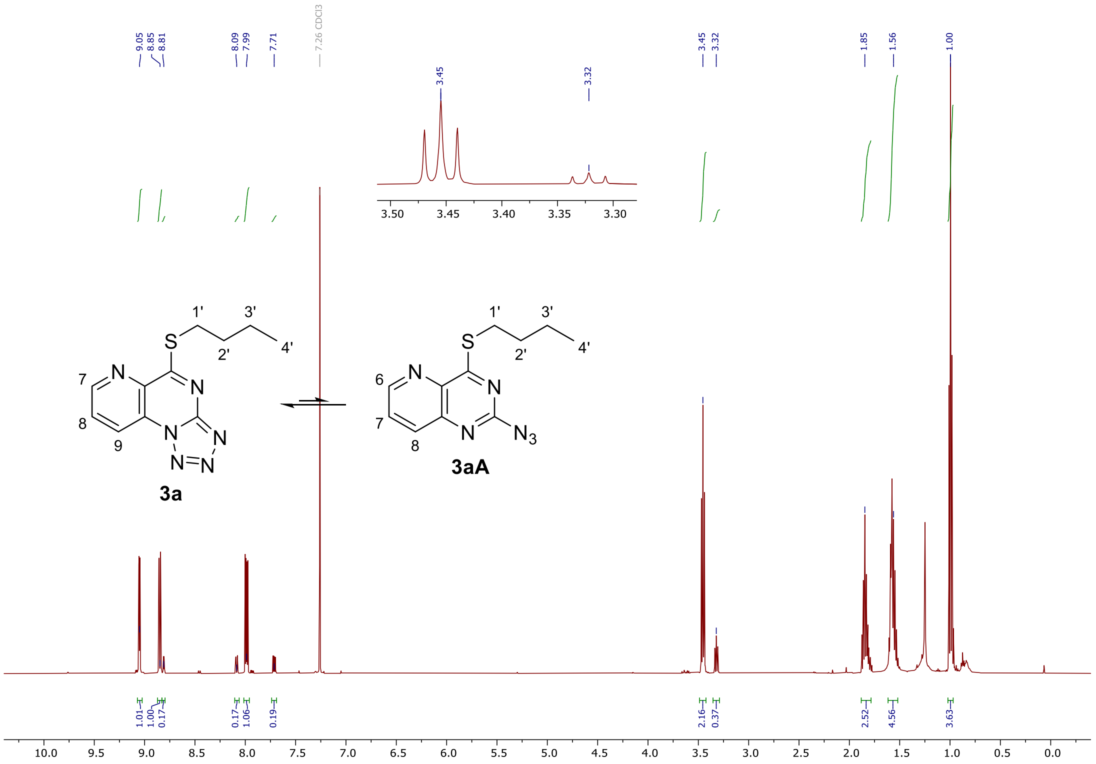

5-(Phenethylthio)pyrido[2,3-*e*]tetrazolo[1,5-*a*]pyrimidine (**3b**):

^{1}H NMR (500 MHz, DMSO-d_6)

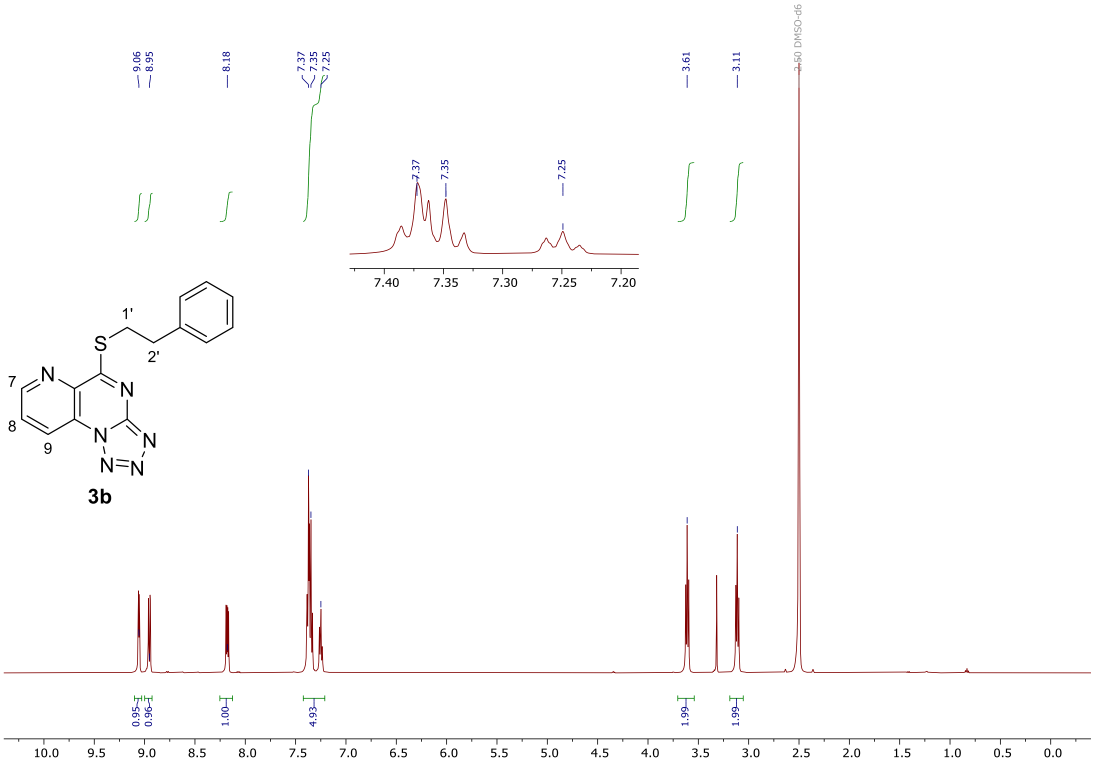

^{13}C NMR (125 MHz, DMSO-d_6)

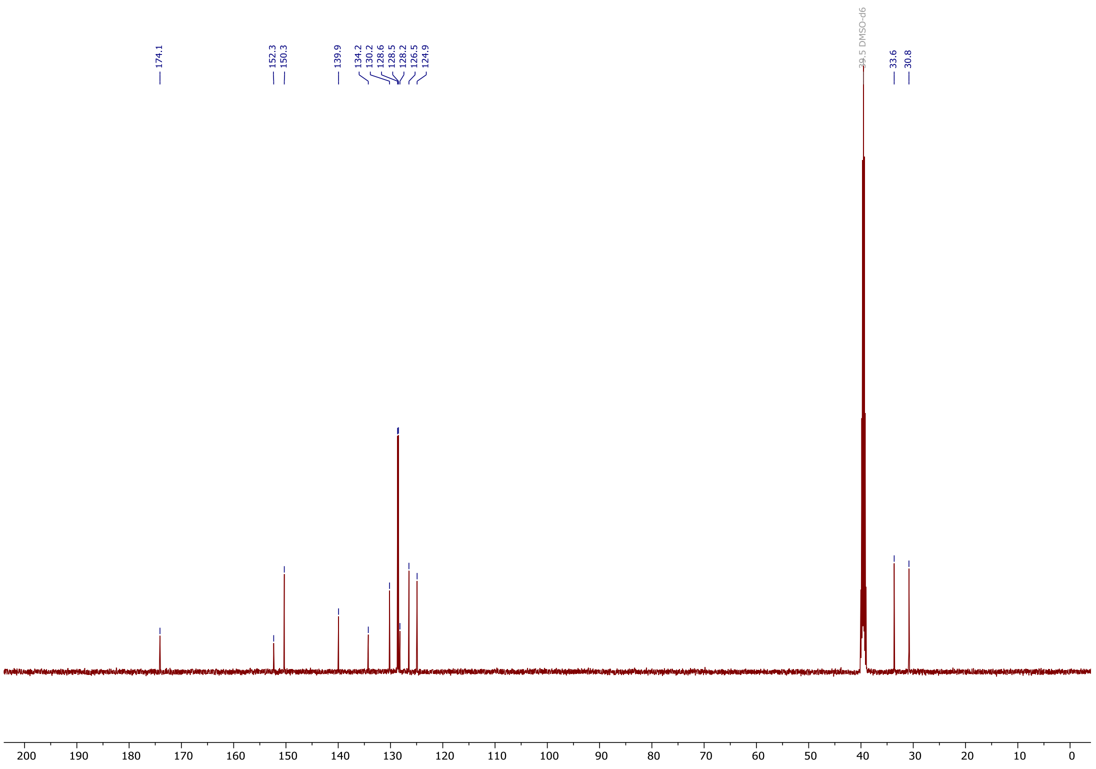

5-(Phenethylthio)pyrido[2,3-*e*]tetrazolo[1,5-*a*]pyrimidine (**3b**) and 2-azido-4-(phenethylthio)pyrido[3,2-*d*]pyrimidine (**3bA**):

^{1}H NMR (500 MHz, $CDCl_3$)

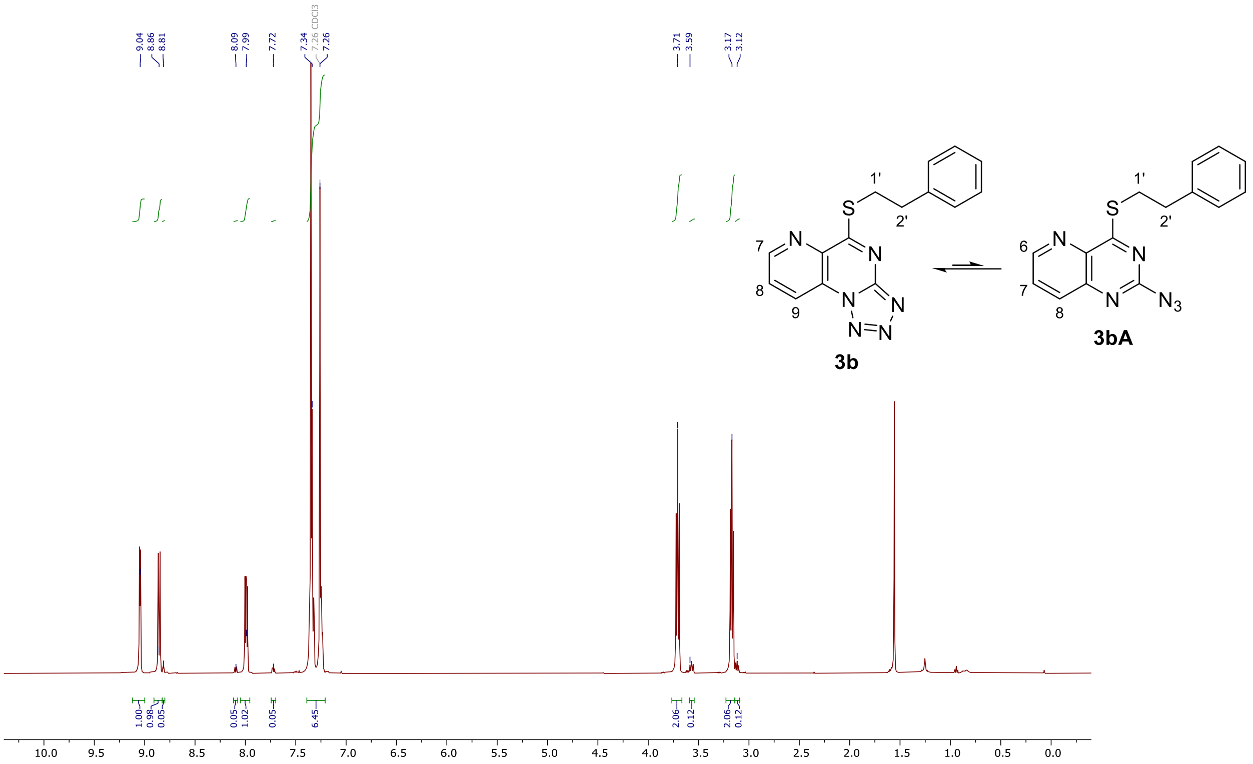

5-(*p*-Tolylthio)pyrido[2,3-*e*]tetrazolo[1,5-*a*]pyrimidine (**3c**):

^{1}H NMR (500 MHz, DMSO-d_6)

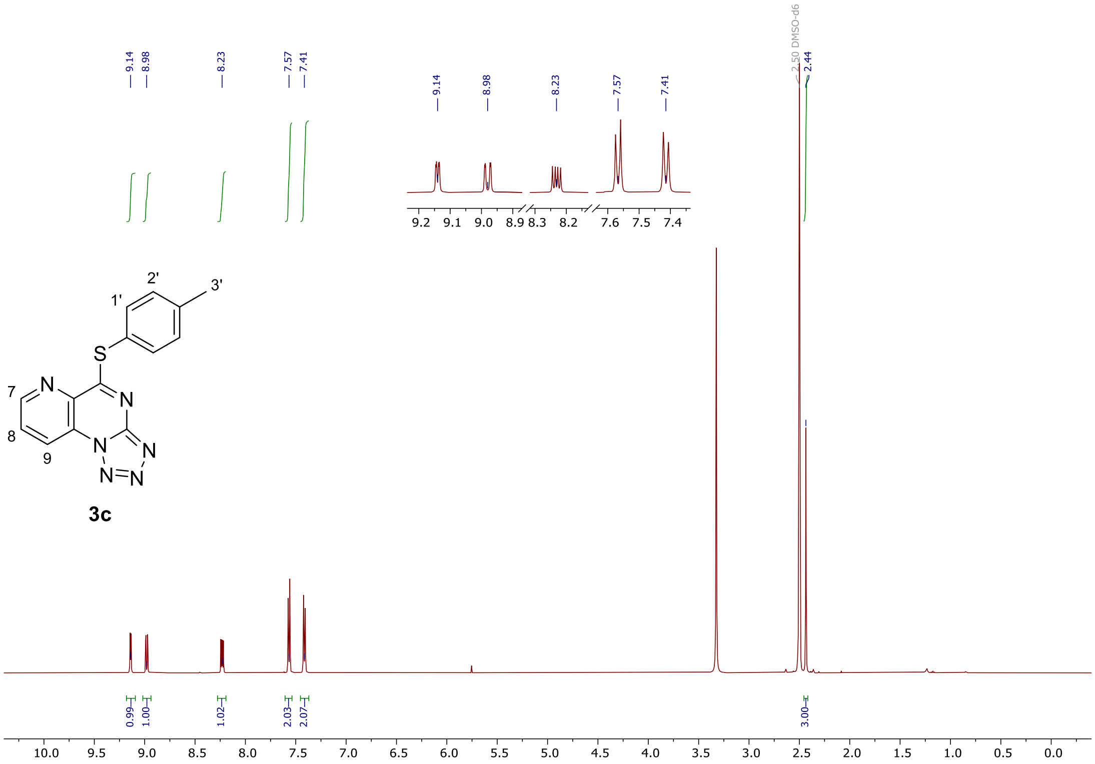

^{13}C NMR (125 MHz, DMSO-d_6)

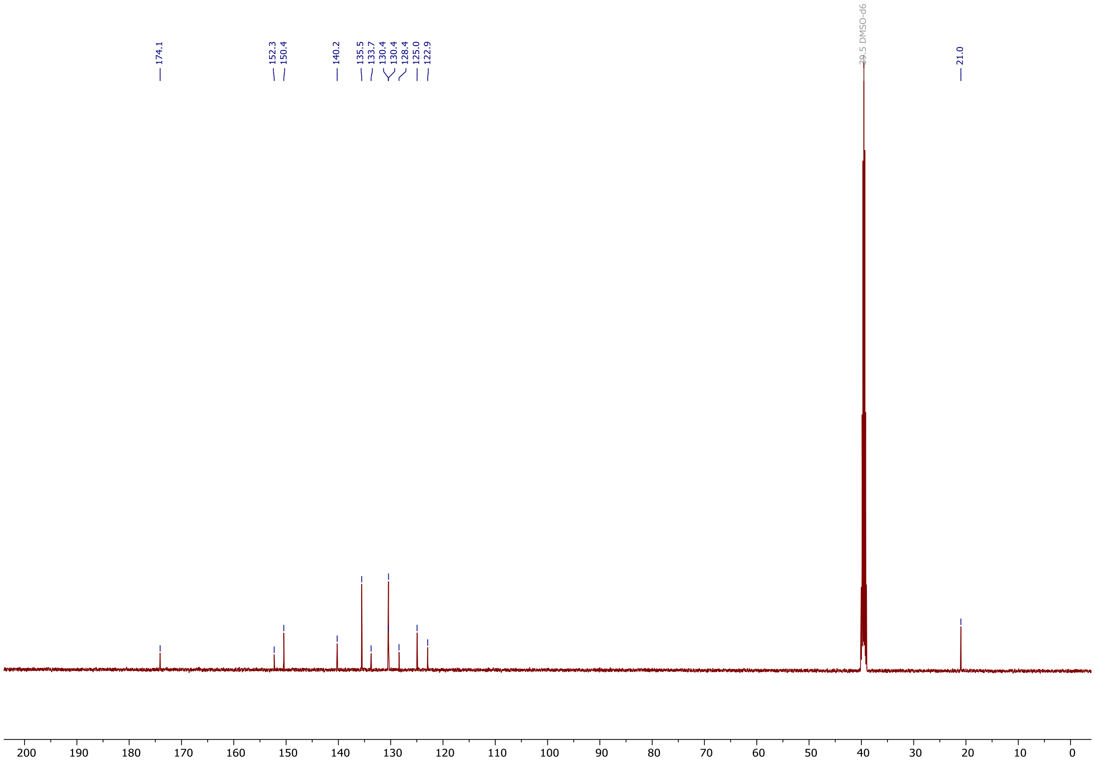

5-(*p*-Tolylthio)pyrido[2,3-*e*]tetrazolo[1,5-*a*]pyrimidine (**3c**) and 2-azido-4-(*p*-tolylthio)pyrido[3,2-*d*]pyrimidine (**3cA**):

^{1}H NMR (500 MHz, $CDCl_3$)

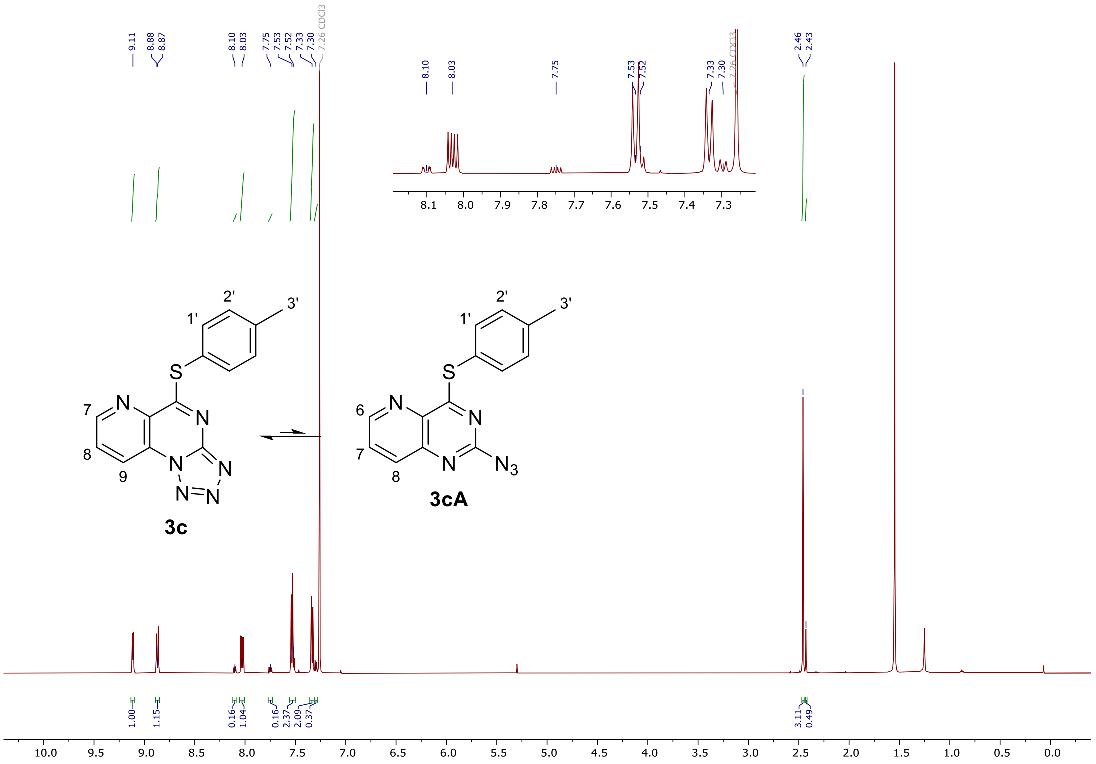

5-(Isopropylthio)pyrido[2,3-*e*]tetrazolo[1,5-*a*]pyrimidine (**3d**):

^{1}H NMR (500 MHz, DMSO-d_6)

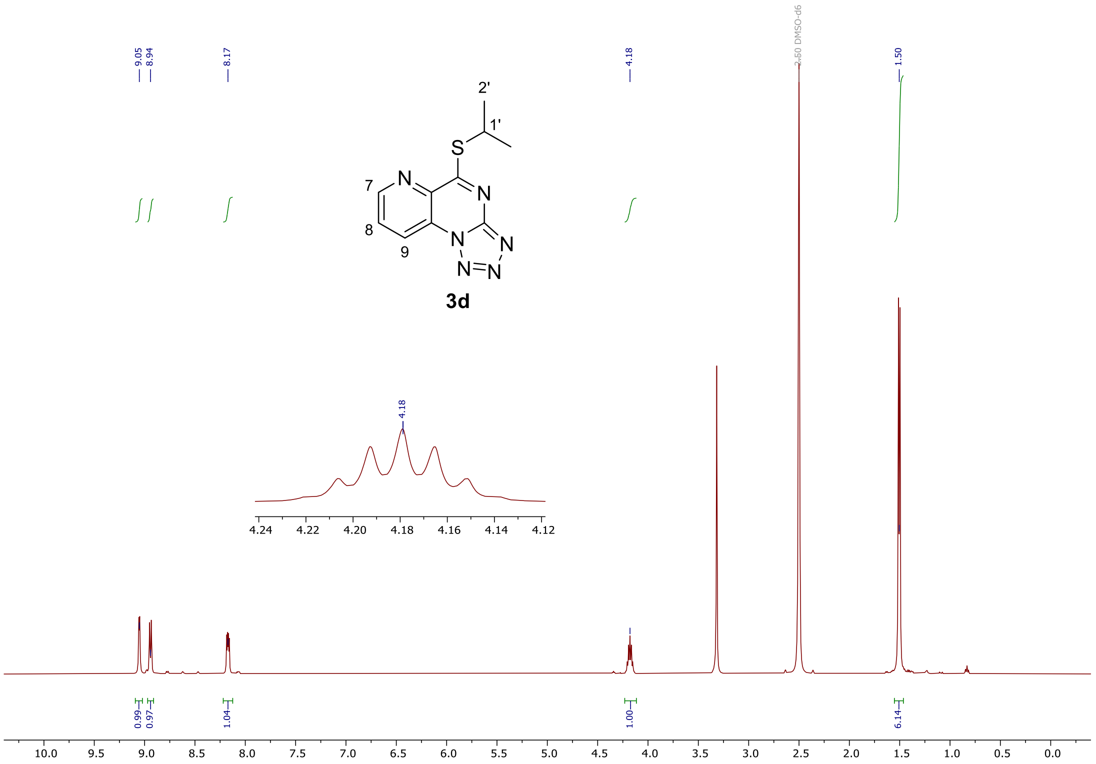

^{13}C NMR (125 MHz, DMSO-d_6)

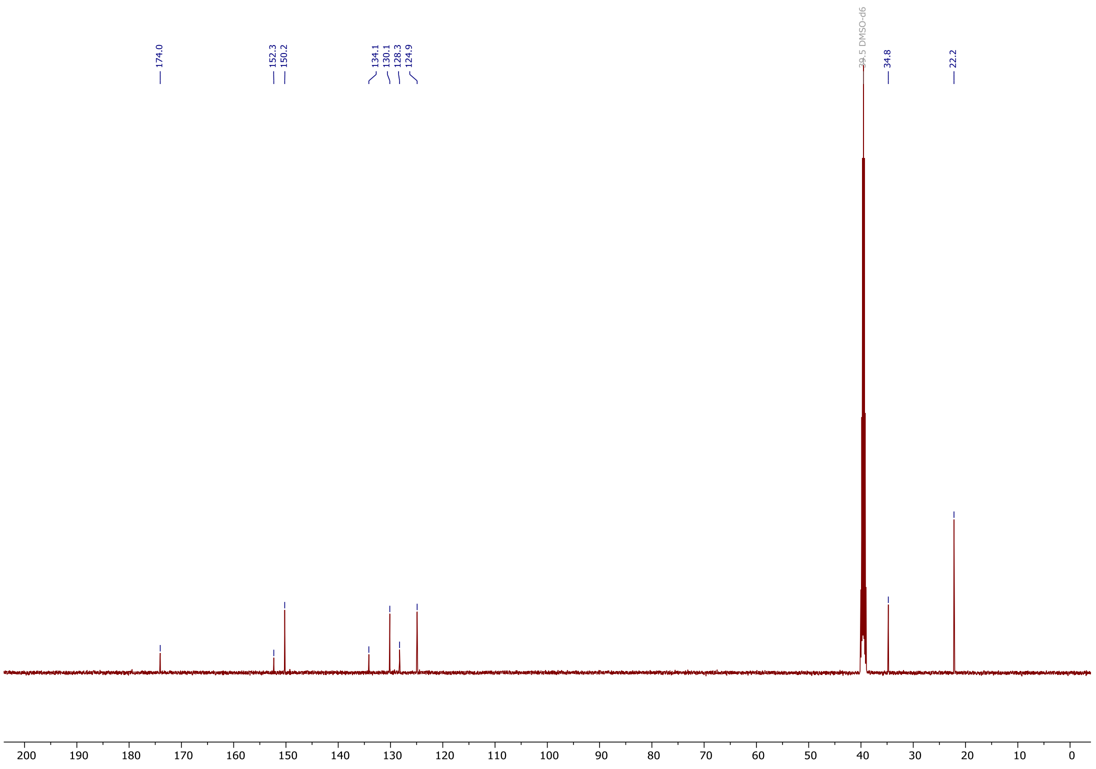

5-(Isopropylthio)pyrido[2,3-*e*]tetrazolo[1,5-*a*]pyrimidine (**3d**) and 2-azido-4-(isopropylthio)pyrido[3,2-*d*]pyrimidine (**3dA**):

^{1}H NMR (500 MHz, $CDCl_3$)

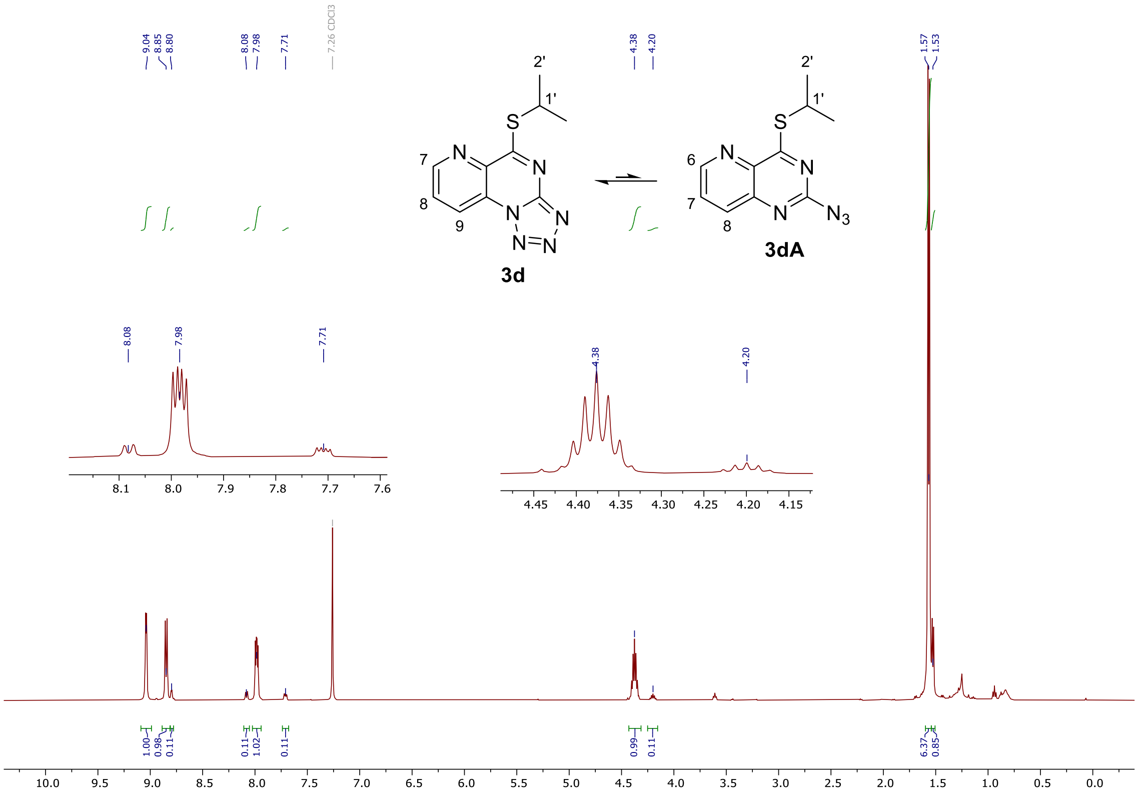

5-(Phenylthio)pyrido[2,3-*e*]tetrazolo[1,5-*a*]pyrimidine (**3e**):

^{1}H NMR (500 MHz, DMSO-d_6)

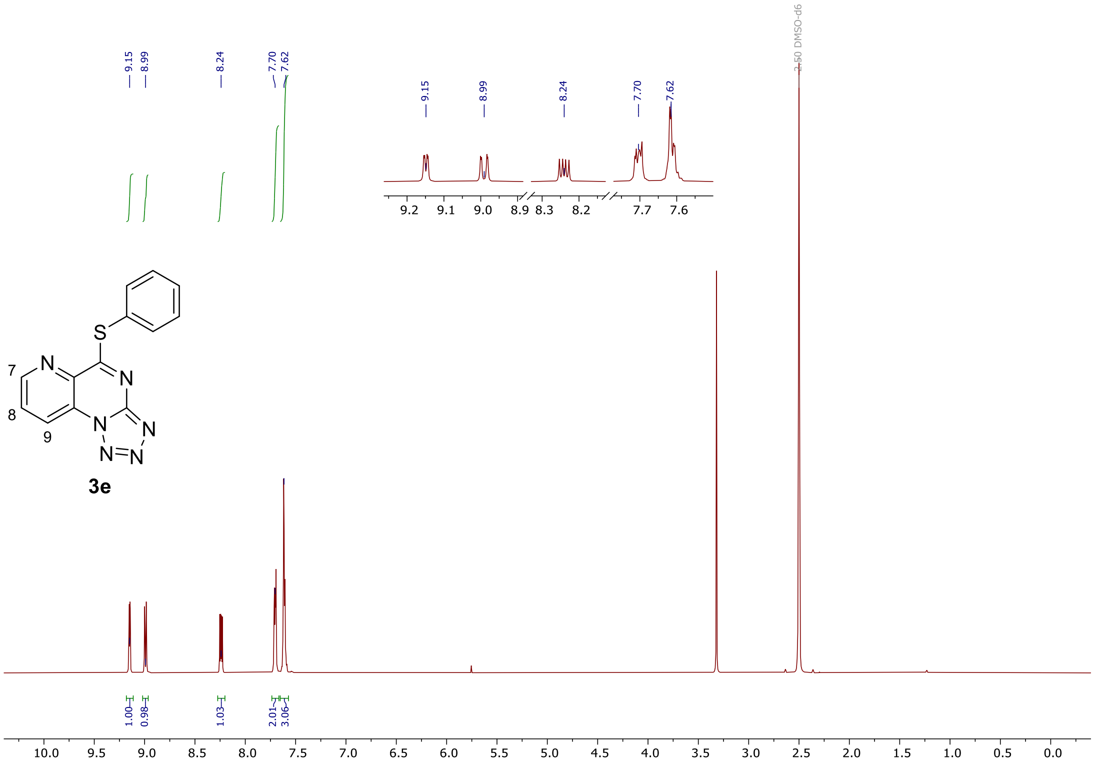

^{13}C NMR (125 MHz, DMSO-d_6)

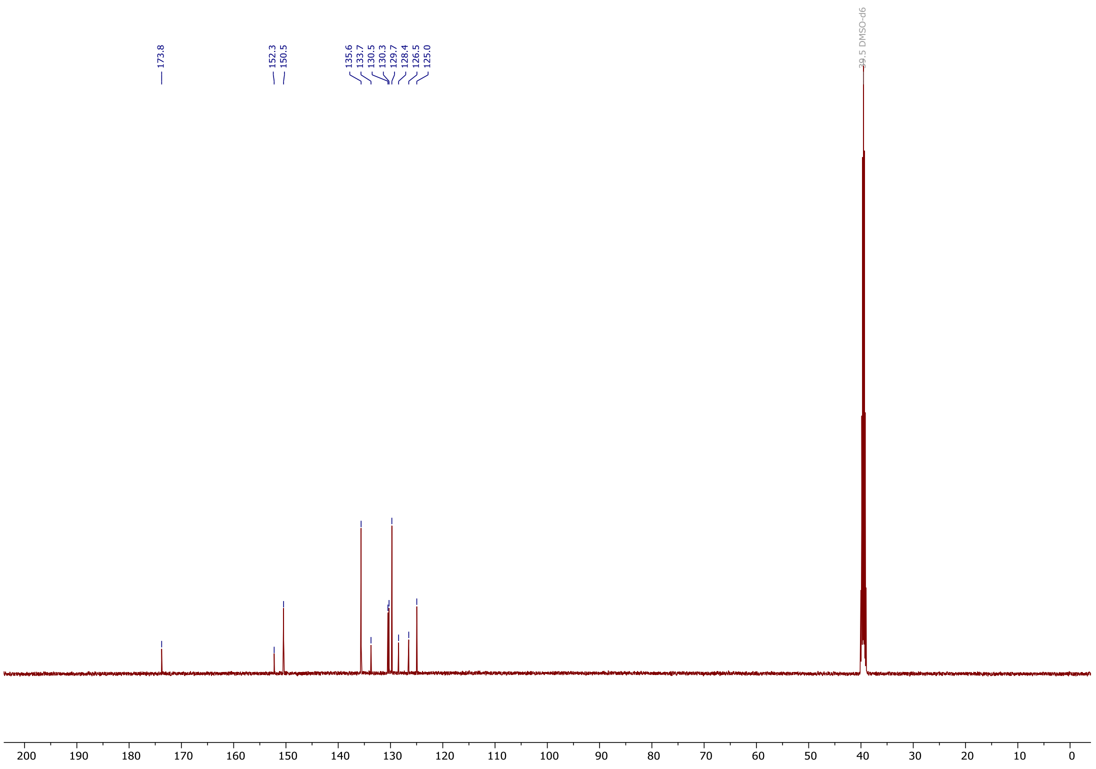

5-(Phenylthio)pyrido[2,3-*e*]tetrazolo[1,5-*a*]pyrimidine (**3e**) and 2-azido-4-(phenylthio)pyrido[3,2-*d*]pyrimidine (**3eA**):

^{1}H NMR (500 MHz, $CDCl_3$)

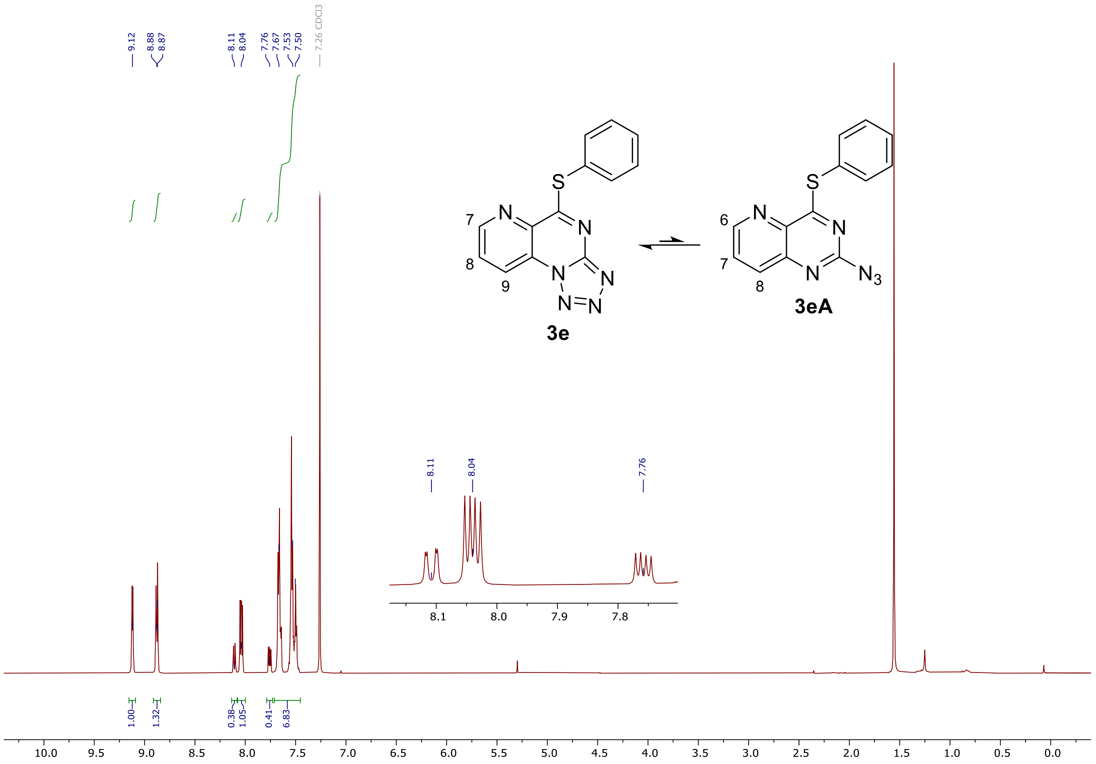

5-(Cyclohexylthio)pyrido[2,3-*e*]tetrazolo[1,5-*a*]pyrimidine (**3f**):

^{1}H NMR (500 MHz, DMSO-d_6)

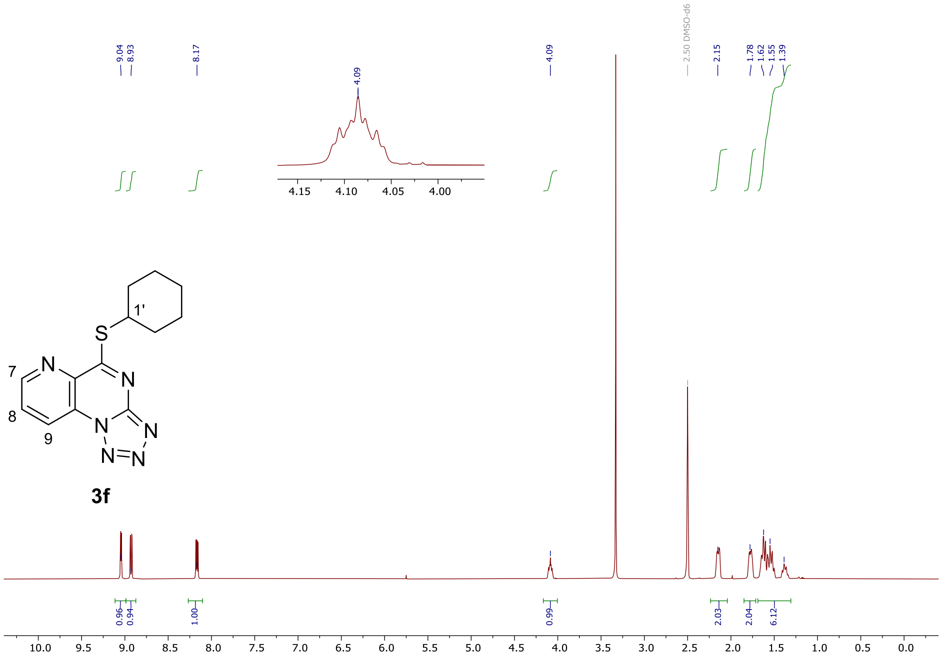

^{13}C NMR (125 MHz, DMSO-d_6)

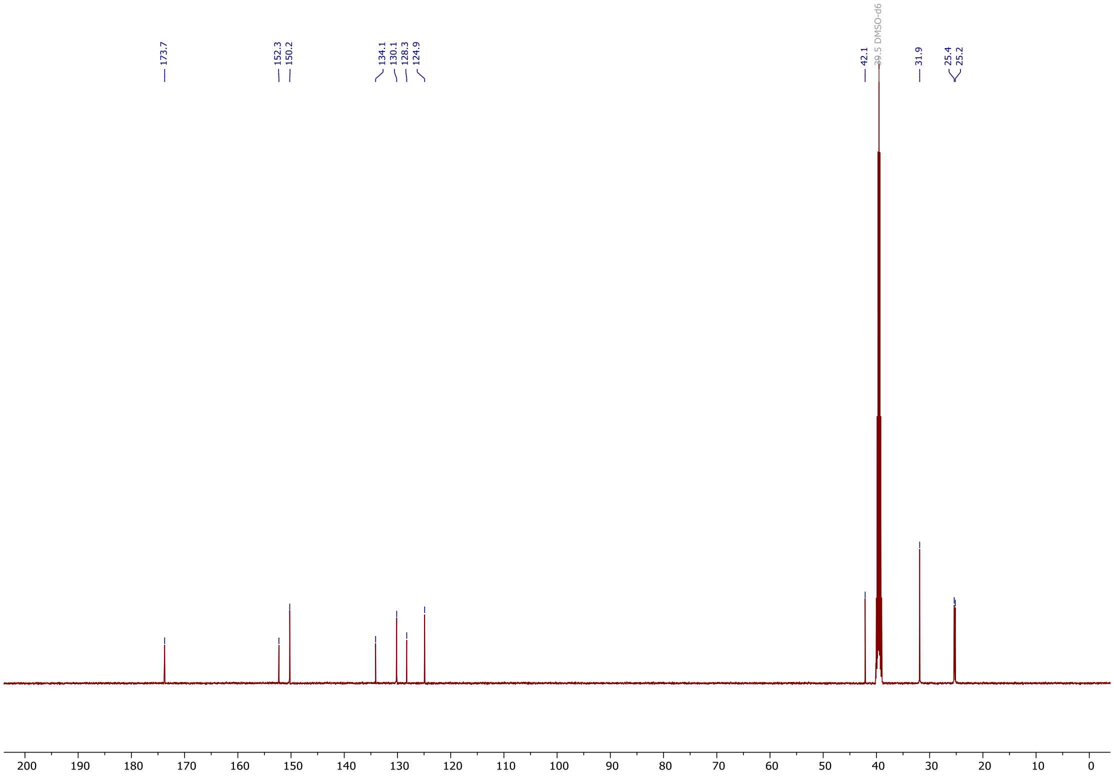

5-(Cyclohexylthio)pyrido[2,3-*e*]tetrazolo[1,5-*a*]pyrimidine (**3f**) and 2-azido-4-(cyclohexylthio)pyrido[3,2-*d*]pyrimidine (**3fA**):

^{1}H NMR (500 MHz, $CDCl_3$)

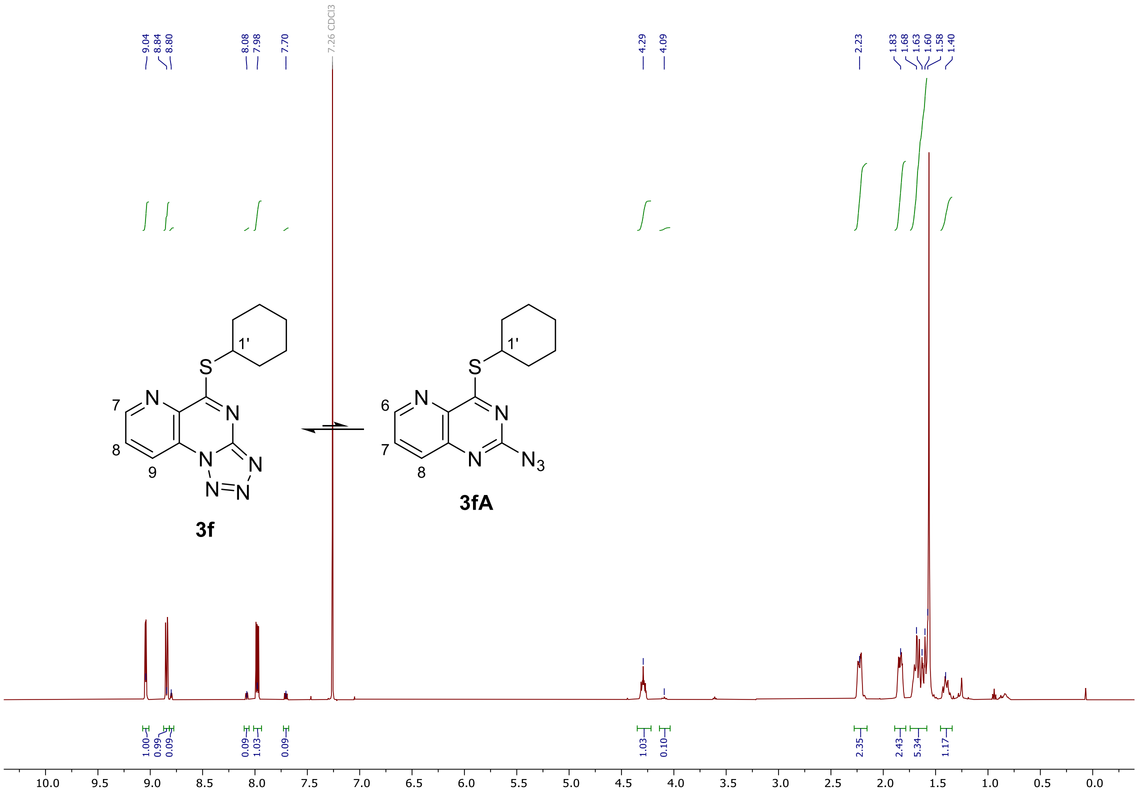

N-(4-Methoxybenzyl)pyrido[2,3-*e*]tetrazolo[1,5-*a*]pyrimidin-5-amine (**4a**):

^{1}H NMR (500 MHz, DMSO-d_6)

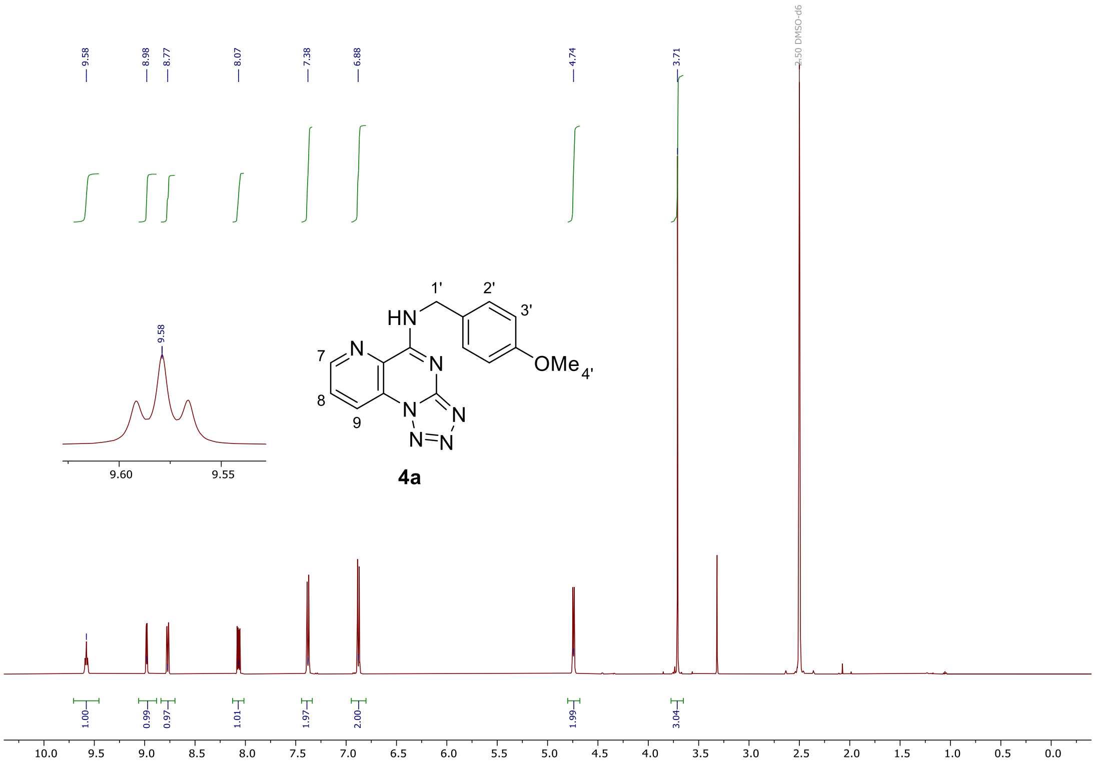

^{13}C NMR (125 MHz, DMSO-d_6)

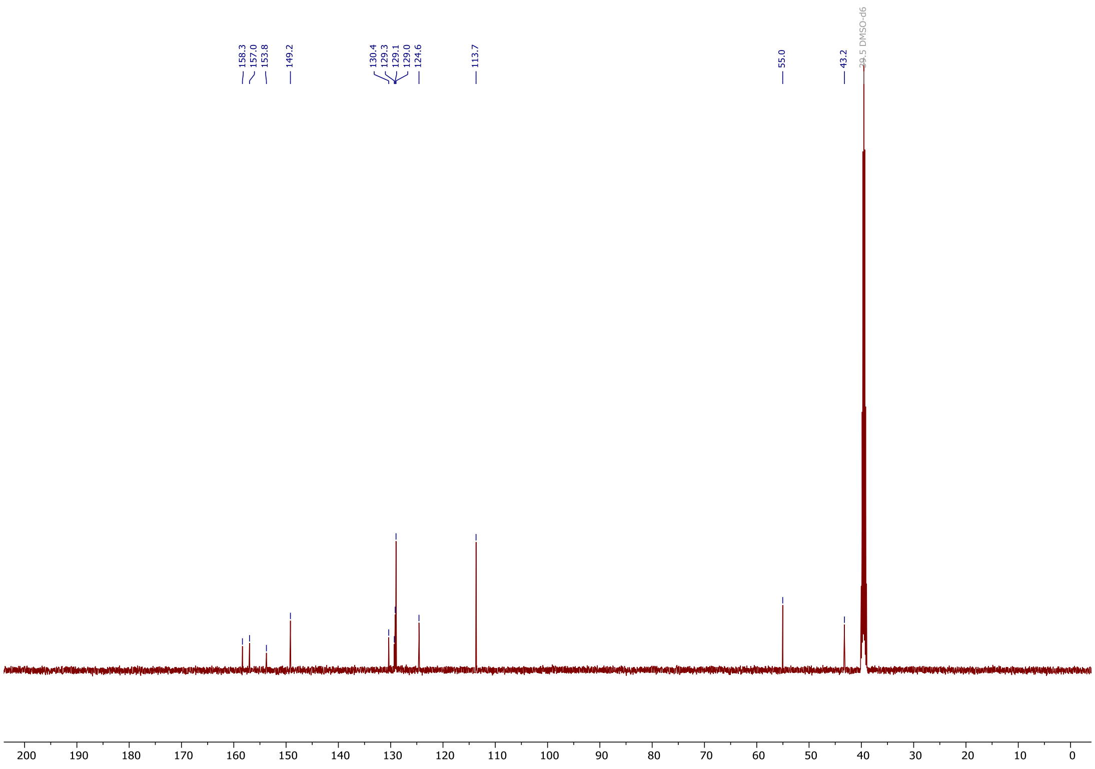

N-(4-Methoxybenzyl)pyrido[2,3-*e*]tetrazolo[1,5-*a*]pyrimidin-5-amine (**4a**) and 2-azido-*N*-(4-methoxybenzyl)pyrido[3,2-*d*]pyrimidin-4-amine (**4aA**):

^{1}H NMR (500 MHz, $CDCl_3$)

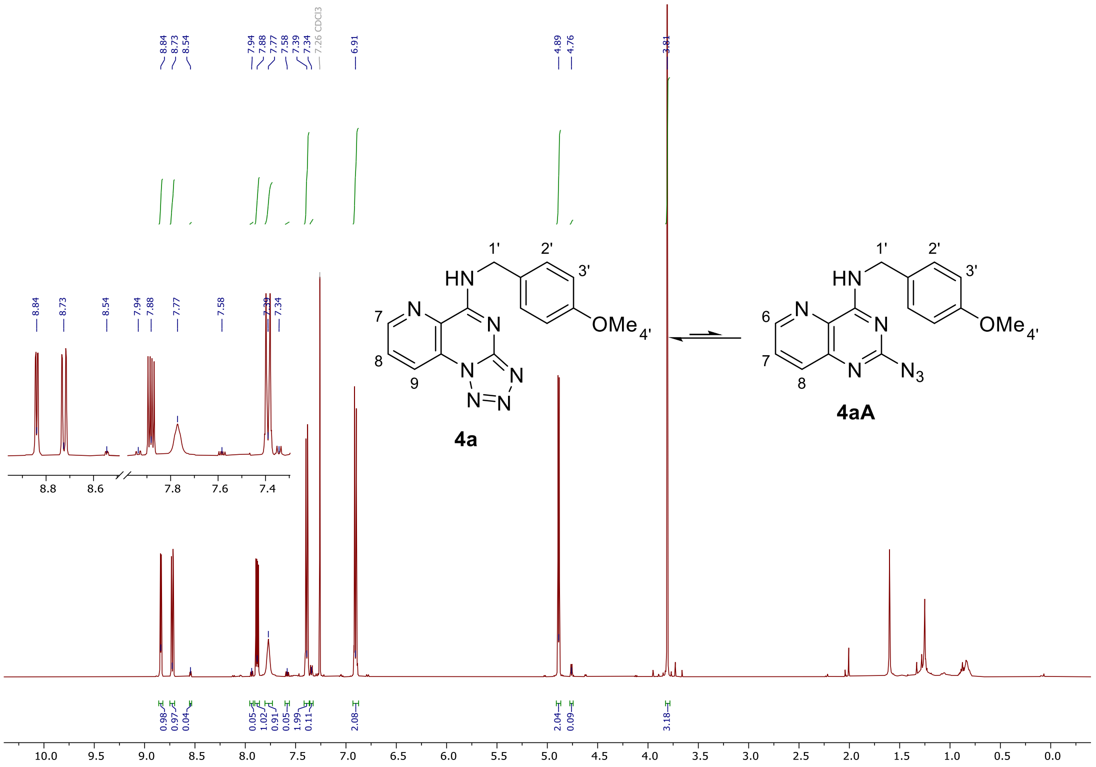

N-Hexylpyrido[2,3-*e*]tetrazolo[1,5-*a*]pyrimidin-5-amine (**4b**):

^{1}H NMR (500 MHz, DMSO-d_6)

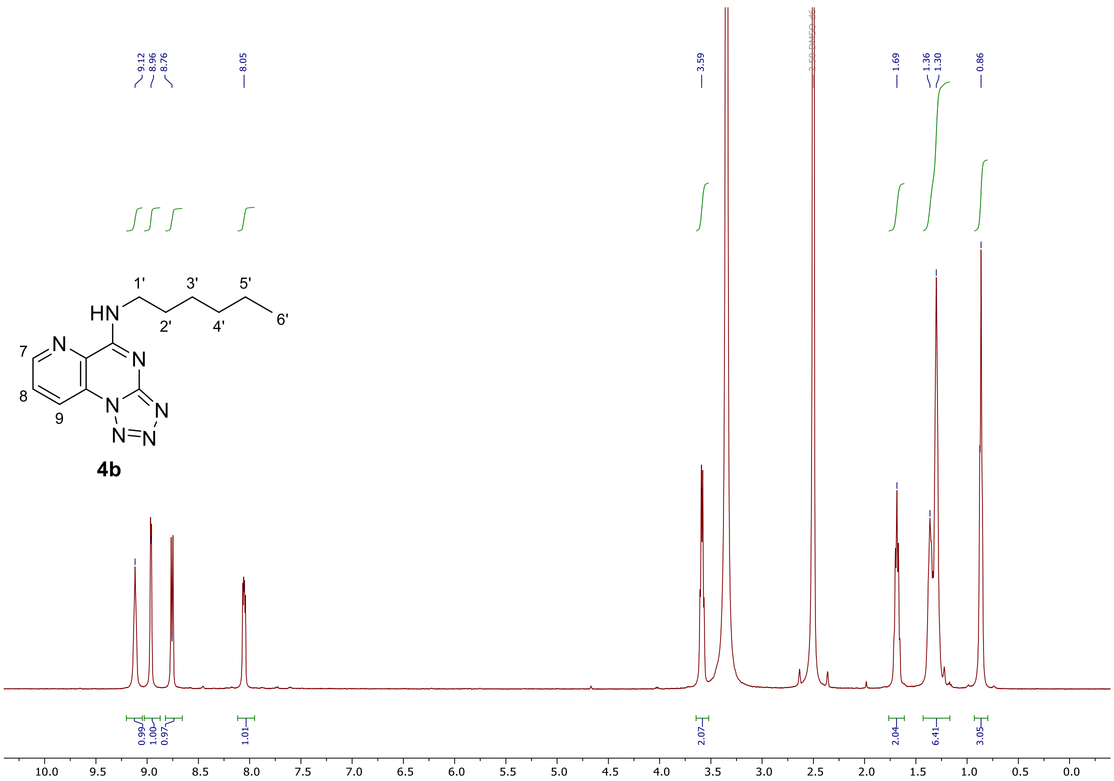

^{13}C NMR (125 MHz, DMSO-d_6)

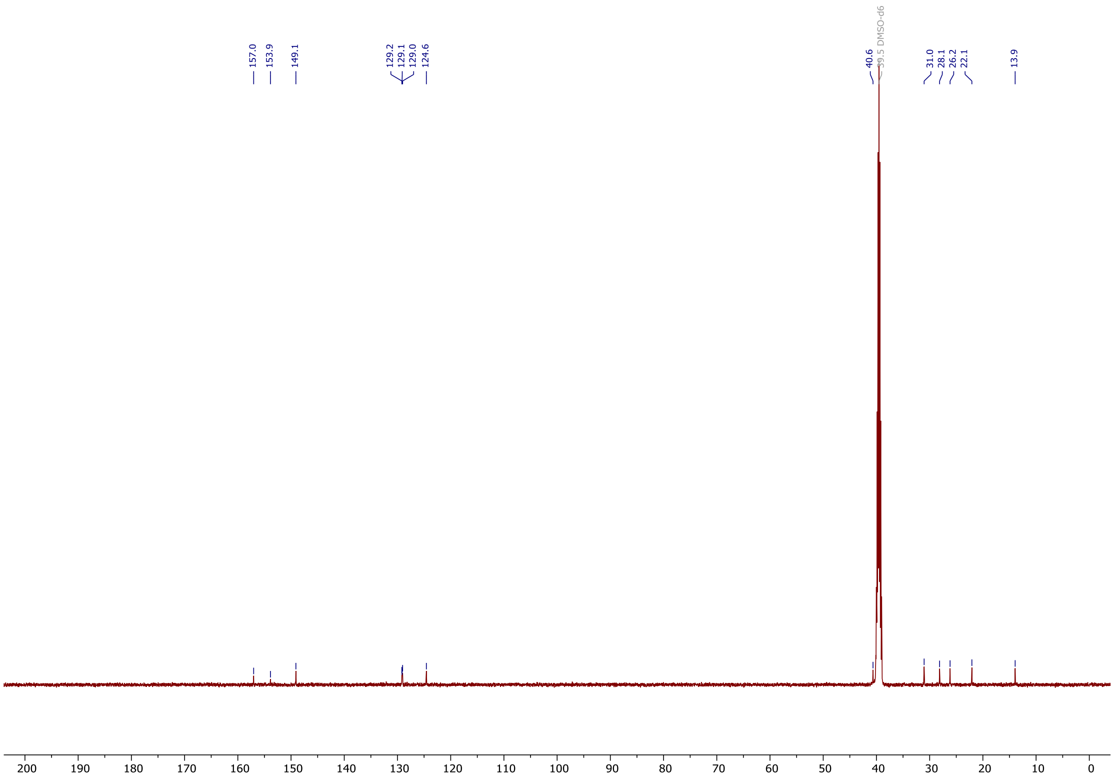

2-Azido-*N*-hexylpyrido[3,2-*d*]pyrimidin-4-amine (**4bA**) and *N*-hexylpyrido[2,3-*e*]tetrazolo[1,5-*a*]pyrimidin-5-amine (**4b**):

^{1}H NMR (500 MHz, $CDCl_3$)

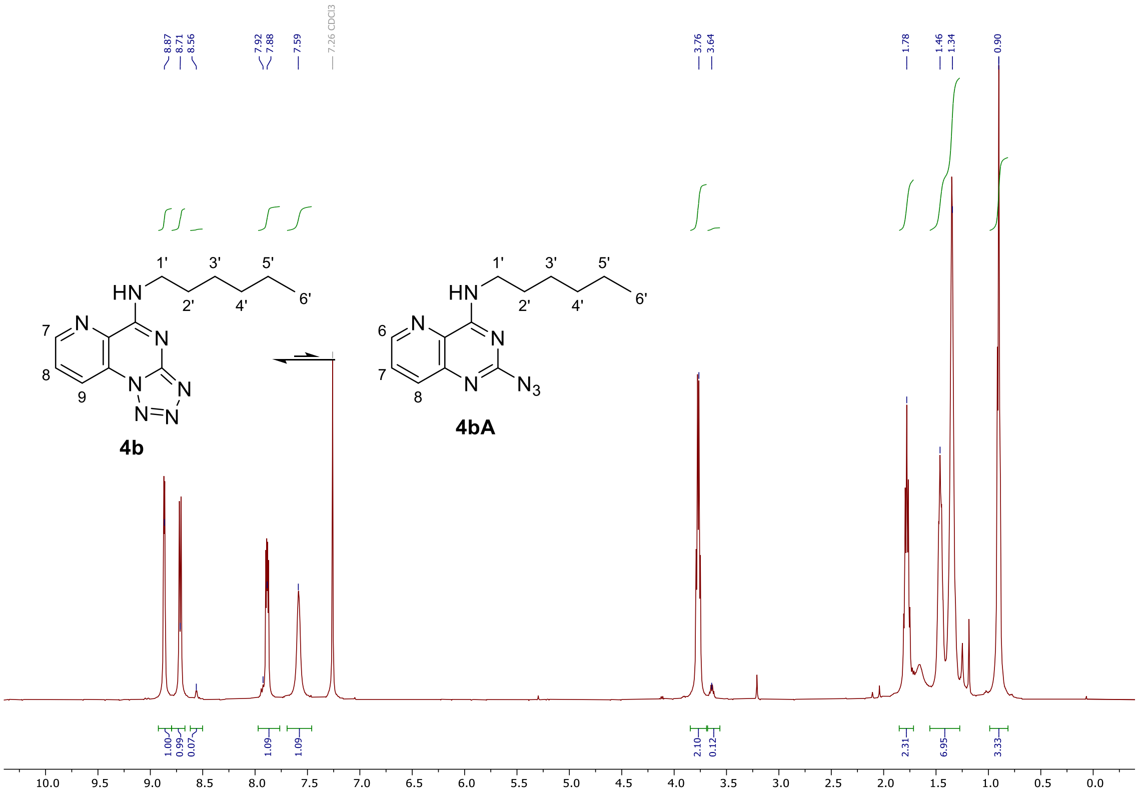

5-(Piperidin-1-yl)pyrido[2,3-*e*]tetrazolo[1,5-*a*]pyrimidine (**4c**):

^{1}H NMR (500 MHz, DMSO-d_6)

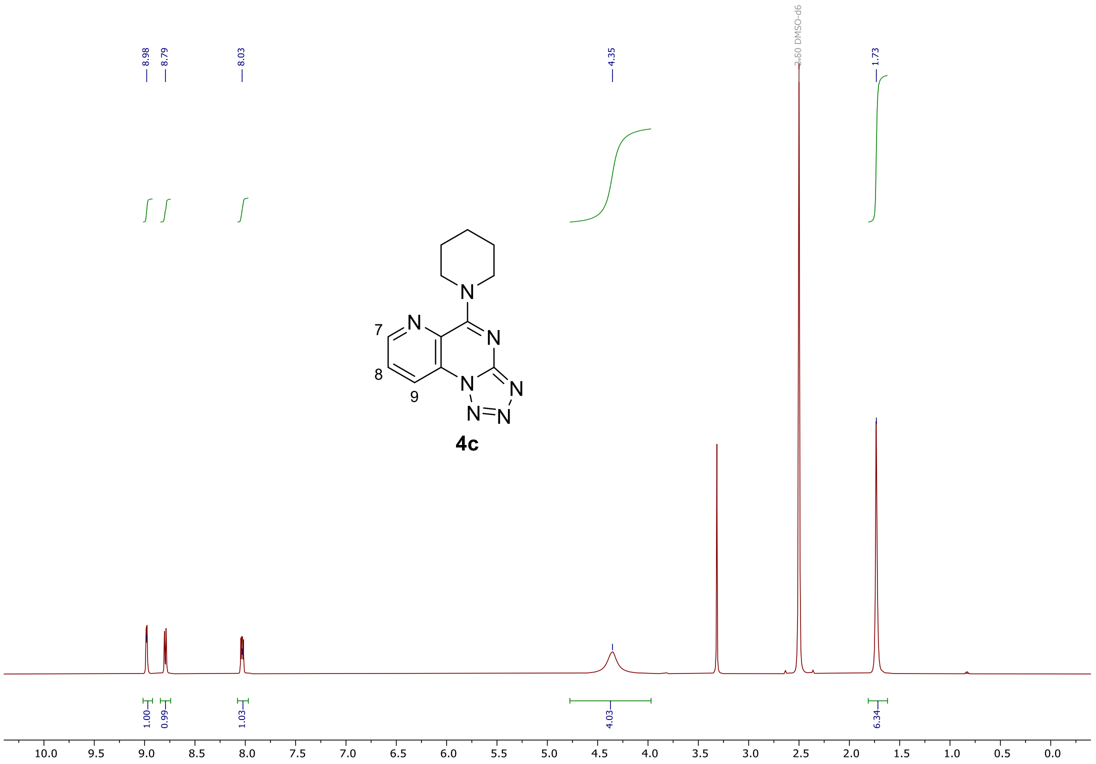

^{13}C NMR (125 MHz, DMSO-d_6)

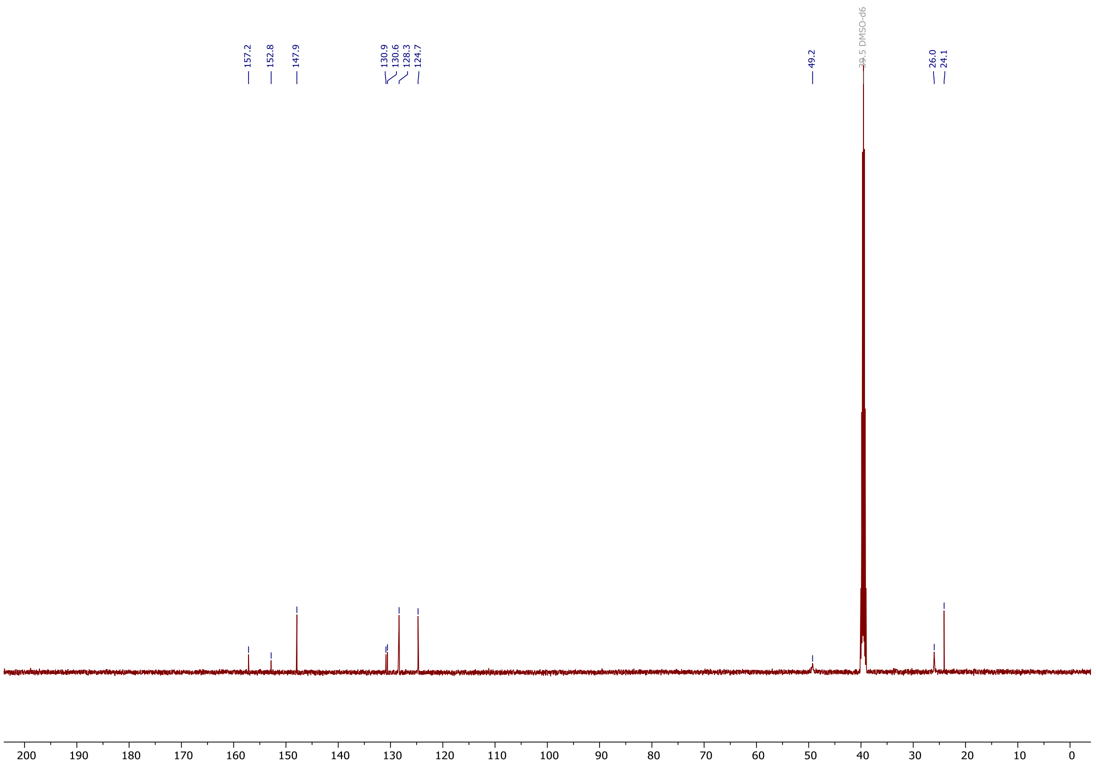

4-(Pyrido[2,3-*e*]tetrazolo[1,5-*a*]pyrimidin-5-yl)morpholine (**4c**) and 2-azido-4-(piperidin-1-yl)pyrido[3,2-*d*]pyrimidine (**4cA**):

^{1}H NMR (500 MHz, $CDCl_3$)

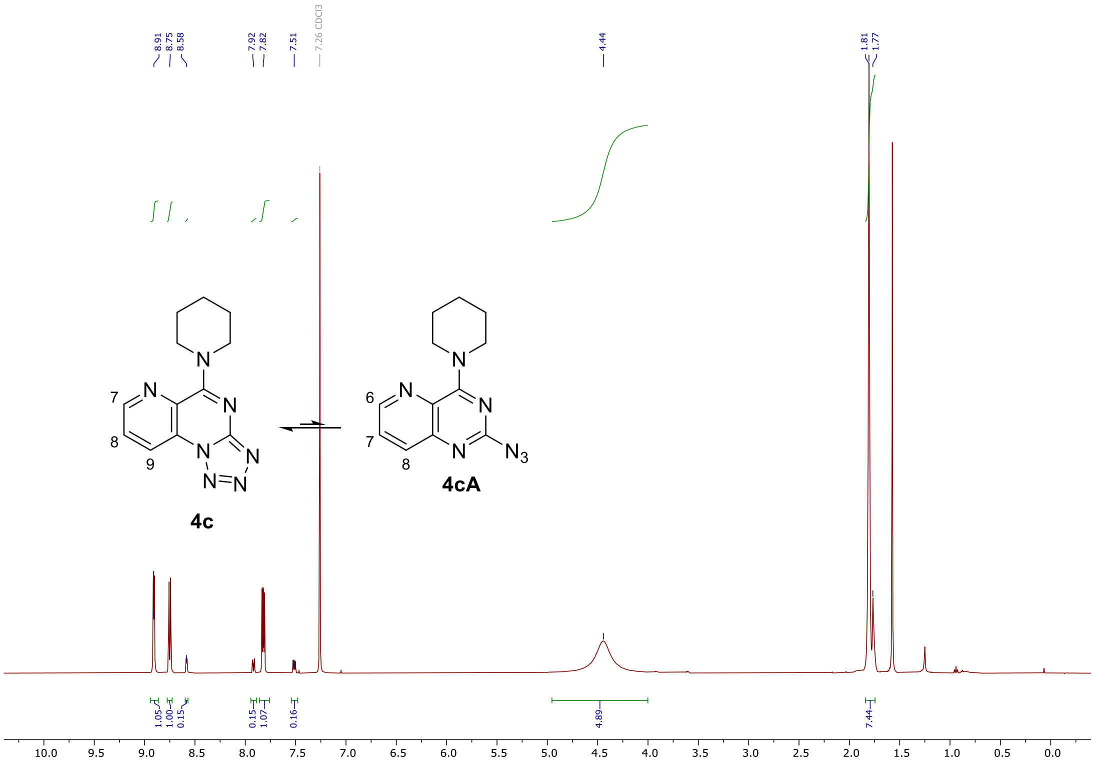

4-(Pyrido[2,3-*e*]tetrazolo[1,5-*a*]pyrimidin-5-yl)morpholine (**4d**):

^{1}H NMR (500 MHz, DMSO-d_6)

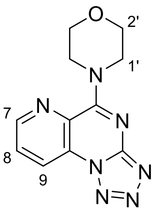

4d

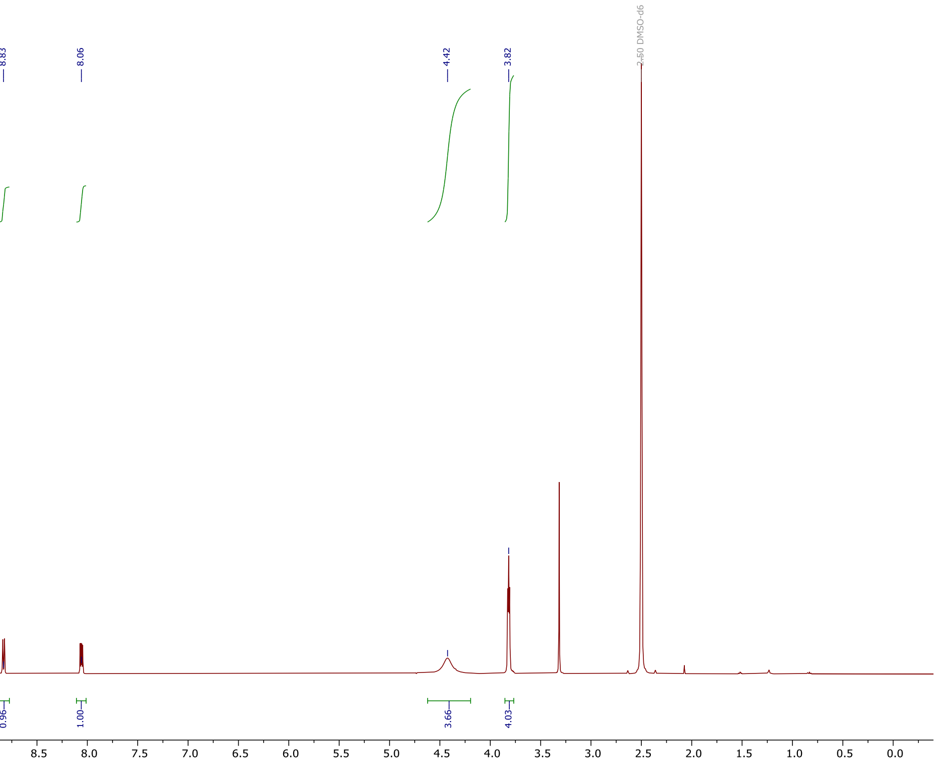

^{13}C NMR (125 MHz, DMSO-d_6)

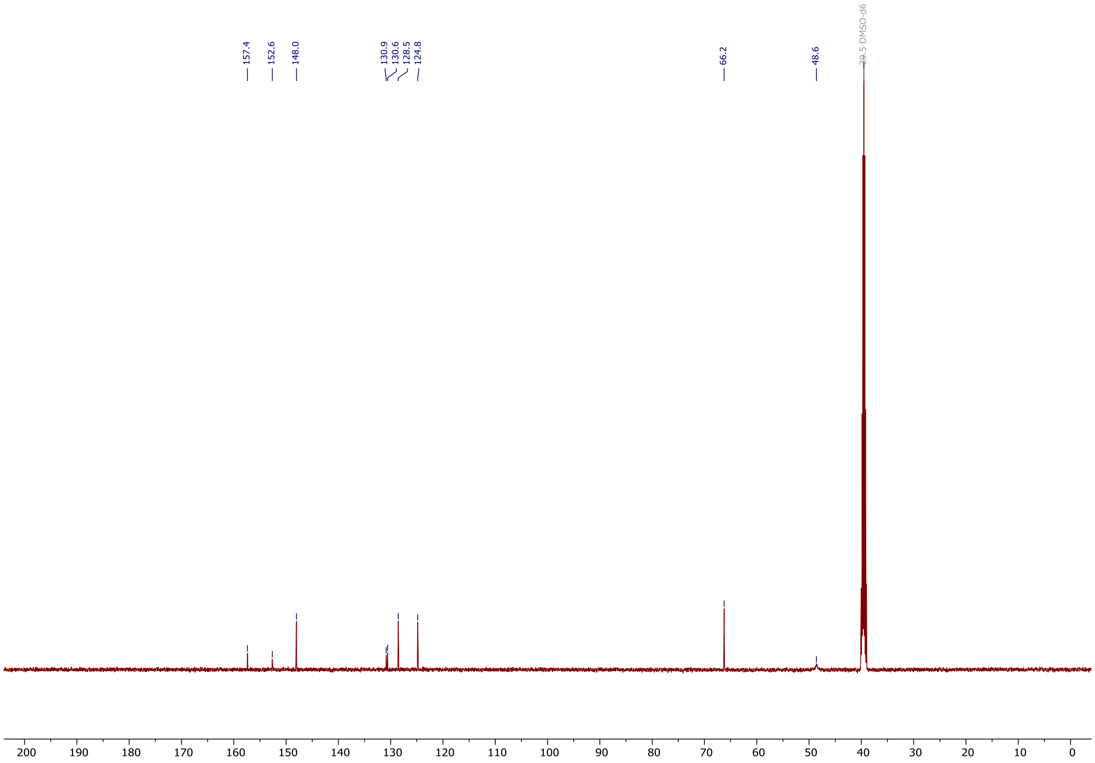

4-(Pyrido[2,3-*e*]tetrazolo[1,5-*a*]pyrimidin-5-yl)morpholine (**4d**) and 4-(2-azidopyrido[3,2-*d*]pyrimidin-4-yl)morpholine (**4dA**):

^{1}H NMR (500 MHz, $CDCl_3$)

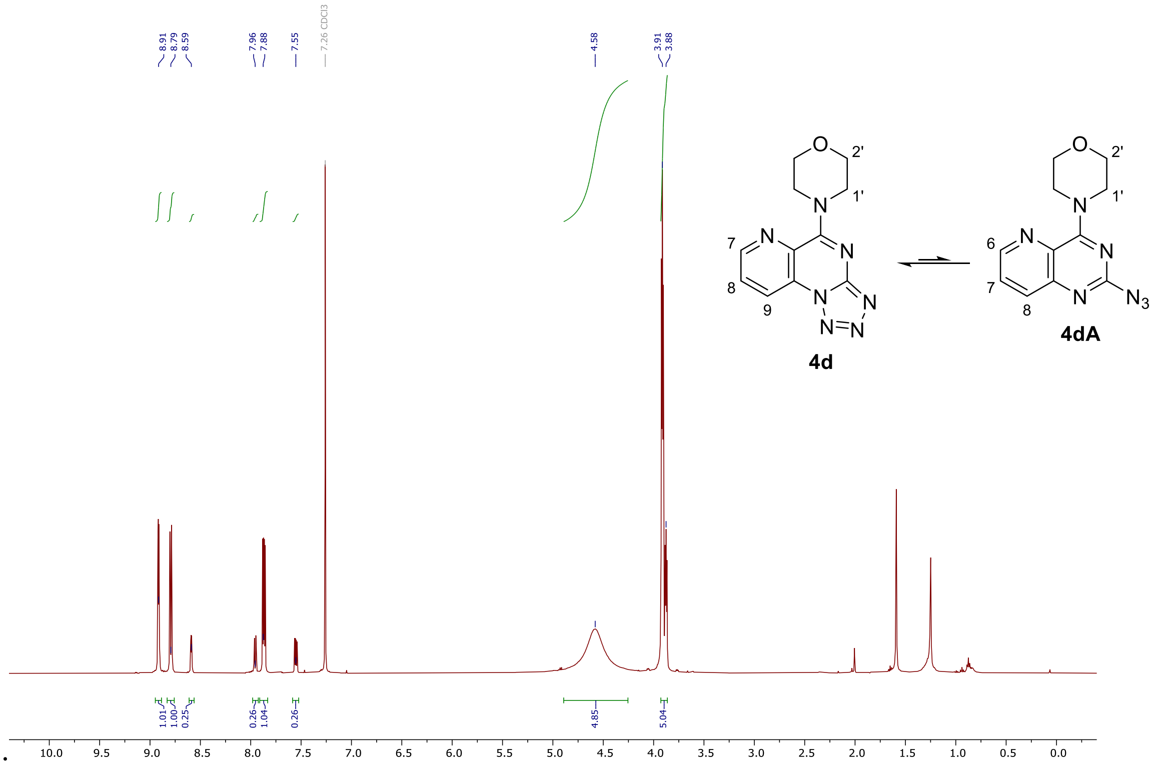

Pyrido[2,3-*e*]tetrazolo[1,5-*a*]pyrimidin-5-amine (**4e**):

^{1}H NMR (500 MHz, DMSO-d_6)

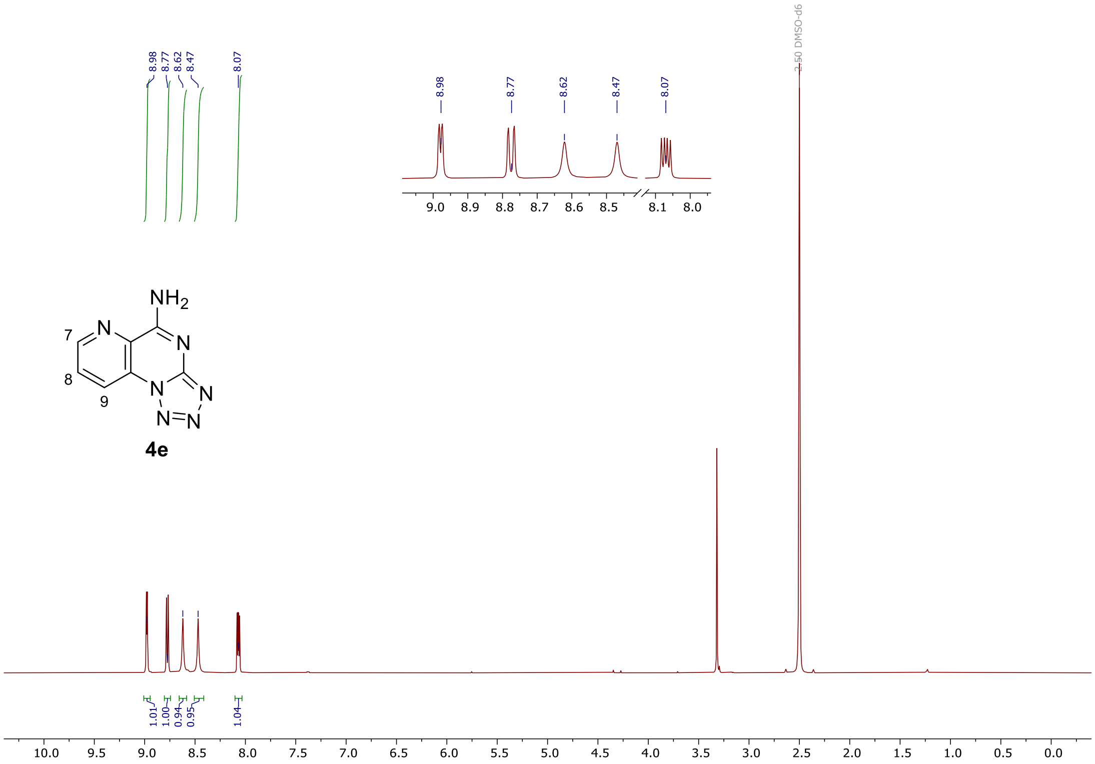

^{13}C NMR (125 MHz, DMSO-d_6)

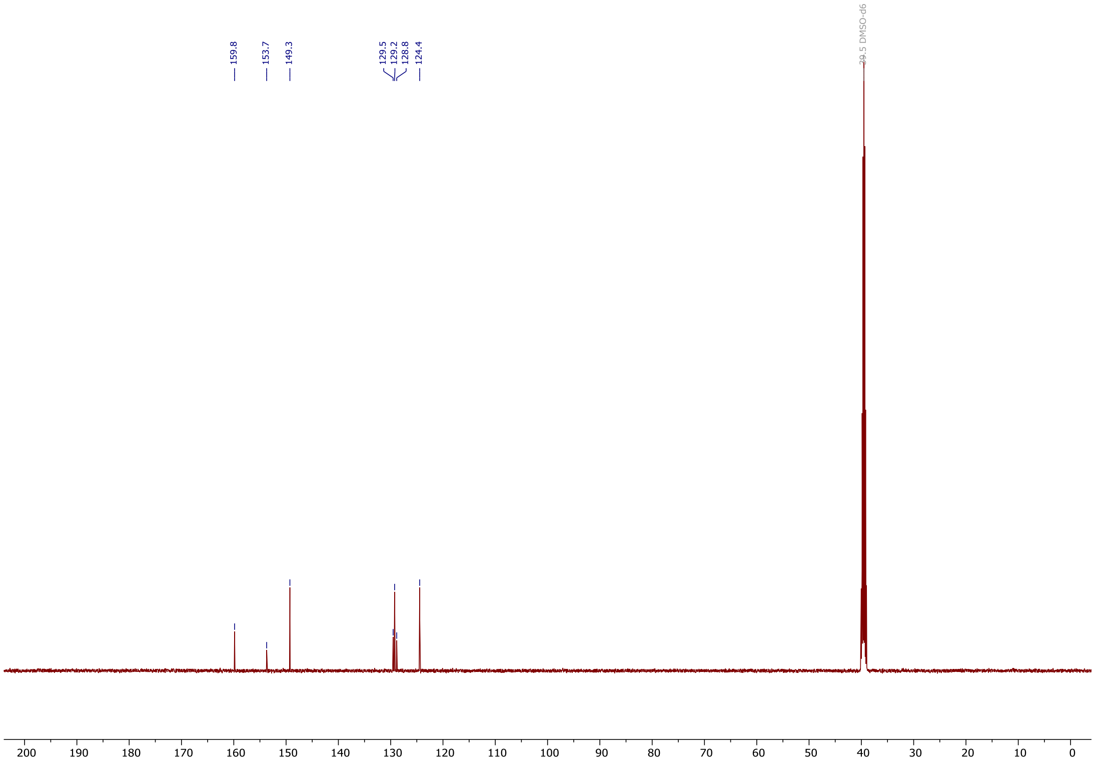

5-Hydrazinylpyrido[2,3-*e*]tetrazolo[1,5-*a*]pyrimidine (**4f**):

^{1}H NMR (500 MHz, DMSO-d_6)

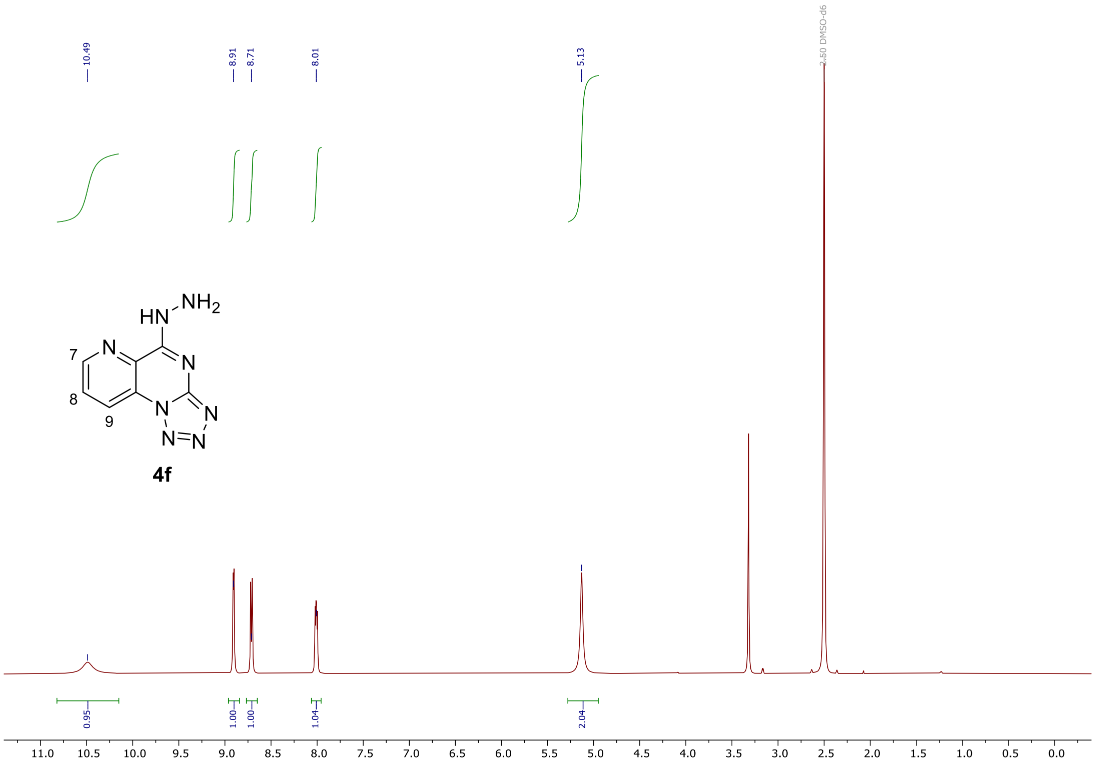

^{13}C NMR (125 MHz, DMSO-d_6)

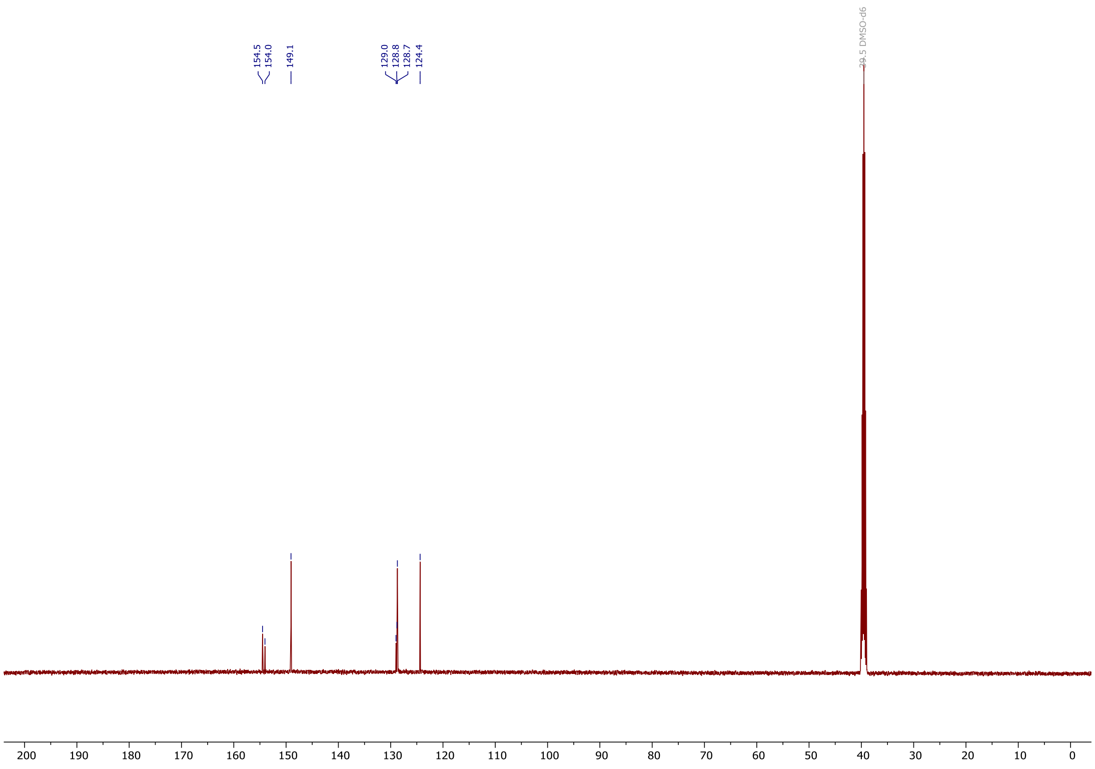

5-(4-Methylpiperazin-1-yl)pyrido[2,3-*e*]tetrazolo[1,5-*a*]pyrimidine (**4g**):

^{1}H NMR (500 MHz, DMSO-d_6)

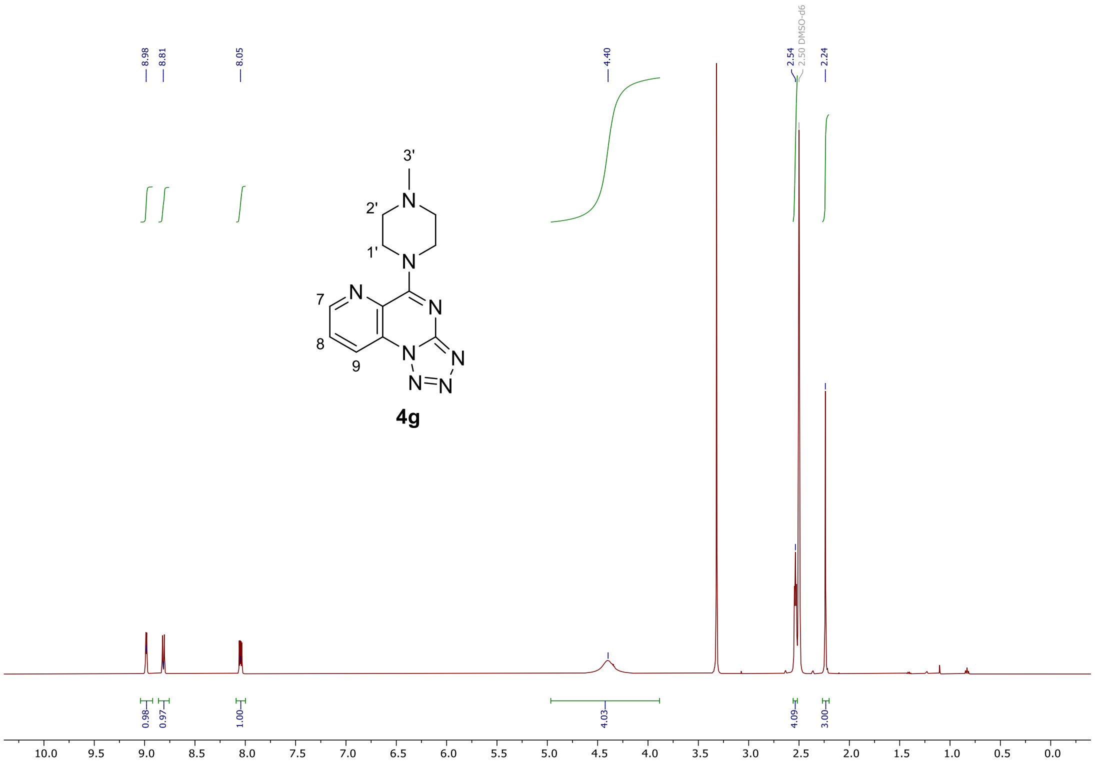

^{13}C NMR (125 MHz, DMSO-d_6)

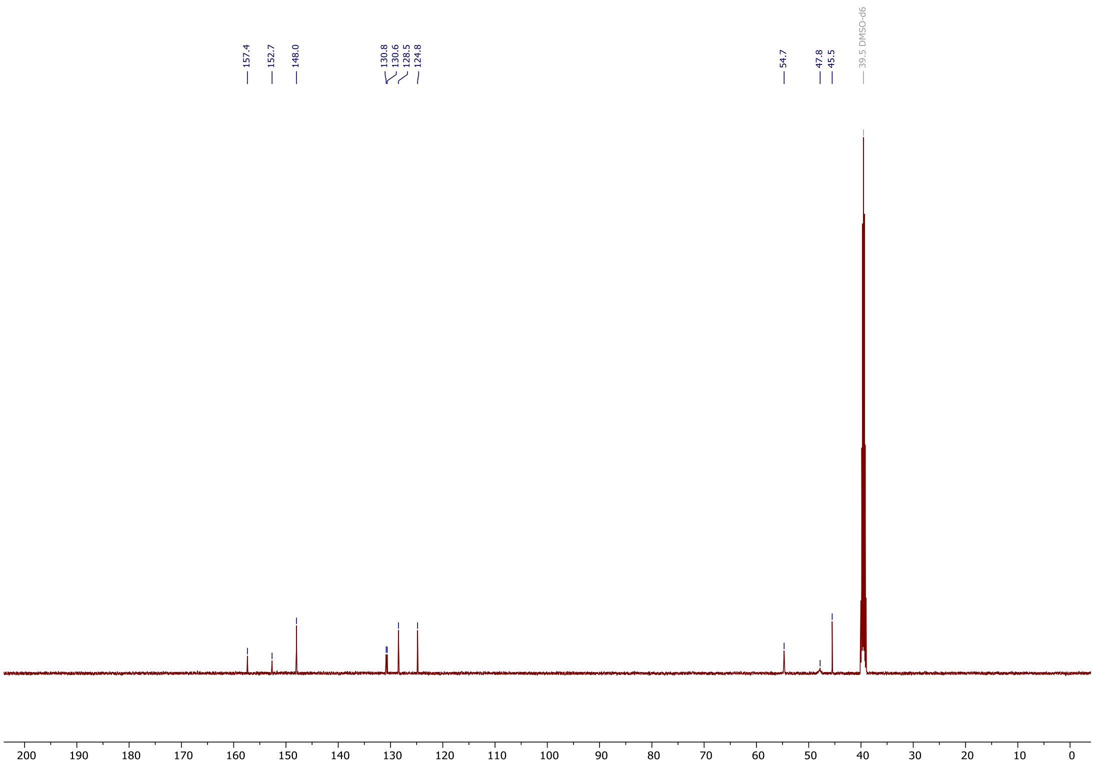

5-(4-Methylpiperazin-1-yl)pyrido[2,3-*e*]tetrazolo[1,5-*a*]pyrimidine (**4g**) and 2-azido-4-(4-methylpiperazin-1-yl)pyrido[3,2-*d*]pyrimidine (**4gA**):

^{1}H NMR (500 MHz, $CDCl_3$)

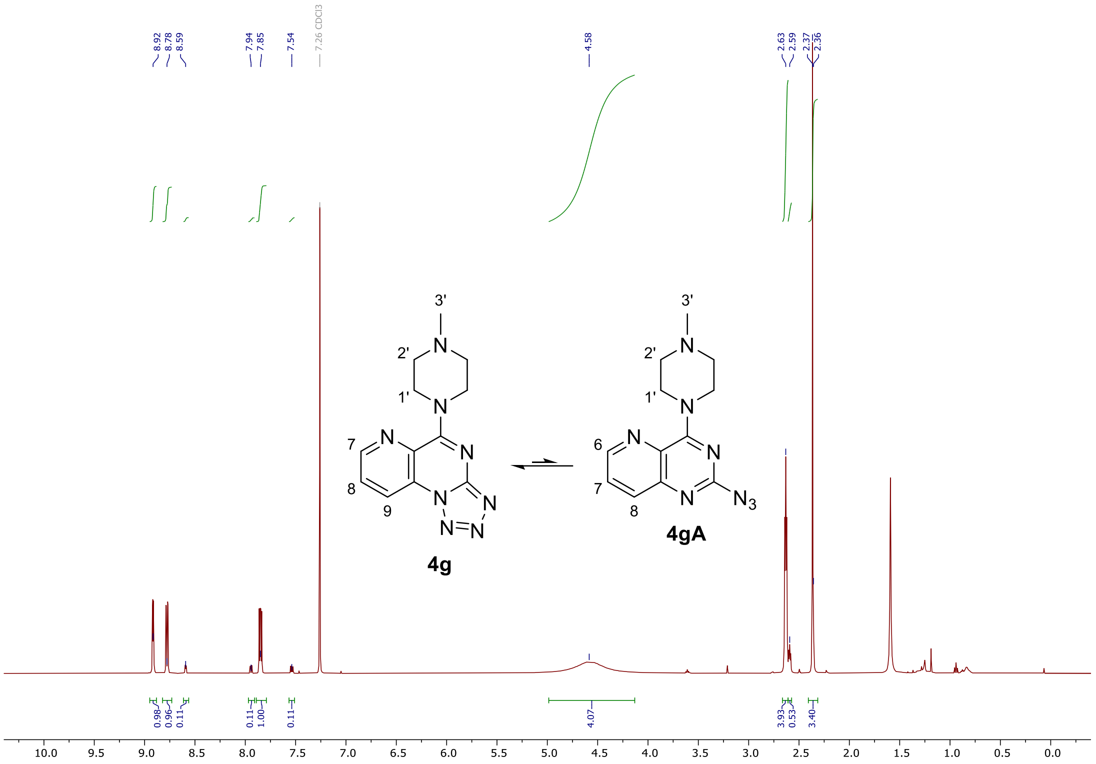

5-(Cyclopentyloxy)pyrido[2,3-*e*]tetrazolo[1,5-*a*]pyrimidine (**5a**):

^{1}H NMR (500 MHz, DMSO-d_6)

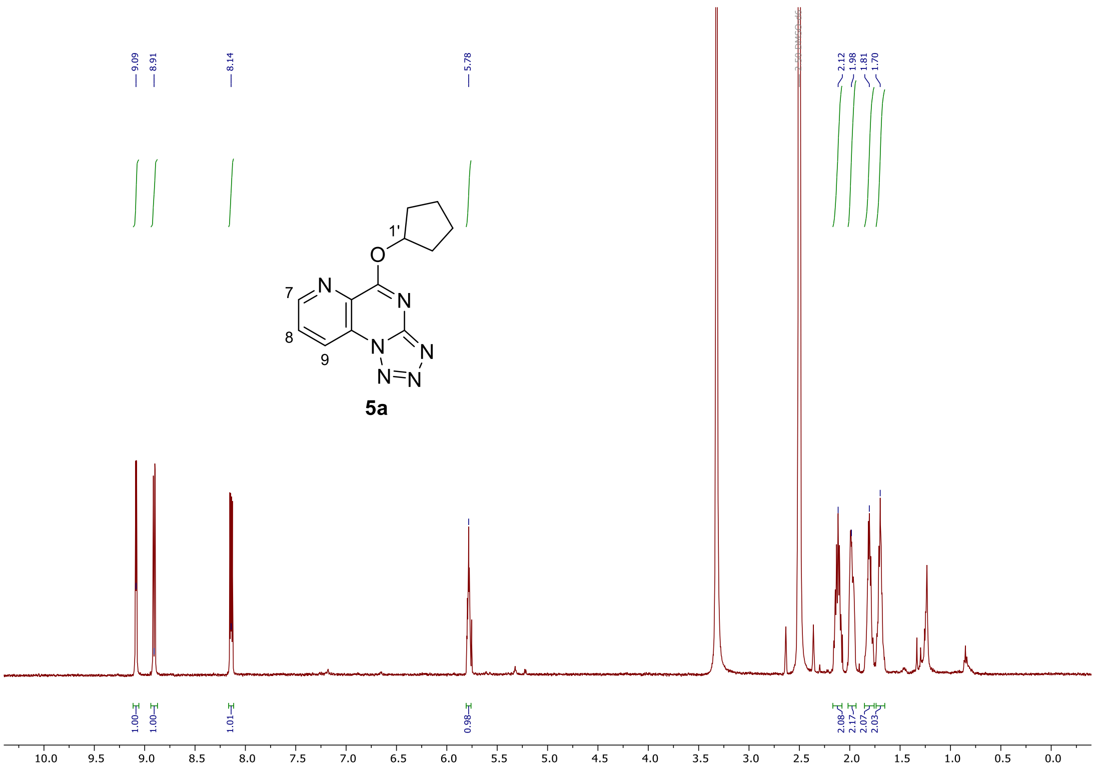

^{13}C NMR (125 MHz, DMSO-d_6)

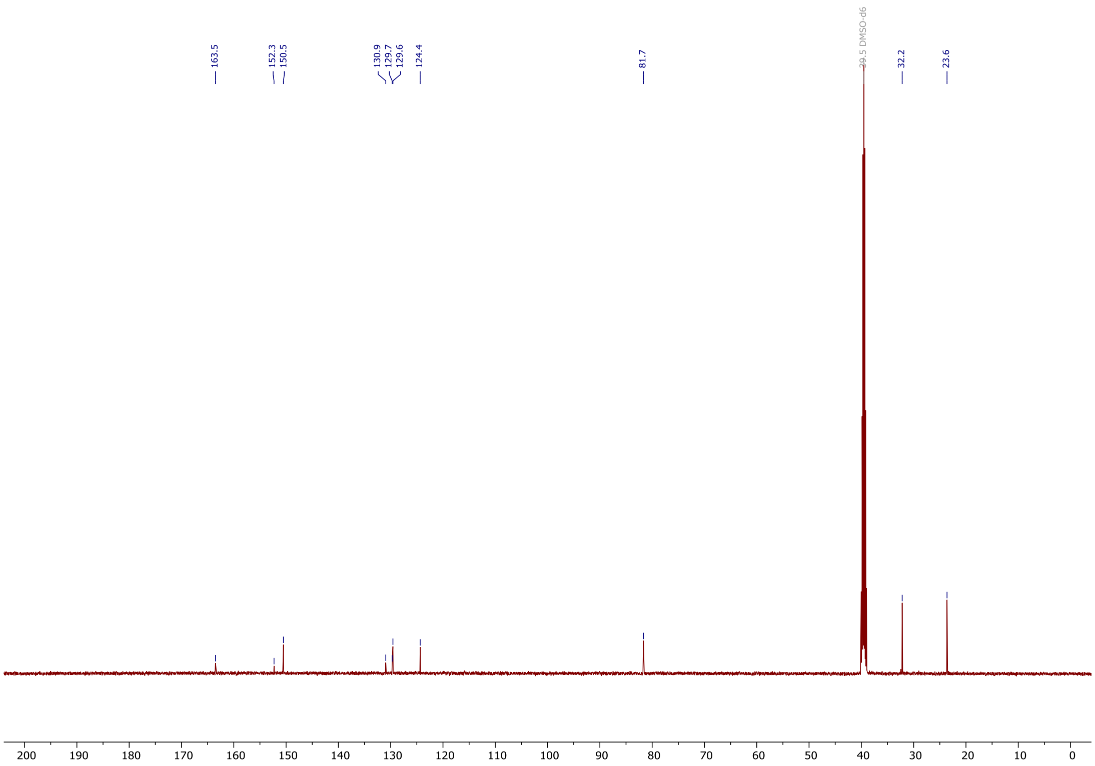

5-(Cyclopentyloxy)pyrido[2,3-*e*]tetrazolo[1,5-*a*]pyrimidine (**5a**) and 2-azido-4-(cyclopentyloxy)pyrido[3,2-*d*]pyrimidine (**5aA**):

^{1}H NMR (500 MHz, $CDCl_3$)

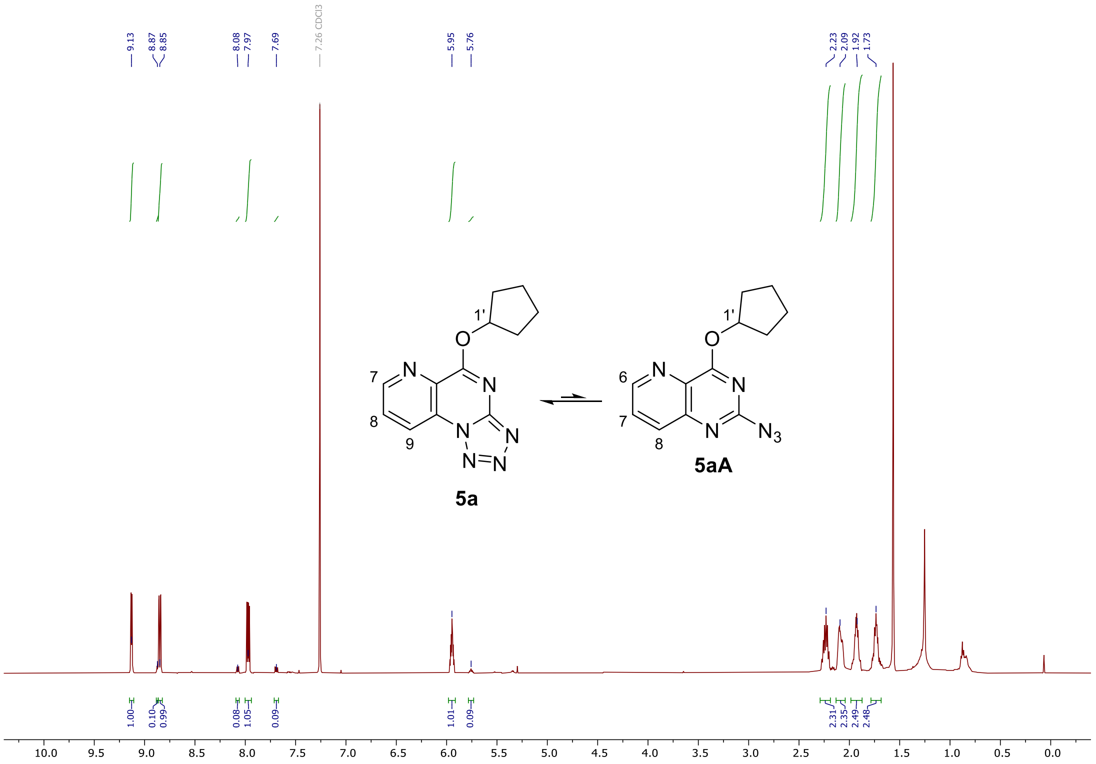

5-Propoxypyrido[2,3-*e*]tetrazolo[1,5-*a*]pyrimidine (**5b**):

^{1}H NMR (500 MHz, DMSO-d_6)

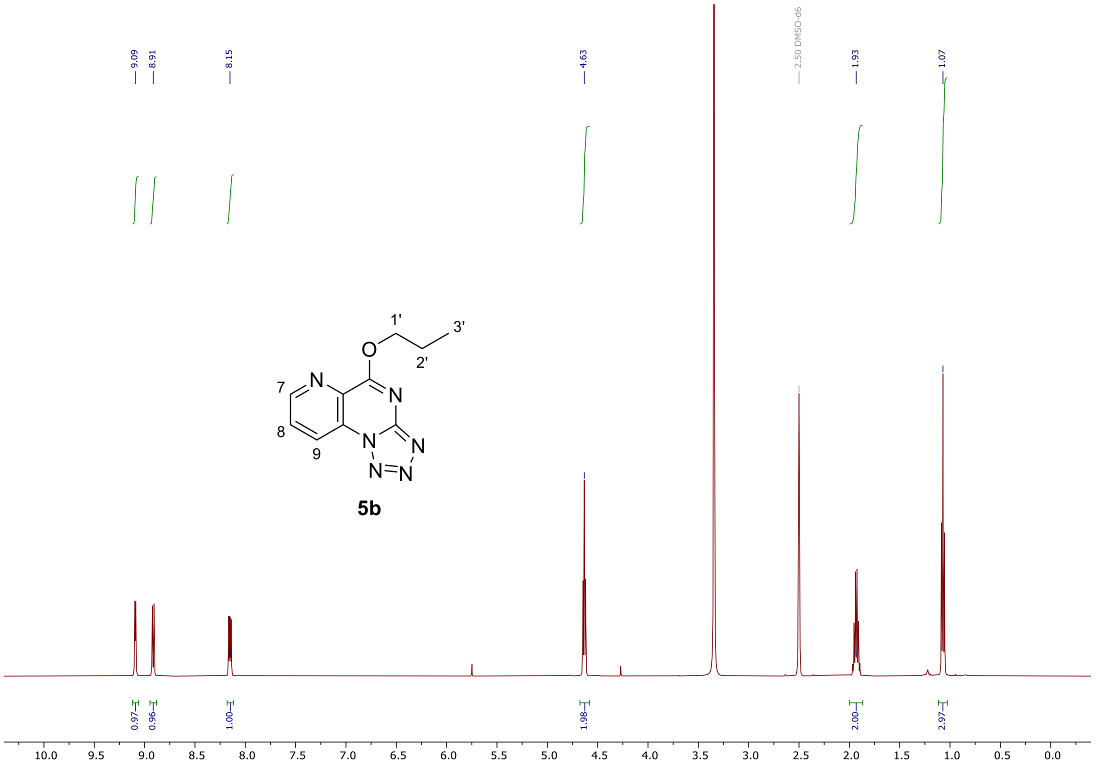

^{13}C NMR (125 MHz, DMSO-d_6)

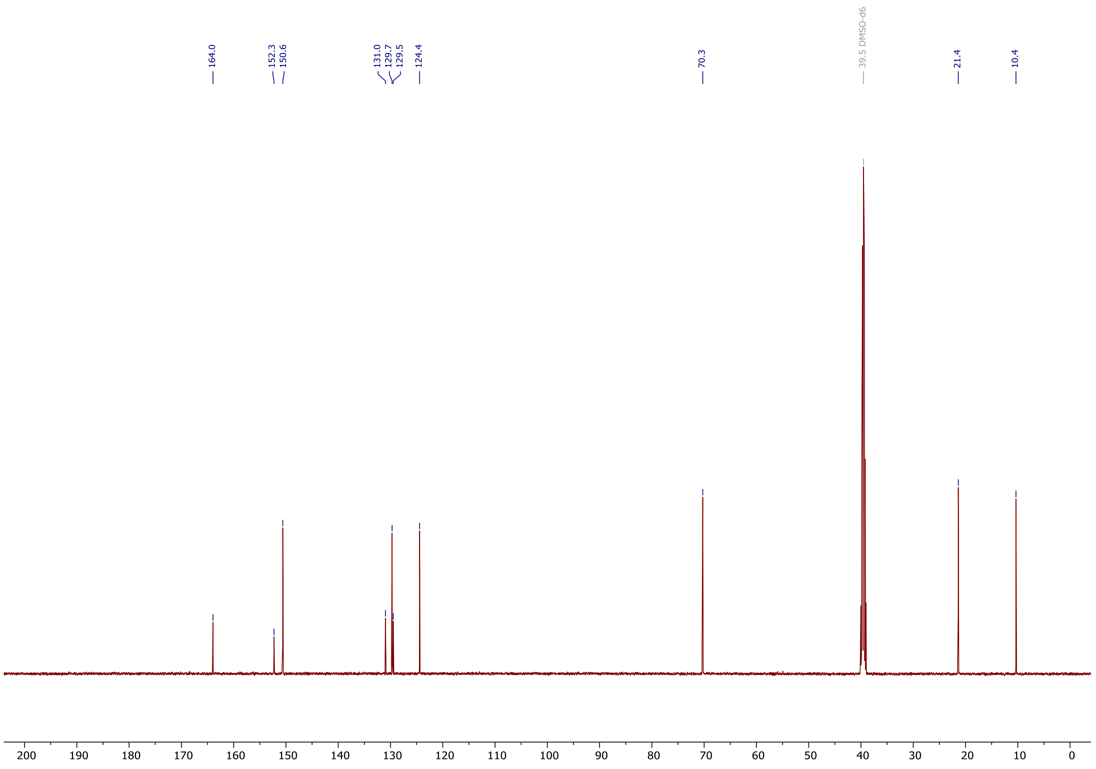

5-Propoxypyrido[2,3-*e*]tetrazolo[1,5-*a*]pyrimidine (**5b**) and 2-azido-4-propoxypyrido[3,2-*d*]pyrimidine (**5bA)**:

^{1}H NMR (500 MHz, $CDCl_3$)

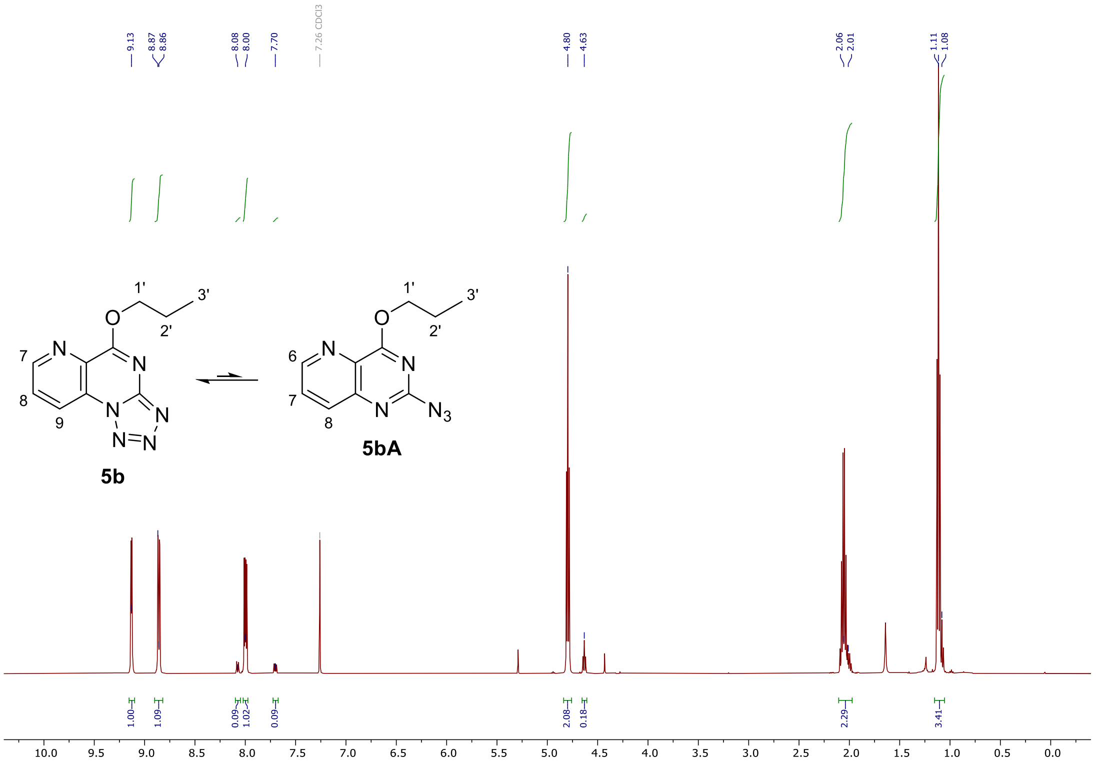

N-(4-Methoxybenzyl)-2-(4-phenyl-1*H*-1,2,3-triazol-1-yl)pyrido[3,2-*d*]pyrimidin-4-amine (**6a**):

^{1}H NMR (500 MHz, DMSO-d_6)

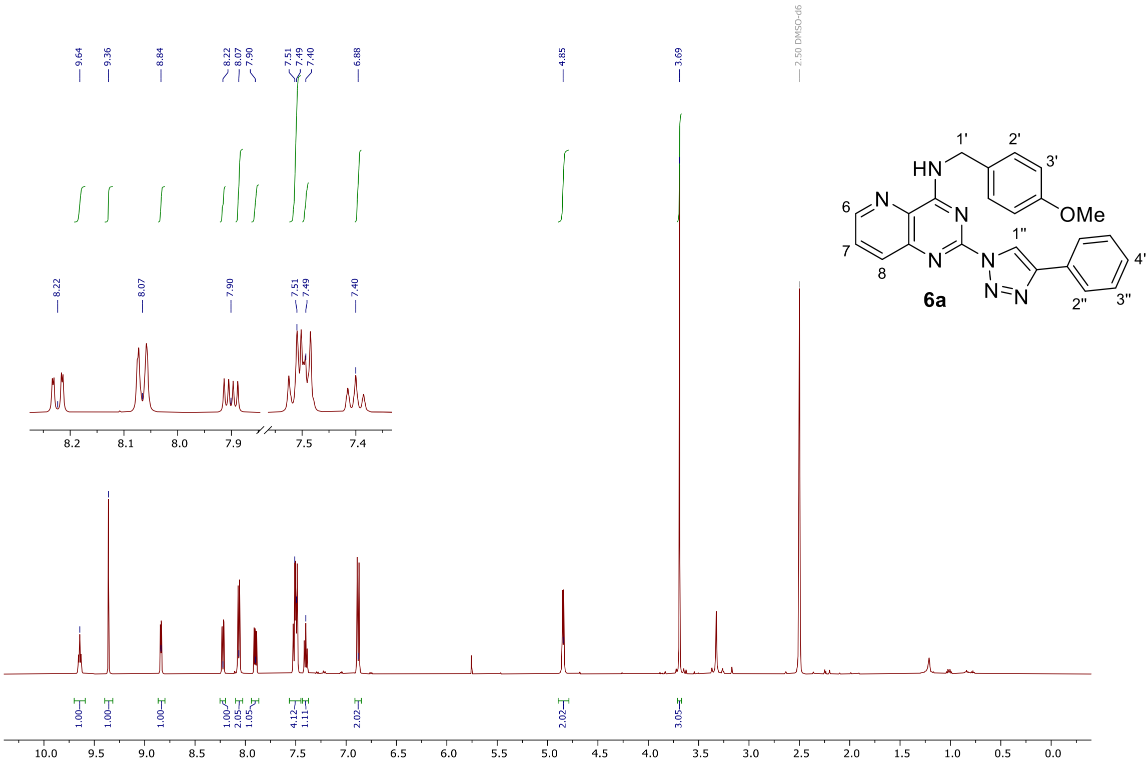

^{13}C NMR (125 MHz, DMSO-d_6)

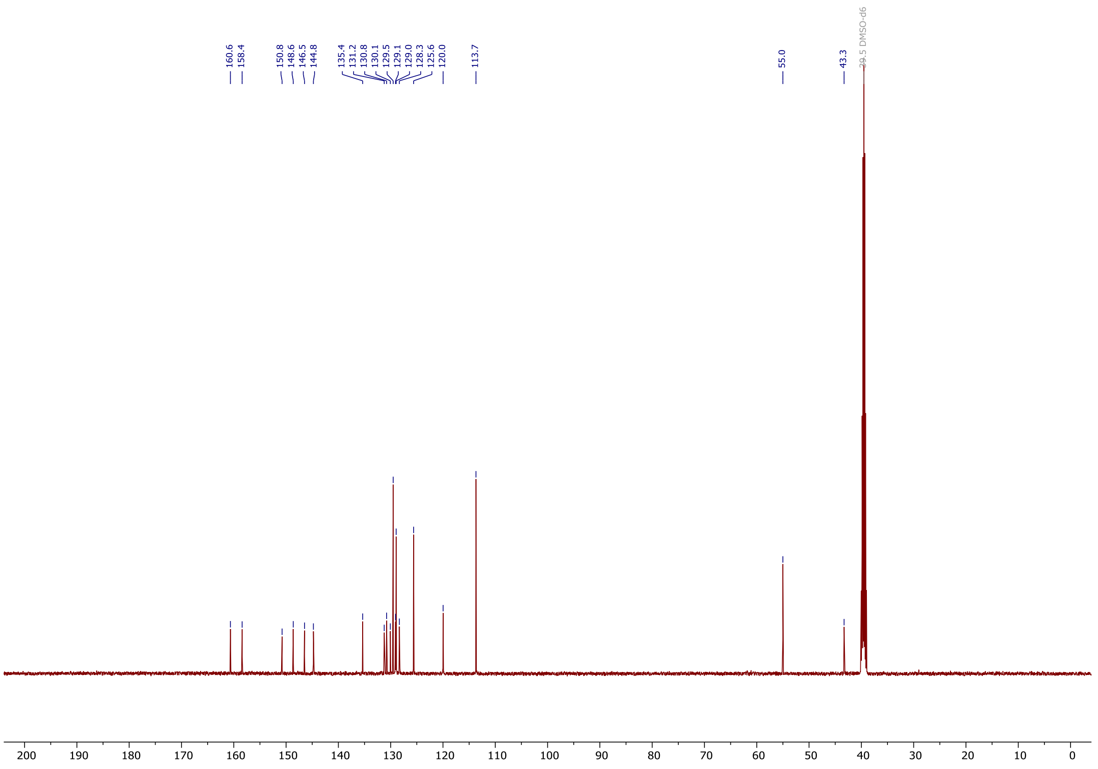

N-(4-Methoxybenzyl)-2-(4-phenyl-1*H*-1,2,3-triazol-1-yl)pyrido[3,2-*d*]pyrimidin-4-amine (**6a**):

^{1}H NMR (500 MHz, $CDCl_3$)

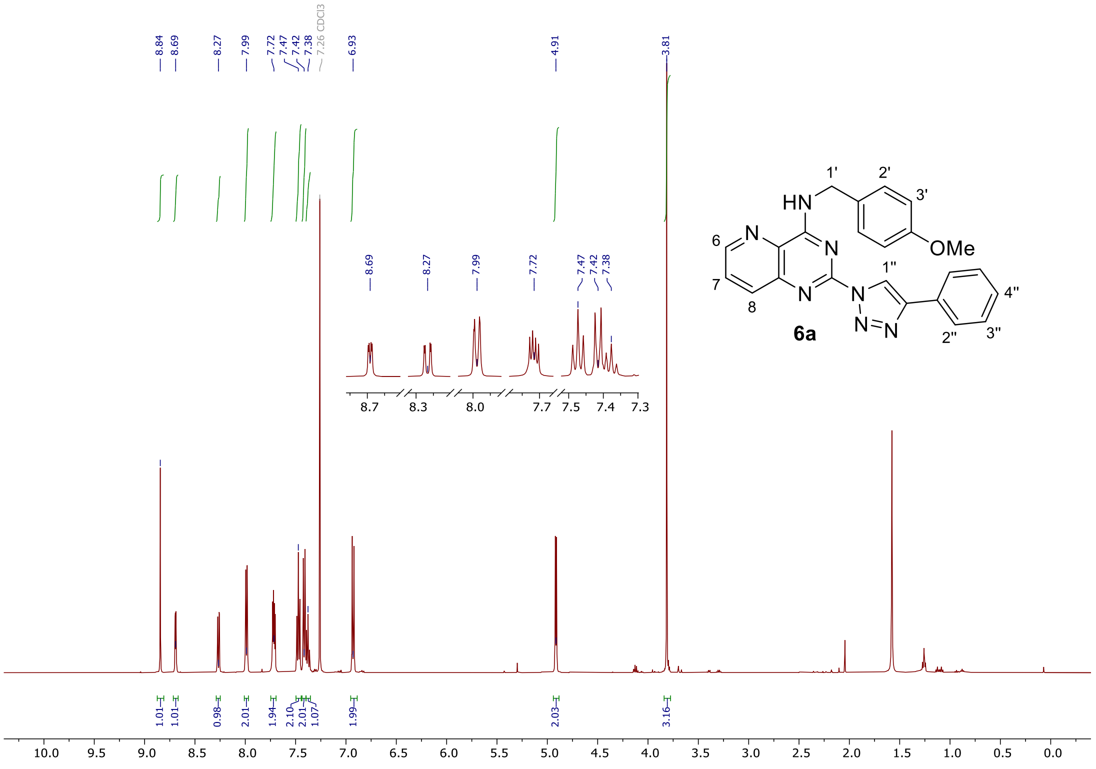

^{13}C NMR (125 MHz, $CDCl_3$)

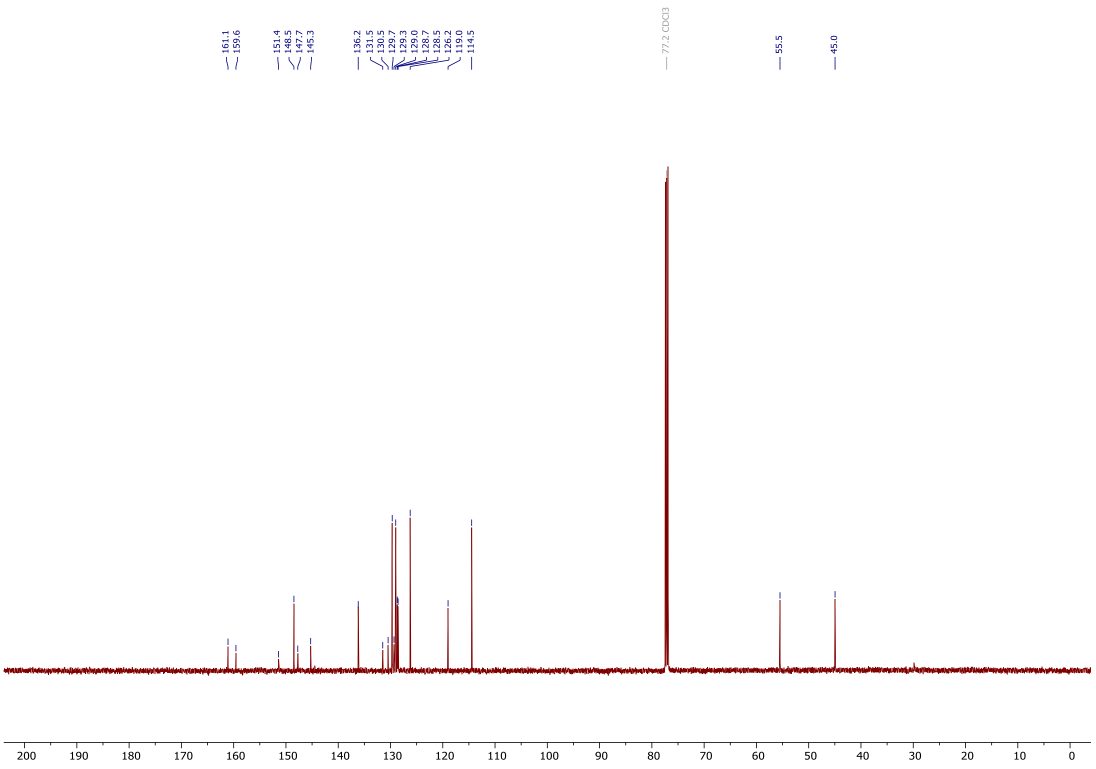

N-(4-Methoxybenzyl)-2-(4-(*p*-tolyl)-1*H*-1,2,3-triazol-1-yl)pyrido[3,2-*d*]pyrimidin-4-amine (**6b**):

^{1}H NMR (500 MHz, DMSO-d_6)

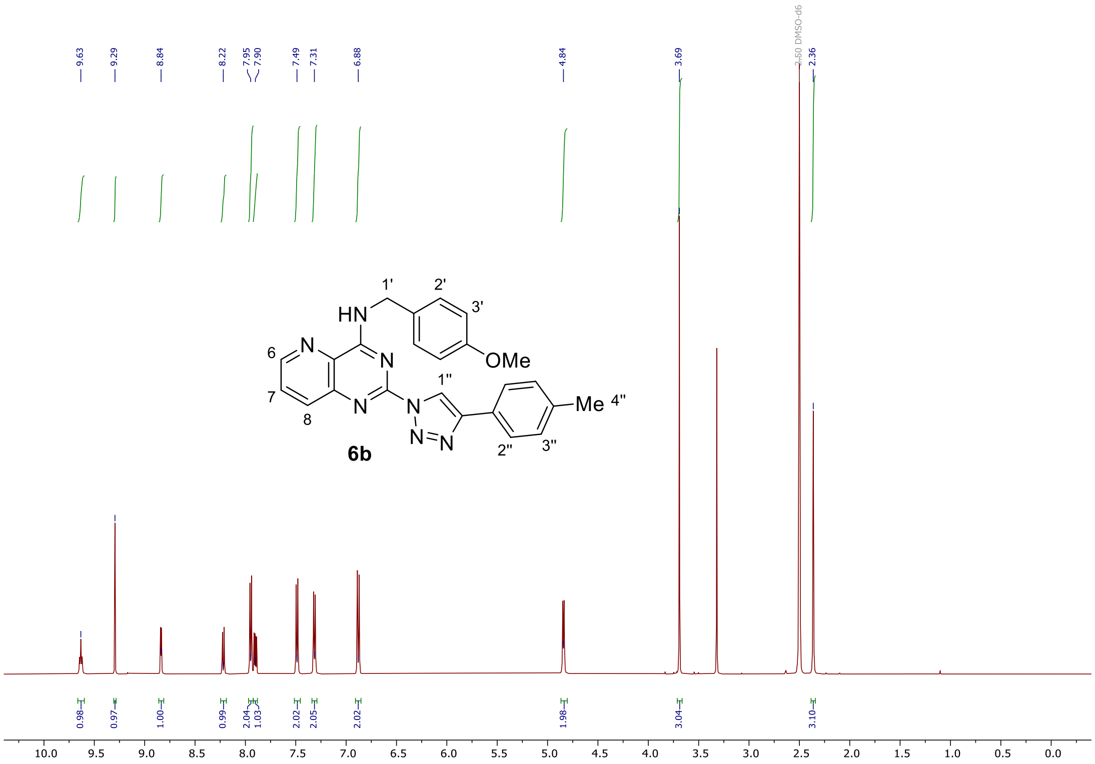

^{13}C NMR (125 MHz, DMSO-d_6)

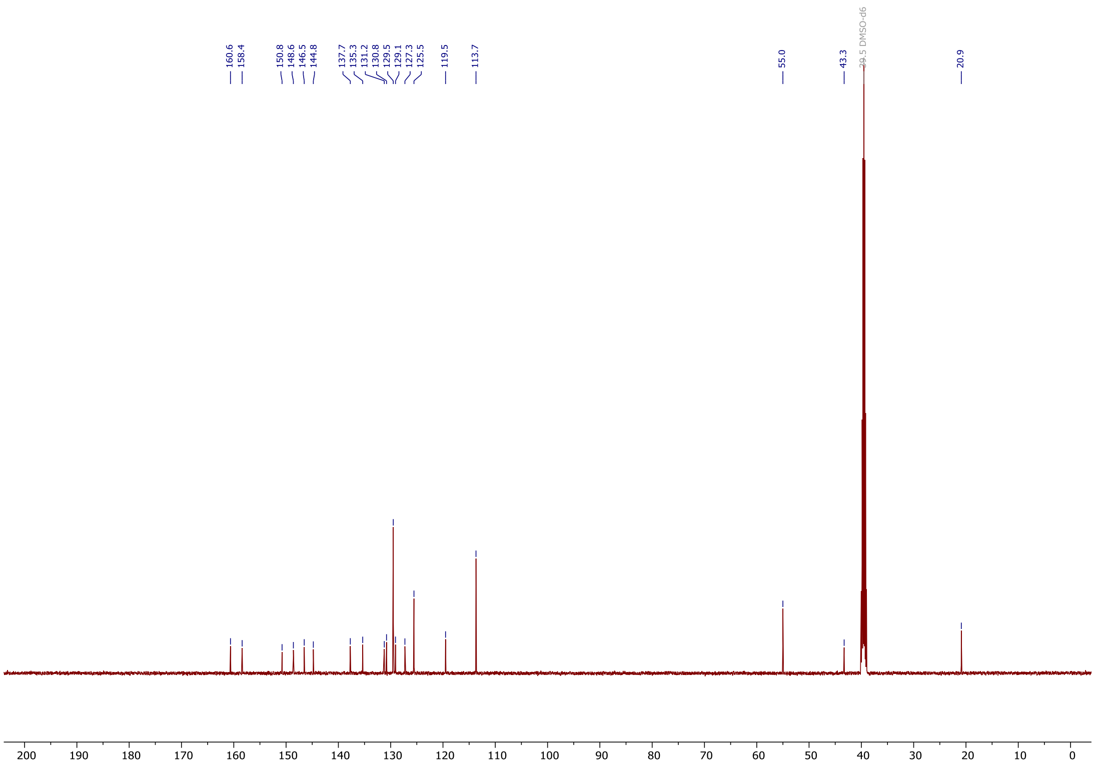

N-(4-Methoxybenzyl)-2-(4-(*p*-tolyl)-1*H*-1,2,3-triazol-1-yl)pyrido[3,2-*d*]pyrimidin-4-amine (**6b**):

^{1}H NMR (500 MHz, CDCl$_3$)

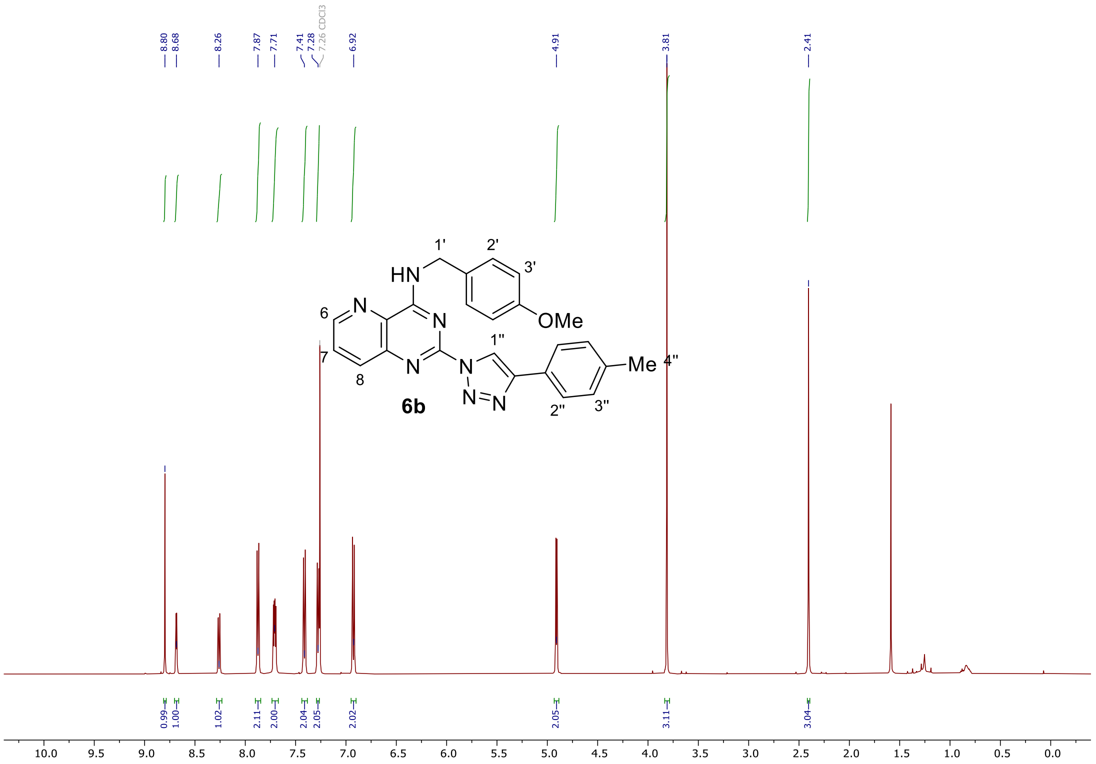

^{13}C NMR (125 MHz, CDCl$_3$)

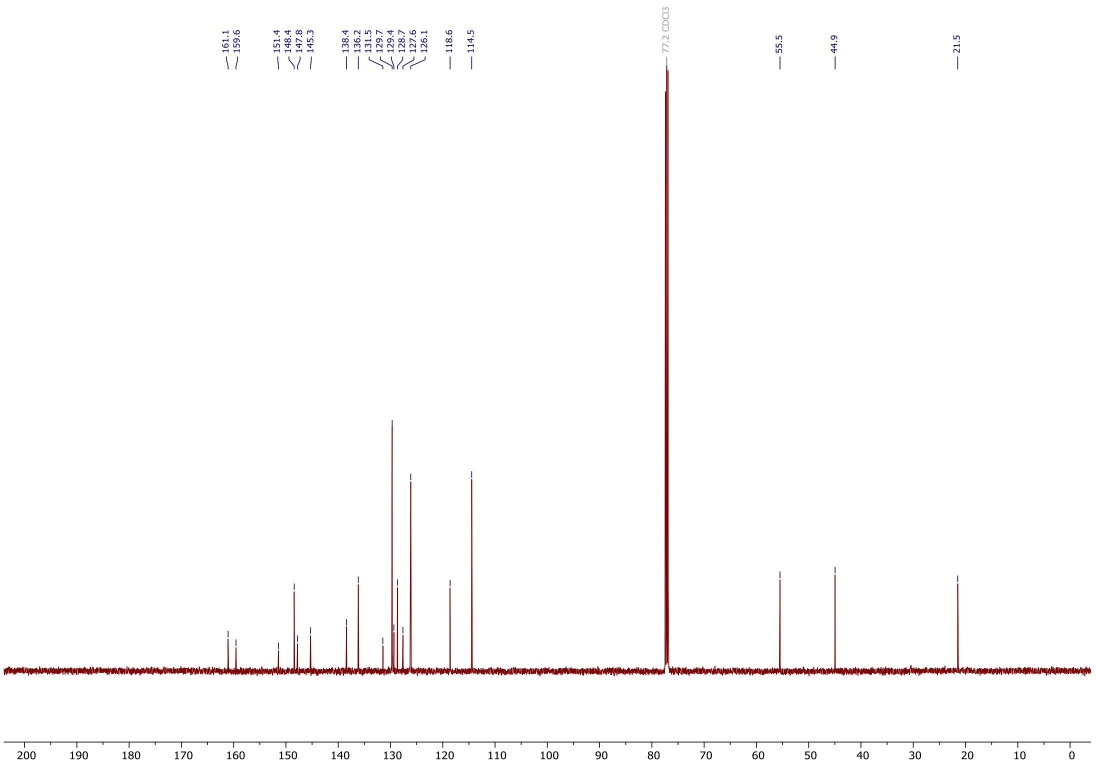

4-(1-(4-((4-Methoxybenzyl)amino)pyrido[3,2-*d*]pyrimidin-2-yl)-1*H*-1,2,3-triazol-4-yl)benzonitrile (**6c**):

^{1}H NMR (500 MHz, DMSO-d_6)

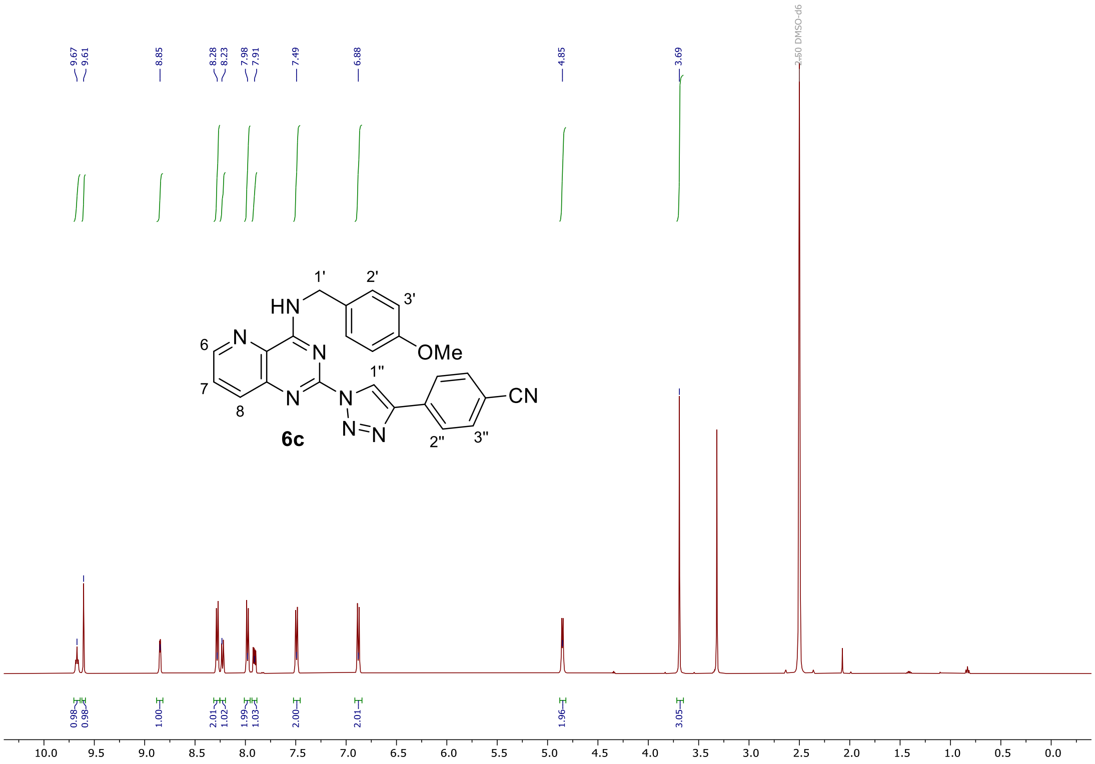

^{13}C NMR (125 MHz, DMSO-d_6)

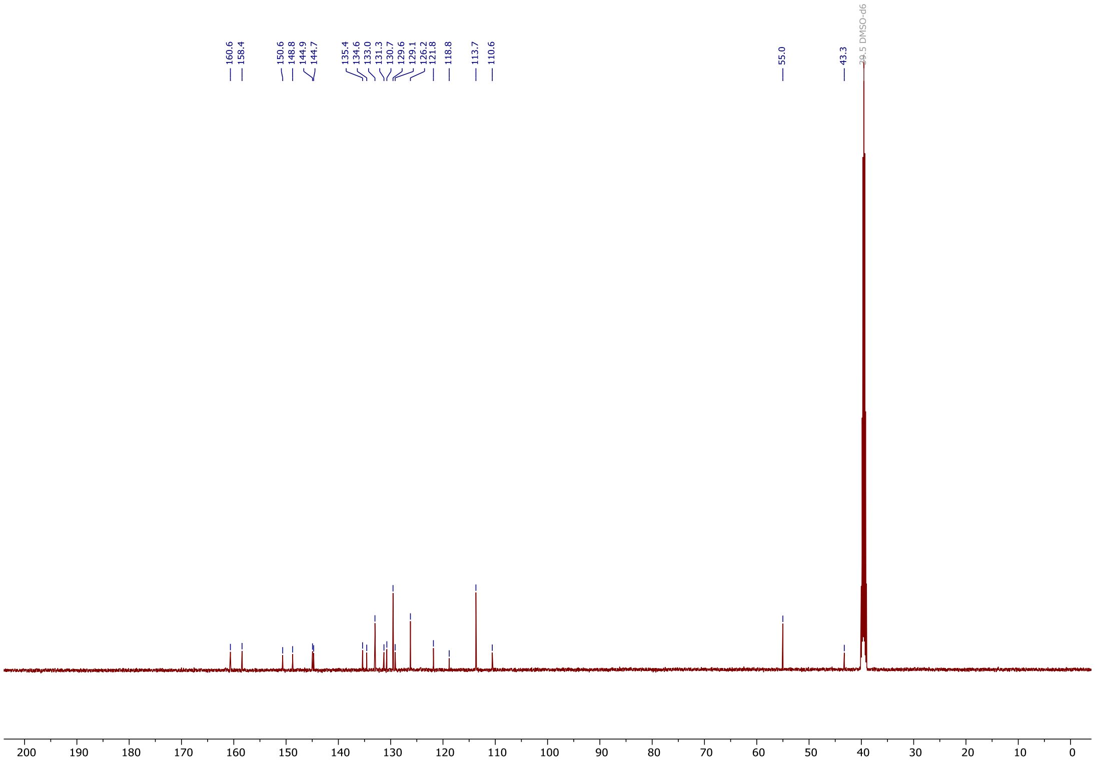

4-(1-(4-((4-Methoxybenzyl)amino)pyrido[3,2-*d*]pyrimidin-2-yl)-1*H*-1,2,3-triazol-4-yl)benzonitrile (**6c**):

^{1}H NMR (500 MHz, $CDCl_3$)

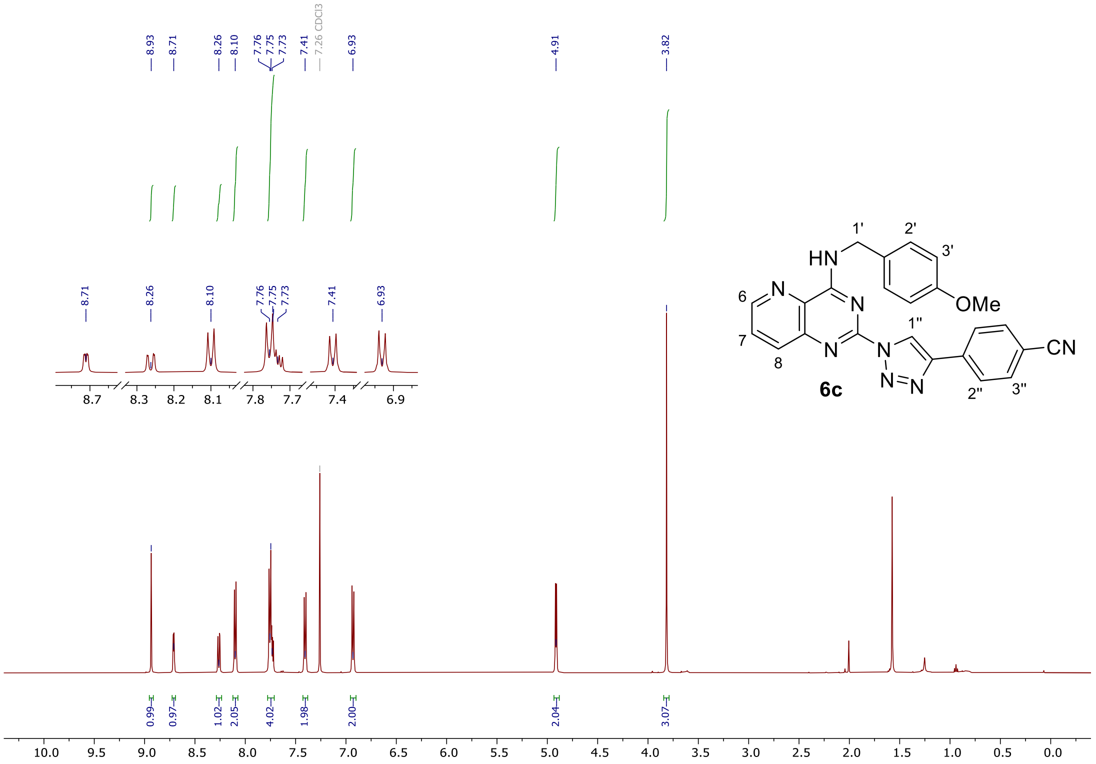

^{13}C NMR (125 MHz, $CDCl_3$)

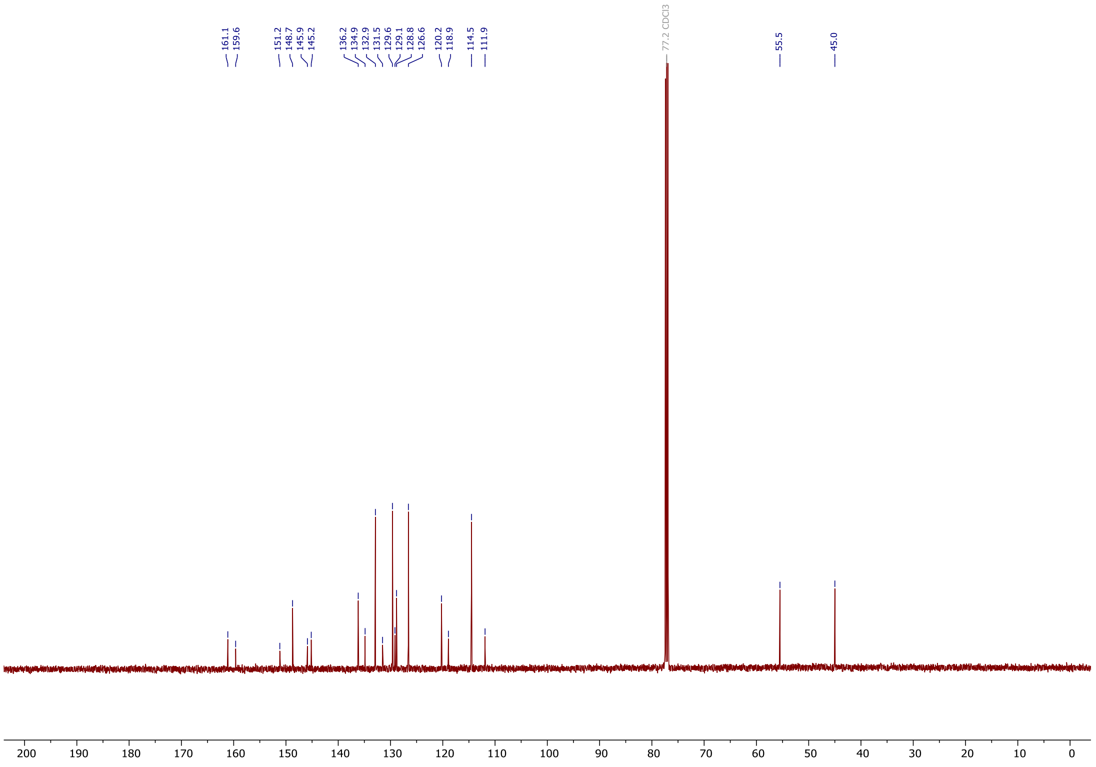

2-(4-Hexyl-1*H*-1,2,3-triazol-1-yl)-*N*-(4-methoxybenzyl)pyrido[3,2-*d*]pyrimidin-4-amine (**6d**):

^{1}H NMR (500 MHz, DMSO-d_6)

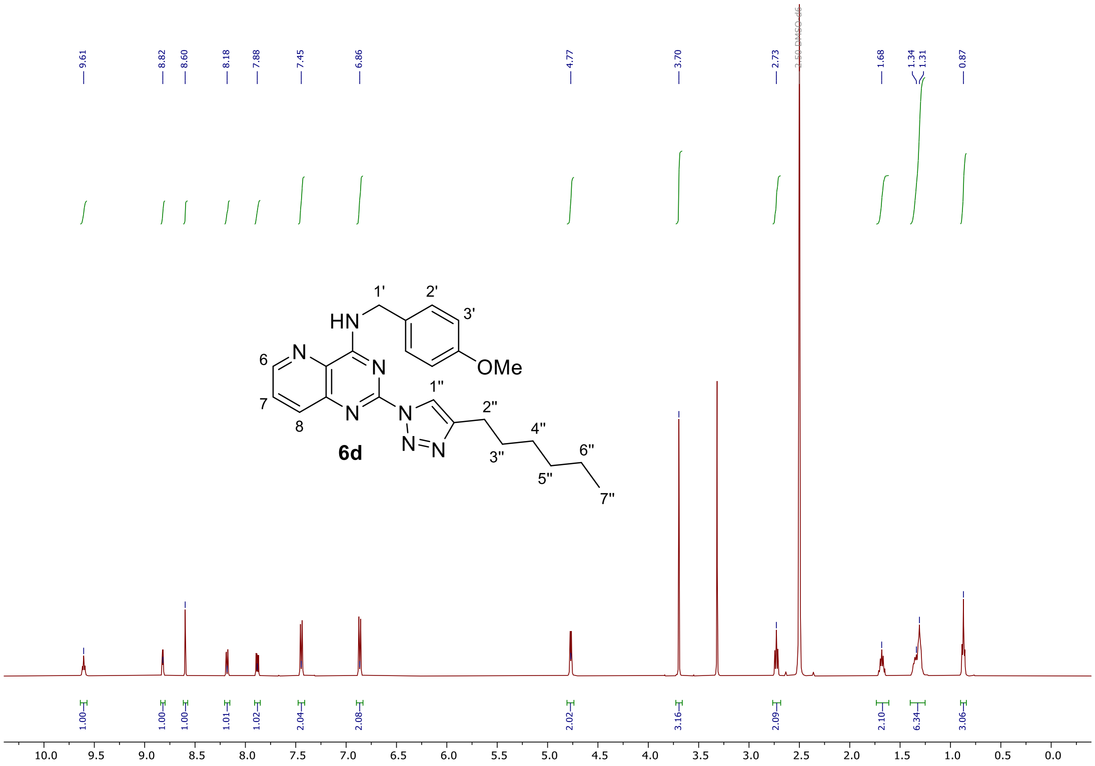

^{13}C NMR (125 MHz, DMSO-d_6)

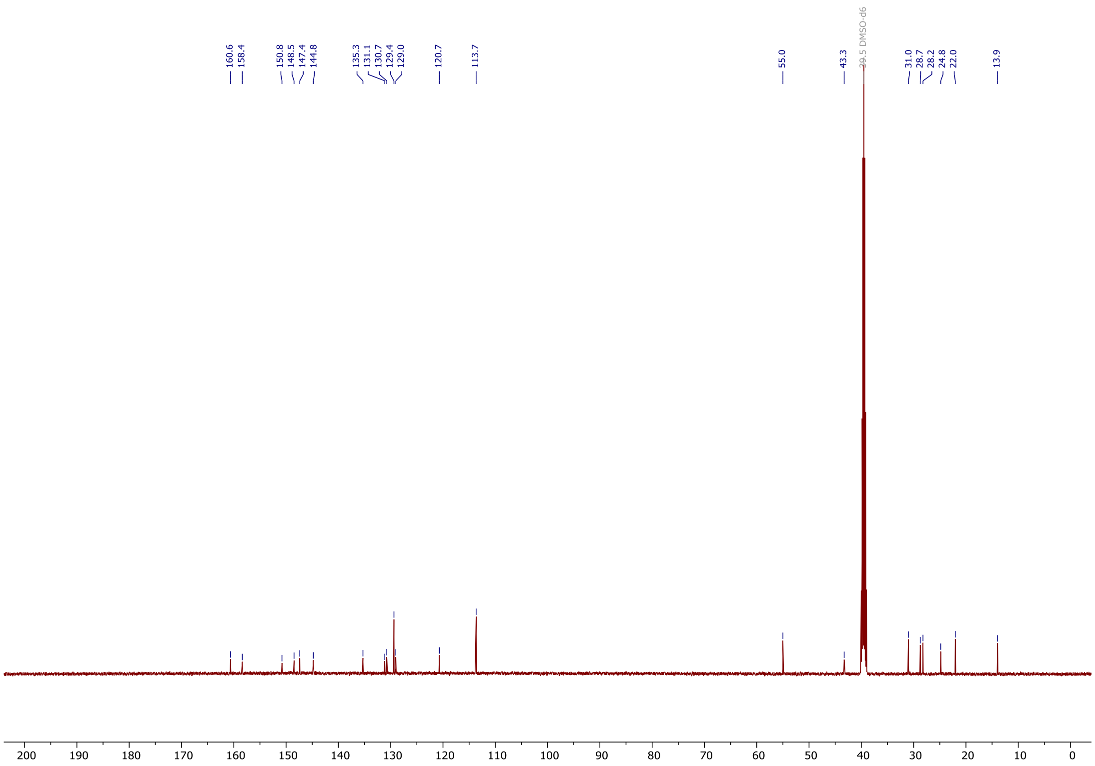

2-(4-Hexyl-1*H*-1,2,3-triazol-1-yl)-*N*-(4-methoxybenzyl)pyrido[3,2-*d*]pyrimidin-4-amine (**6d**):

^{1}H NMR (500 MHz, $CDCl_3$)

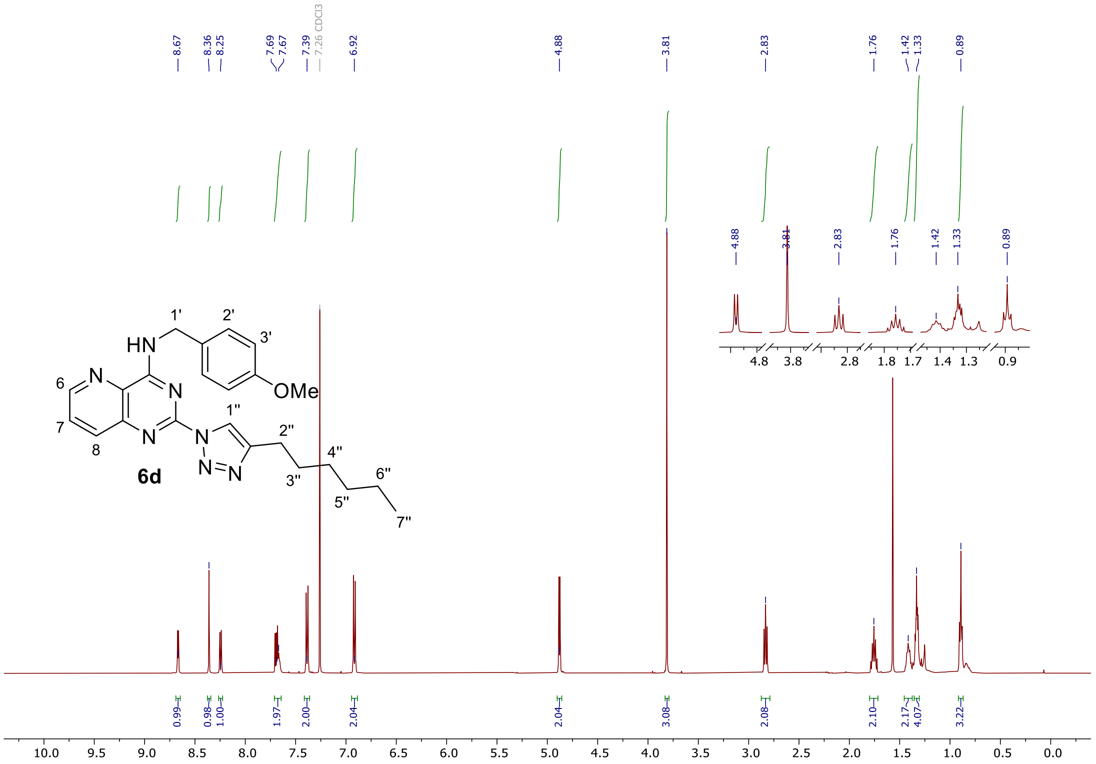

^{13}C NMR (125 MHz, $CDCl_3$)

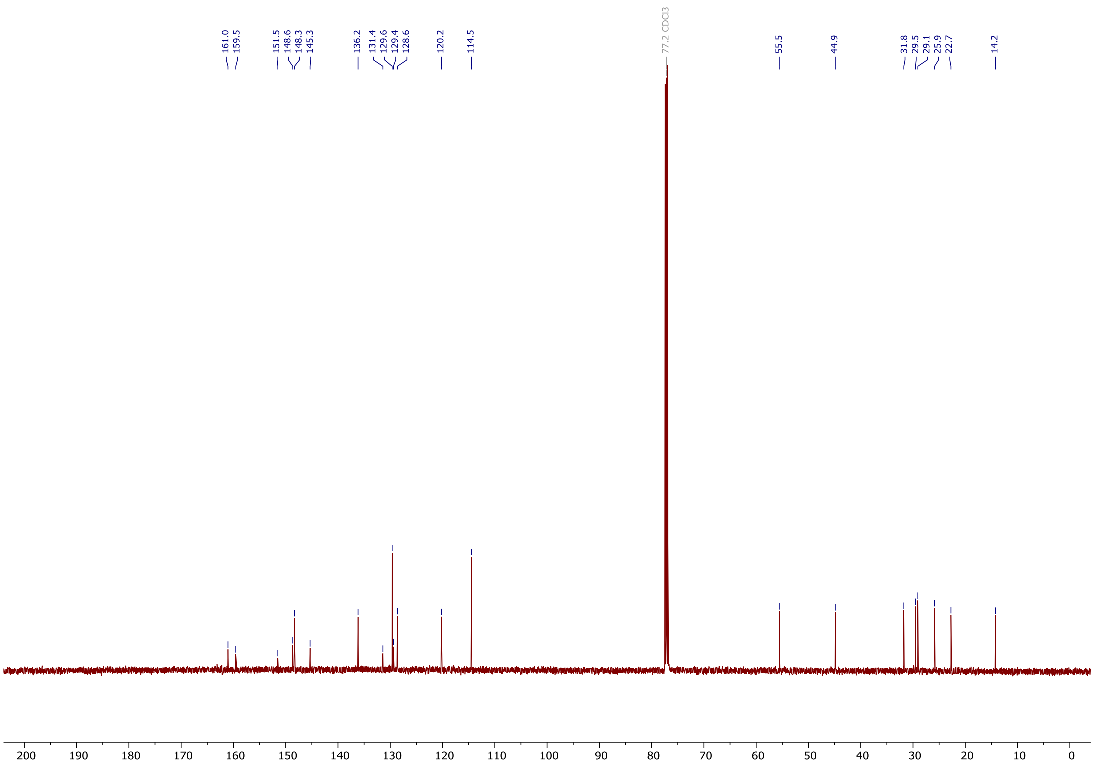

Methyl-1-(4-((4-methoxybenzyl)amino)pyrido[3,2-*d*]pyrimidin-2-yl)-1*H*-1,2,3-triazole-4-carboxylate (**6e**):

^{1}H NMR (500 MHz, $CDCl_3$)

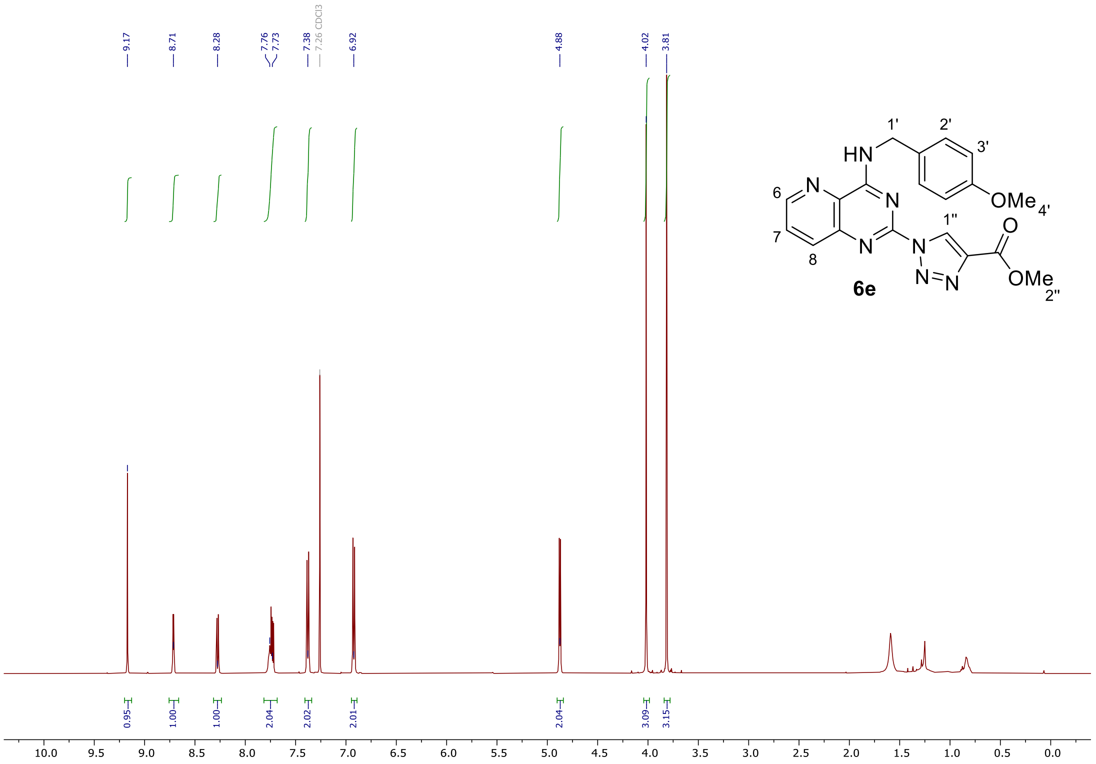

^{13}C NMR (125 MHz, $CDCl_3$)

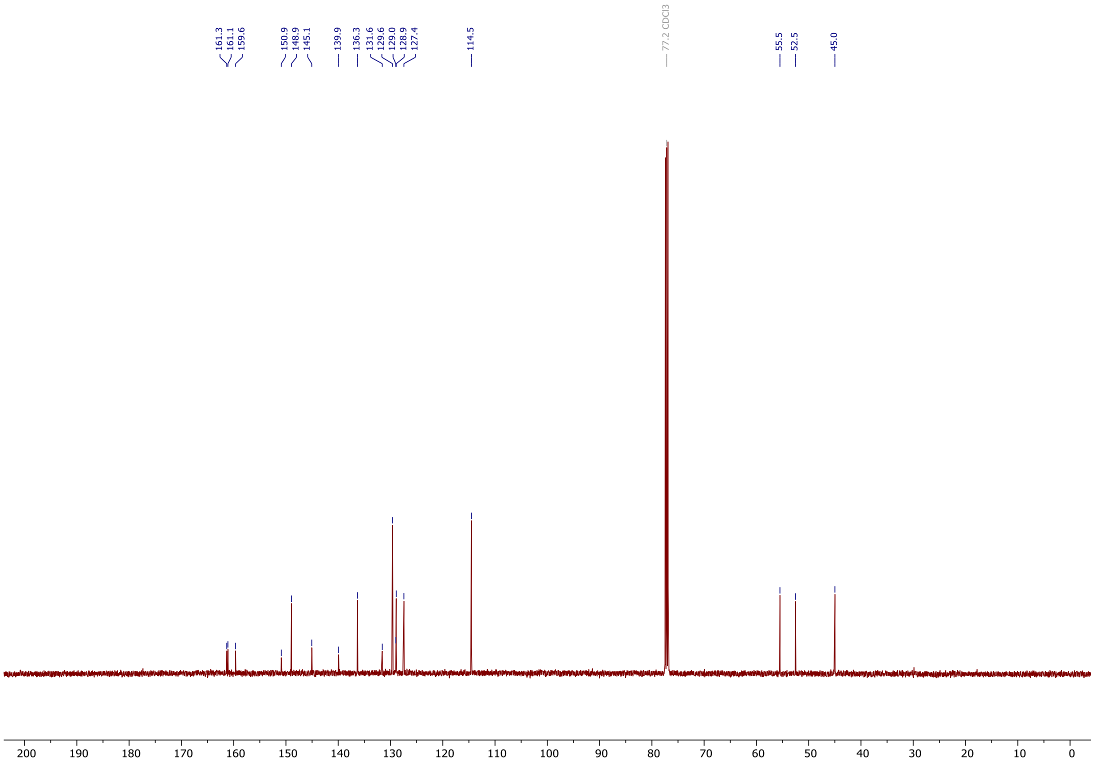

2-(4-Phenyl-1*H*-1,2,3-triazol-1-yl)pyrido[3,2-*d*]pyrimidin-4-amine (**7**):

^{1}H NMR (500 MHz, DMSO-d_6)

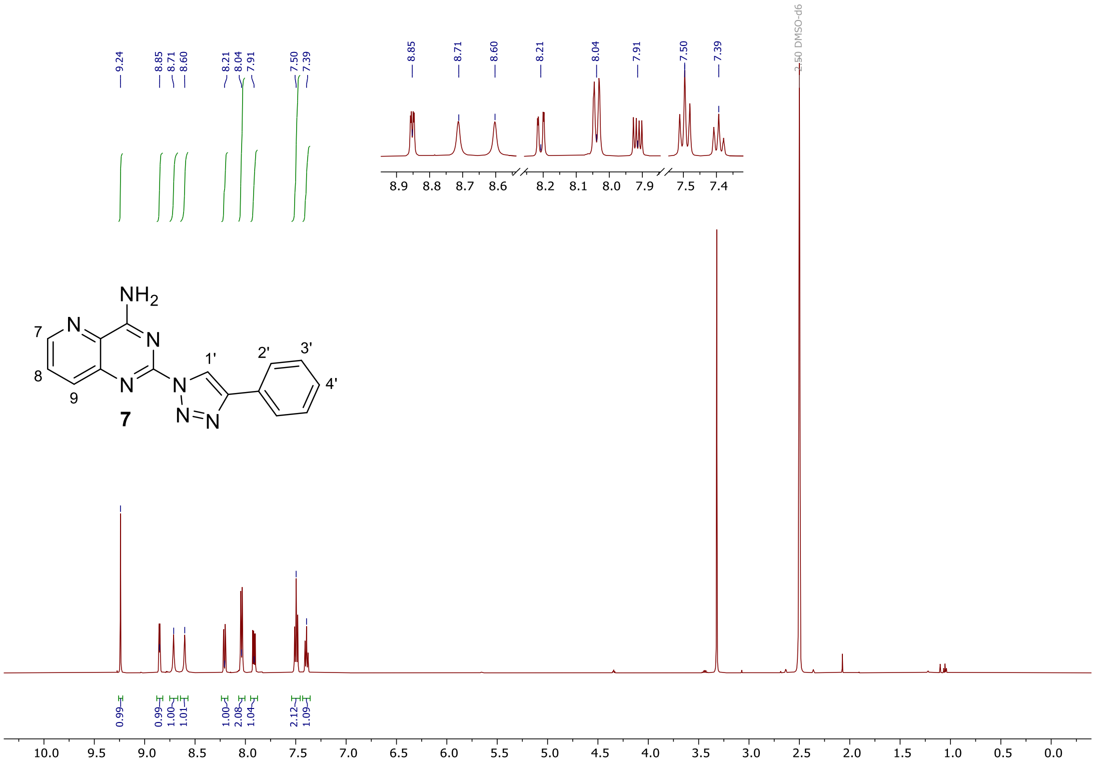

^{13}C NMR (125 MHz, DMSO-d_6)

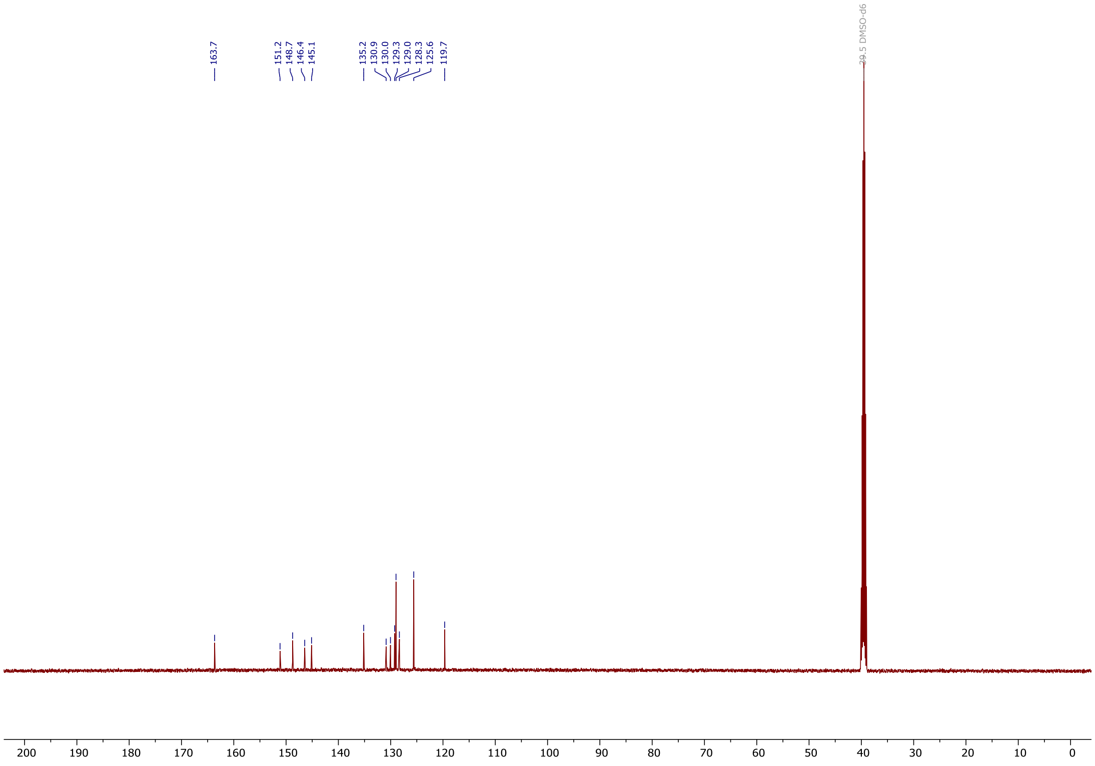

((4-(Hexylamino)pyrido[3,2-*d*]pyrimidin-2(1*H*)-ylidene)amino)triphenylphosphonium chloride (**8'**):

^{1}H NMR (500 MHz, DMSO-d_6)

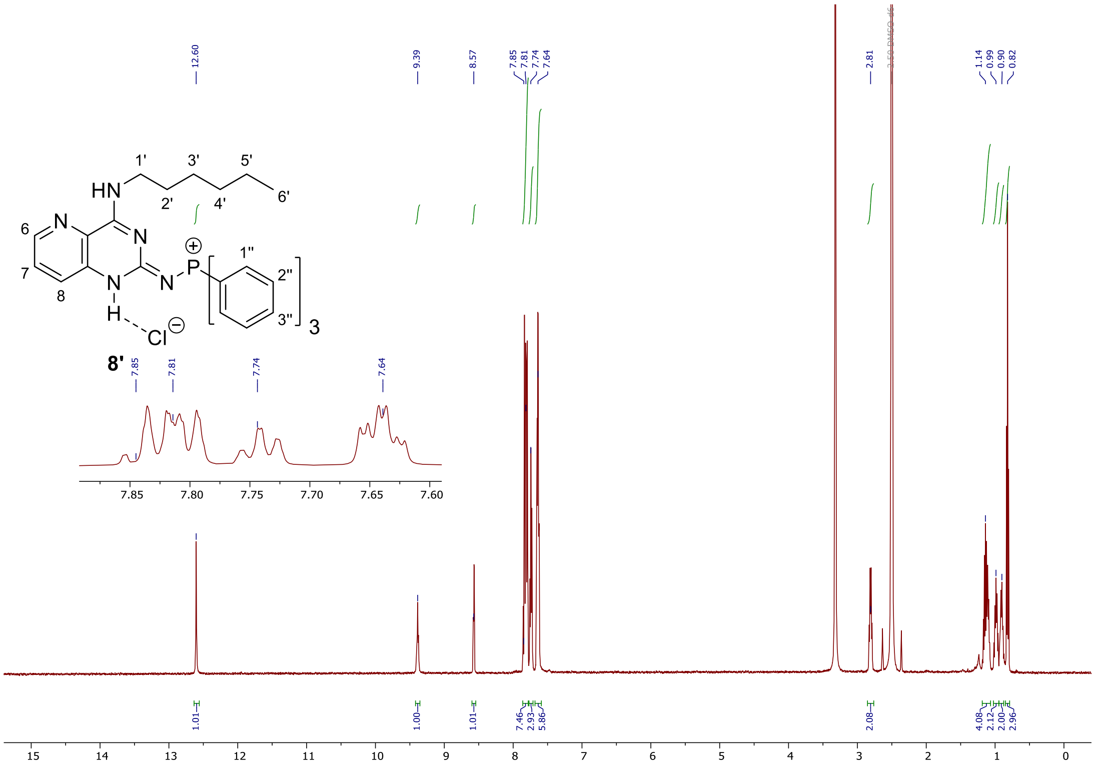

^{1}H NMR (500 MHz, CDCl$_3$)

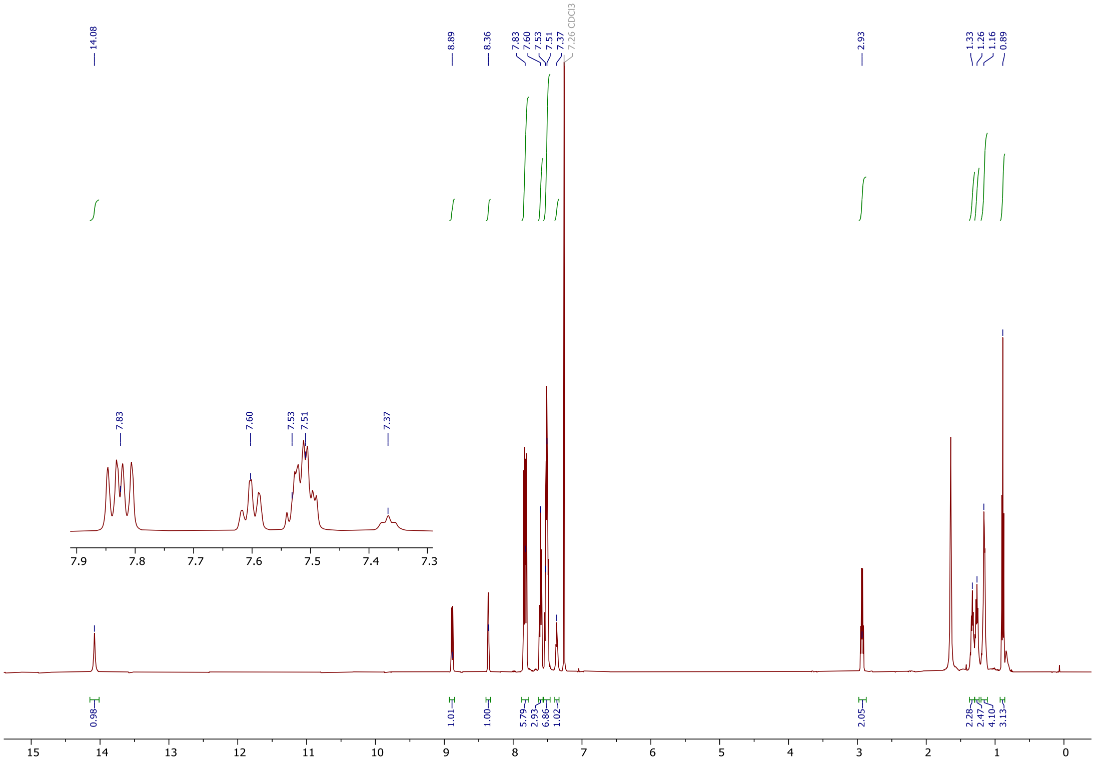

^{13}C NMR (125 MHz, $CDCl_3$)

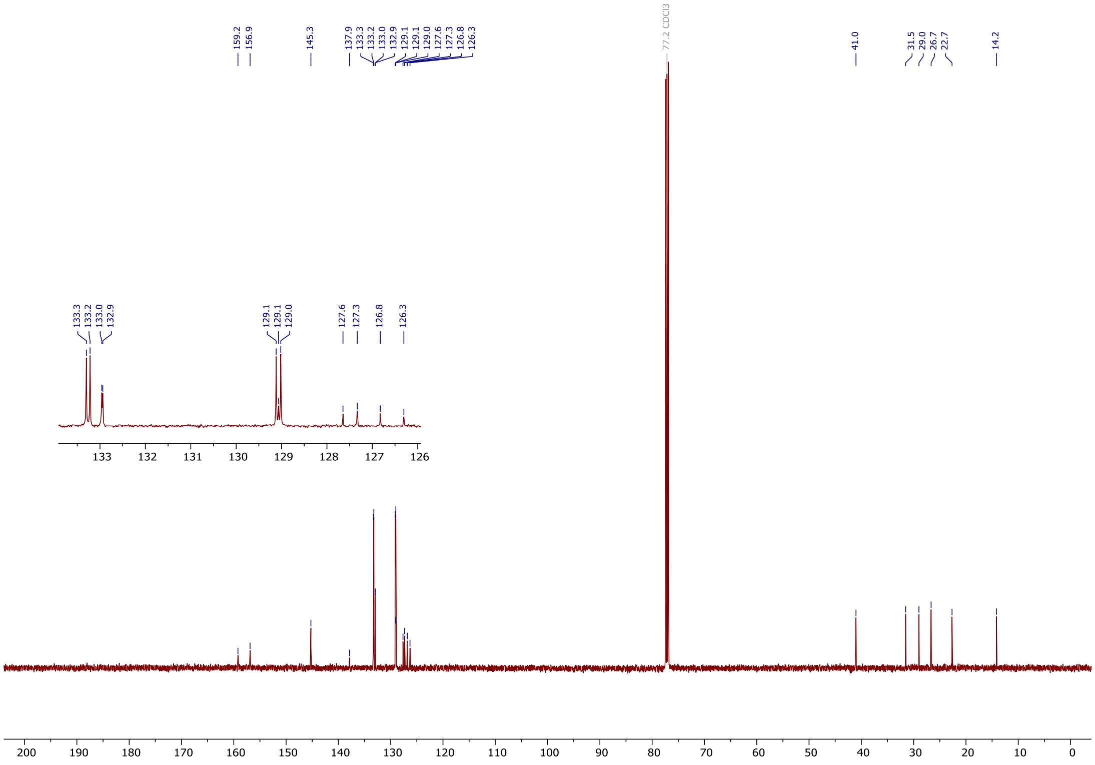

^{31}P NMR (202 MHz, $CDCl_3$)

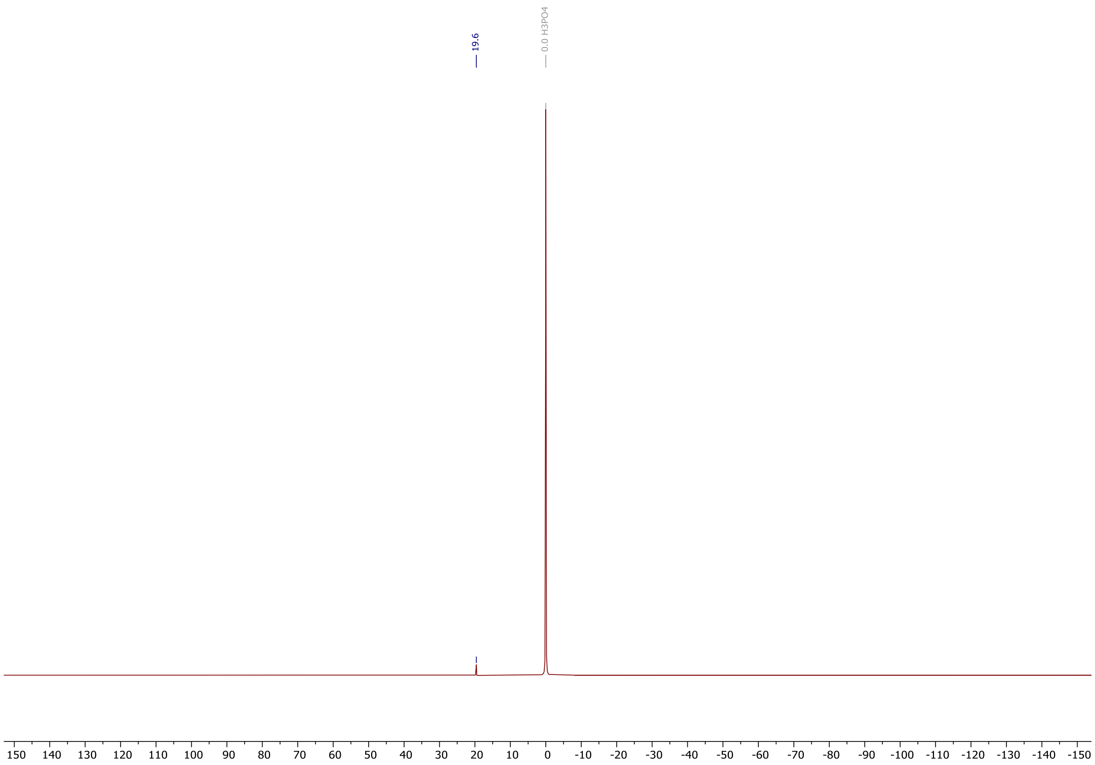

2-Chloro-*N*-hexylpyrido[3,2-*d*]pyrimidin-4-amine (**9**):

^{1}H NMR (500 MHz, $CDCl_3$)

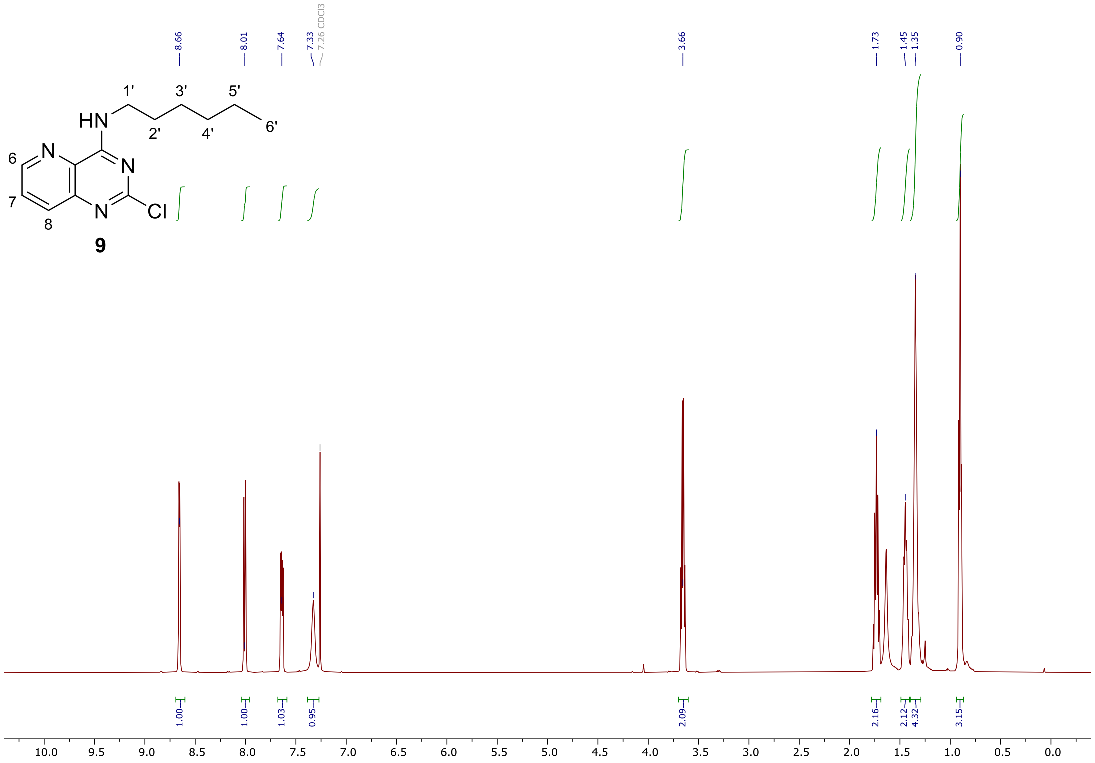

^{13}C NMR (125 MHz, $CDCl_3$)

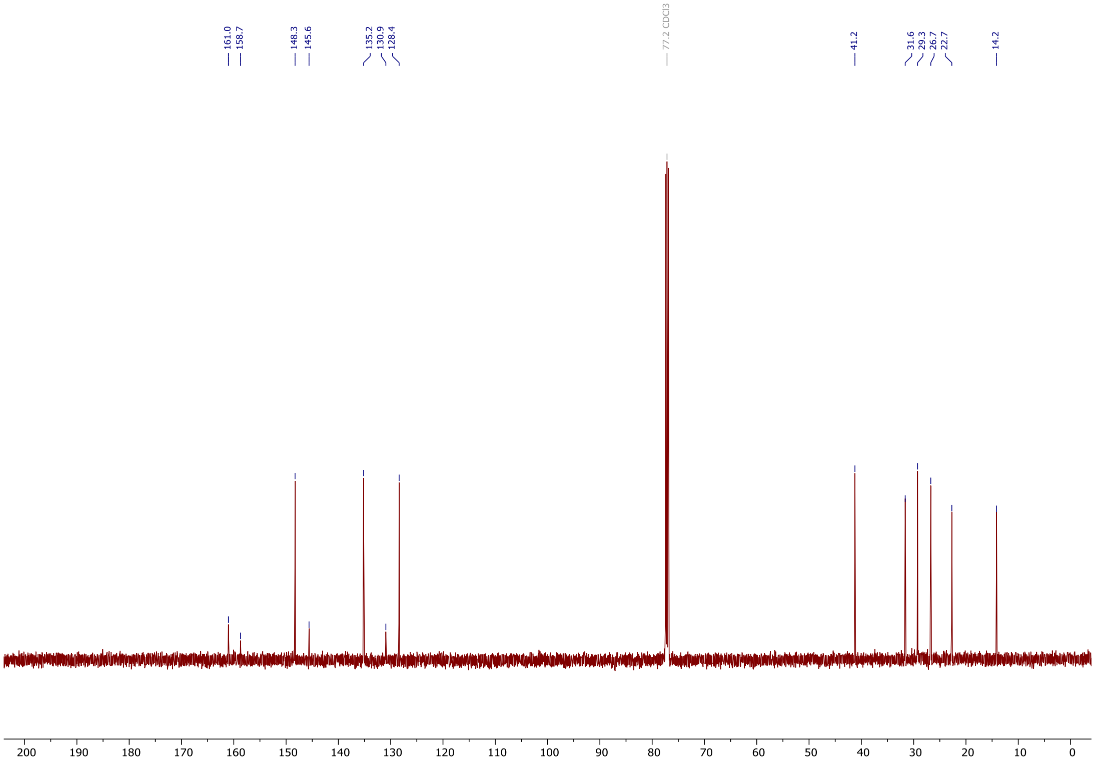

Supplement: Supplementary file 1 [file molecules-27-07675-s001.zip › molecules-2007556-supplementary.pdf]
